# Dissolved trace elements and nutrients in the North Sea—a current baseline

**DOI:** 10.1007/s10661-024-12675-2

**Published:** 2024-05-11

**Authors:** Anna Siems, Tristan Zimmermann, Tina Sanders, Daniel Pröfrock

**Affiliations:** 1 Institute of Coastal Environmental Chemistry, Helmholtz-Zentrum Hereon, Geesthacht, Germany; 2 Institute of Inorganic and Applied Chemistry, Department of Chemistry, Universität Hamburg, Hamburg, Germany; 3 Institute of Carbon Cycles, Helmholtz-Zentrum Hereon, Geesthacht, Germany

**Keywords:** ICP-MS, Trace metals, Skagerrak, Carbon storage, Primary production, Gadolinium anomaly

## Abstract

Primary production is an important driver of marine carbon storage. Besides the major nutrient elements nitrogen, phosphorus, and silicon, primary production also depends on the availability of nutrient-type metals (e.g., Cu, Fe, Mo) and the absence of toxicologically relevant metals (e.g., Ni, Pb). Especially in coastal oceans, carbon storage and export to the open ocean is highly variable and influenced by anthropogenic eutrophication and pollution. To model future changes in coastal carbon storage processes, a solid baseline of nutrient and metal concentrations is crucial. The North Sea is an important shelf sea, influenced by riverine, atmospheric, Baltic Sea, and North Atlantic inputs. We measured the concentrations of dissolved nutrients (NH_4_^+^, NO_3_^−^, PO_4_^3−^, and SiO_4_^4−^) and 26 metals in 337 water samples from various depths within the entire North Sea and Skagerrak. A principal component analysis enabled us to categorize the analytes into three groups according to their predominant behavior: tracers for seawater (e.g., Mo, U, V), recycling (e.g., NO_3_^−^, PO_4_^3−^, SiO_4_^4−^), and riverine or anthropogenic input (e.g., Ni, Cu, Gd). The results further indicate an increasing P-limitation and increasing anthropogenic gadolinium input into the German Bight.

**Supplementary Information:**

The online version contains supplementary material available at 10.1007/s10661-024-12675-2.

## Introduction

Primary production (PP) binds atmospheric carbon and serves as the basis of the global food web (Chavez et al., 2011) with a marine net PP fixing of roughly 50,000 teragrams (Tg) carbon per year (46% of global net PP; Field et al., 1998). While marine PP varies spatially, seasonally, and with global climate phenomena, model simulations indicate that global marine PP will decline in the future due to global warming (Chavez et al., 2011; Ito et al., 2010). However, because of their high productivity during summer, coastal oceans like the North Sea can still function as a sink for CO_2_, depending on latitude, weather, and circulation patterns (Bozec et al., 2005; Cai & Dai, 2004; Chaichana et al., 2019; Emeis et al., 2015; Macovei et al., 2021; Thomas et al., 2004).

Primary producers rely on the availability of nutrient elements (nitrogen (N), phosphorus (P), and silicon (Si)) in a certain ratio (Redfield et al., 1963). Depending on the availability of P and N species, the system usually is either P- or N-limited (Burson et al., 2016). Additionally, organisms need trace elements like cobalt (Co), copper (Cu), cadmium (Cd), iron (Fe), manganese (Mn), and nickel (Ni) for the synthesis of enzymes related to nutrient and carbon uptake or nitrogen sequestration (Croot et al., 2002; Morel, 2008; Morel & Price, 2003; Pinedo-González et al., 2015; Schulz et al., 2004). However, increased metal concentrations can also hamper phytoplankton growth (Echeveste et al., 2012; Yang et al., 2019), and the effects of recently introduced new anthropogenic metal contaminants like gadolinium (Gd) or other rare earth elements are still not sufficiently investigated. However, first studies hint at the negative impacts of Gd on marine organism metabolism (Trapasso et al., 2021). Hence, the available elements and their concentrations might strongly impact the phytoplankton community structure and productivity (Sunda, 2012).

**Fig. 1:**
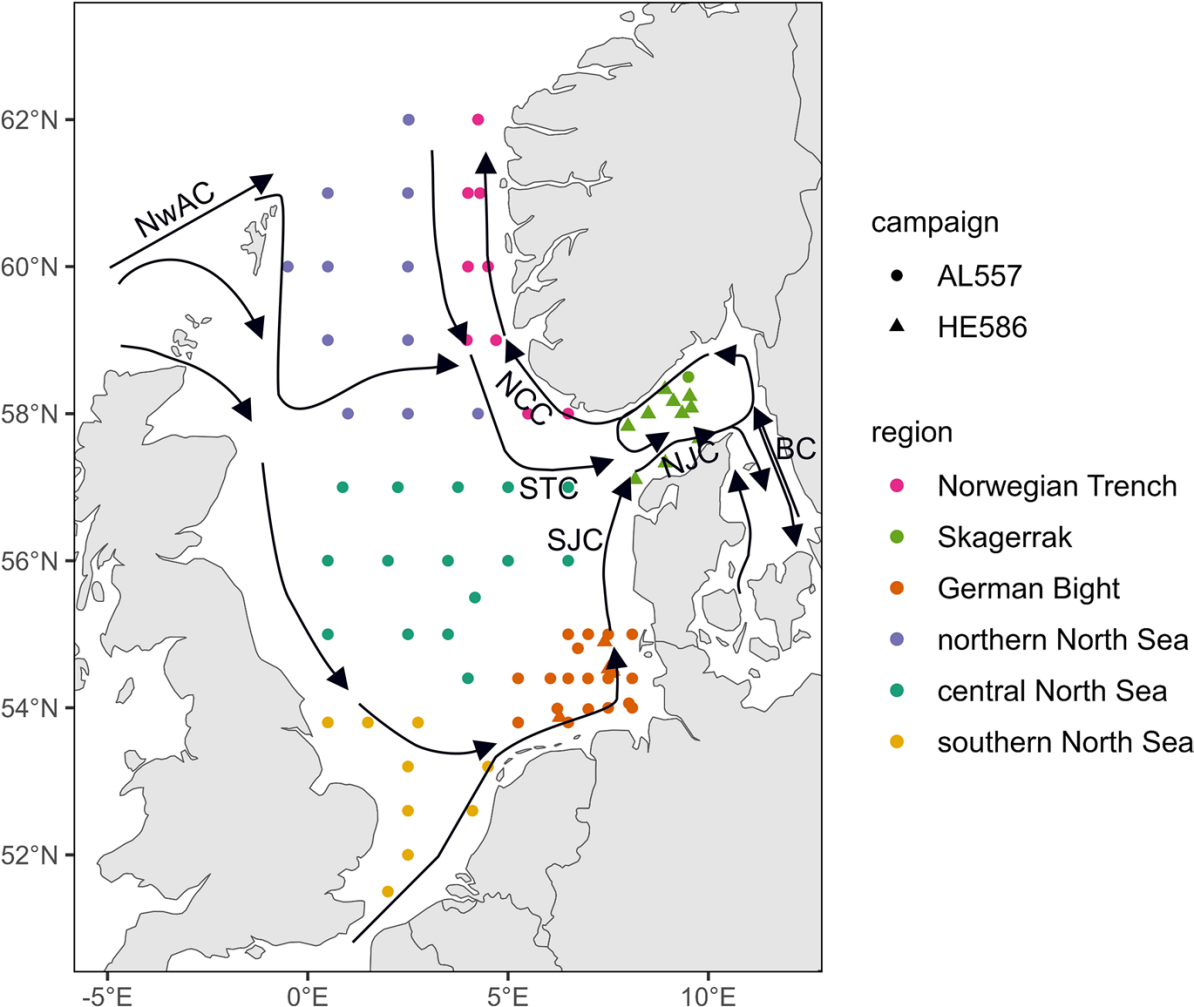
Map of sampling area, currents, and sampling stations in the North Sea. Currents are NwAC (Norwegian Atlantic Current), NCC (Norwegian Coastal Current), STC (Southern Trench Current), SJC (Southern Jutland Current), NJC (Northern Jutland Current), and BC (Baltic Current). Currents are taken from Brückner and Mackensen (2006). Station numbers are given in Fig. S1

The North Sea is an economically important shelf sea, exposed to continuous anthropogenic eutrophication and contamination (Chaichana et al., 2019; Emeis et al., 2015; Fock, 2003; Hickel et al., 1993; Radach & Patsch, 2007). It is characterized by anti-clockwise currents (Fig. 1), water inflow of North Atlantic water through the northern border and the English Channel, as well as the Baltic Sea inflow via the Skagerrak and outflow of water via the Norwegian trench (Fig. 1; Thomas et al., 2005; Winther & Johannessen, 2006). The shallow southern North Sea is defined by a mixed water column in which the production and respiration of organic matter take place in parallel (Thomas et al., 2004). The deeper northern North Sea is characterized by a stratified water column with PP in the surface waters and respiration in the subsurface waters which export approximately 2.2 Tg C month^−1^ to the North Atlantic during late summer (Bozec et al., 2005; Thomas et al., 2004). Nutrients and trace elements are supplied to the North Sea mainly by the inflow of nutrient-rich water from the North Atlantic and riverine inputs (Chaichana et al., 2019; Emeis et al., 2015; Pätsch et al., 2004; Topcu et al., 2011a, 2011b). In addition, atmospheric deposition can play an important role in nutrient and metal inputs (de Leeuw et al., 2003; Jickells et al., 2016; Pätsch & Kühn, 2008). The open North Sea is rather N-limited while the coastal North Sea is mostly P-limited due to high riverine N-inputs (Burson et al., 2016).

Gröger et al. (2013) simulated that decreasing anthropogenic eutrophication and climate-change-dependent change in ocean circulations will reduce the nutrient supply and subsequently reduce the PP in the North Sea by up to 35% during the 21st century. Although the North Sea is currently a sink for CO_2_ (Thomas et al., 2004), decreased inputs of trace elements and nutrients might significantly influence PP, carbon fixation capabilities, and ultimately also its export to the North Atlantic and long-term carbon storage. To investigate and model these biological and physical processes for the North Sea (e.g., Lenhart et al., 2004; Radach & Lenhart, 1995), trace element and nutrient concentrations with a high spatial resolution (longitude, latitude, depth) are needed, since these analytes feature a high seasonal and year-to-year variability (BSH, 2016; Fock, 2003). However, only a few studies on nutrient concentrations for the entire North Sea with high-depth resolutions are available (e.g., Bozec et al., 2005; Chaichana et al., 2019). For trace elements, transects through the North Sea were sampled during the 1980s and 1990s (e.g., Hydes & Kremling, 1993; Laslett, 1995; Tappin et al., 1995). Recent data for these parameters is often only available for coastal regions, e.g., the German Bight (BSH, 2016; Raabe & Wiltshire, 2009; Topcu et al., 2011a, 2011b), Southern Bight (Gao et al., 2013; Lefebvre & Dezecache, 2020; Mortelmans et al., 2019; van der Zee & Chou, 2005), or Skagerrak (Croot et al., 2002; Frigstad et al., 2013; Frigstad et al., 2020).

We aim to provide a comprehensive data set of dissolved nutrients (ammonium (NH_4_^+^), nitrite (NO_2_^−^,), nitrate (NO_3_^−^), phosphate (PO_4_^3−^), silicate (SiO_4_^4−^)), and 26 dissolved trace elements (e.g., Co, Cu, Cd, Fe) for the entire North Sea which can be used as a baseline for models and to track future concentration changes. Most samples were taken in June, and the Skagerrak and German Bight were also sampled in October. We are aware that seasonality affects the results but aimed at providing a first data set that should be supplemented by further data sets in the future. By applying principal component analysis (PCA), we were able to divide the analytes into three groups, depending on their major behavior: marine tracers with conservative mixing, nutrient-like analytes, and analytes with anthropogenic or terrestrial sources. We have used these groups to understand the impact of the investigated analytes on PP and remineralization as important factors for carbon storage.

## Materials and methods

### Sampling

The water samples were taken during two cruises in June and October 2021. The cruise in June covered the entire North Sea (RV Alkor, AL557), while the cruise in October (RV Heincke, HE586) focused on the Skagerrak and the German and Danish coast. Sampling stations are shown in Fig. 1, additional information on locations, water depths, bottom depths, and sampling dates is given in Table S1 and Fig. S1. The water samples were taken at several depths with Niskin bottles attached to a CTD (conductivity, temperature, depth sensor). The ship was positioned against the currents, and the Niskin bottles were rinsed with ambient water during the downcast. For trace element analysis, the water was directly filled into two acid-leached 0.5-L bottles (LDPE, Nalgene^TM^, Thermo Scientific^TM^, USA) or one acid-leached 1-L bottle (LDPE, Brand, Germany) during AL557 and HE586, respectively. Samples were frozen immediately (−20 °C) onboard the ship. The samples were thawed the night before filtration. Under clean room conditions, triplicates were filtered with *Digi*FILTER^TM^s (0.45 µm, 50 mL, SCP Science, Canada) into *Digi*TUBE^TM^s from which they were measured (Przibilla et al., 2023). *Digi*FILTER^TM^s and *Digi*TUBE^TM^s were cleaned for 1 week with diluted acid (0.1% (*w*/*w*) HCl and 1.3% (*w*/*w*) HNO_3_), respectively; Przibilla et al., 2023). The sampling bottles (1 L, LDPE, Brand, Germany; 0.5 L, LDPE, Nalgene^TM^, Thermo Fisher Scientific, USA) were leached twice with HCl (*w* = 0.3%) for 1 week and 2 weeks with HNO_3_ (*w*=0.3%) and stored with HNO_3_ (*w* = 0.1%) until use. Before use, the bottles were rinsed thoroughly with ultrapure water and sample. Analytical grades HNO_3_ (65% *w*/*w*, Fisher Scientific, Germany) and HCl (30% *w*/*w*, Carl Roth, Germany) were further purified by double sub-boiling in perfluoroalkoxy-polymer (PFA)-sub-boiling stills (DST4000 & DST-1000, Savillex, USA) operated under clean room conditions. The filtrates were acidified to 0.13% (*w*/*w*) HNO_3_ and stored at 4 °C for a maximum of 6 weeks until analysis. For filtration blanks, ultrapure water (> 18.2 MΩ cm, Milli-Q Elix with Q-POD element; Merck Millipore, Germany) was used.

For nutrient analysis, the samples were filtered directly after sampling and frozen until analysis. For filtration of the nutrient samples, cellulose acetate syringe filters (0.47 µm, Minisart, Sartorius, Germany) were used on AL557 and glass fiber filters (GFF, 0.7 µm, 47 mm, Whatman^TM^, USA) on HE586.

### Instrumentation

For the determination of dissolved trace element concentrations, the filtrates were pre-concentrated with a sea*FAST* SP2 (Elemental Scientific, USA) prior to online measurement by ICP-MS/MS (Agilent 8900, Agilent Technologies, Japan). The sea*FAST* was equipped with two columns filled with Nobias chelate-PA1 resin (HITACHI High-Tech Fielding Corporation, Japan). The calibration solutions were automatically diluted from two multi-element solutions (20 ng L^−1^ and 1000 ng L^−1^) via the syringe module of the sea*FAST* SP2. The measured isotopes are listed in Table S2, for further information on the measurement routine, see Ebeling et al. (2022). The ICP-MS/MS was equipped with s-lenses and operated in single quadrupole mode with He/H_2_ (4 mL min^−1^ and 0.5 mL min^−1^, respectively) as cell gas. The instrument was tuned in a weekly routine with a tuning solution containing lithium (Li), Co, yttrium (Y), cerium (Ce), and thallium (Tl) to maintain a reliable batch-to-batch performance. Validation was based on a certified seawater reference material (NASS-7, National Research Council, Canada), as well as a multi-element standard solution (250 ng L^−1^ for Fe, 25 ng L^−1^ for all other analytes) as described by Ebeling et al. (2022). To monitor potential carryover effects, HNO_3_ (*w* = 0.07%) wash blanks were measured after each sample triplicate. Recoveries, blank concentrations, limits of detection (LOD), and limits of quantification (LOQ) are given in Table S2.

For the determination of the dissolved nutrients, standard colorimetric techniques (Hansen & Koroleff, 2007) and a continuous flow auto-analyzer (AA3, SEAL Analytical, Germany) were used. The calibration range was 0–15 µmol L^−1^ for NH_4_^+^, 0–2 µmol L^−1^ for NO_2_^−^, 0–22 µmol L^−1^ for NO_3_^−^ + NO_2_^−^, 0–3 µmol L^−1^ for PO_4_^3−^, and 0–10 µmol L^−1^ for SiO_4_^4−^. The samples were measured in duplicates, and the analysis was validated with the VKI standards QC SW3.1B and QC SW3.2B (Eurofins, Denmark; 2 µmol L^−1^ PO_4_^3−^ and NH_4_^+^, 15 µmol L^−1^ SiO_4_^4−^, 10 µmol L^−1^ NO_3_^−^ + NO_2_^−^, 1 µmol L^−1^ NO_2_^−^). LODs, LOQs, and measured concentrations of the standards are given in Table S3.

### Data evaluation

Trace element data was pre-processed using the ICP-MS/MS instrument software MassHunter version 4.6 (Agilent Technologies, Japan) operated in time-resolved mode and a custom-written Excel spreadsheet, including a Dean–Dixon outlier test for all sample triplicates. The given uncertainties of the elemental concentrations represent twice the standard deviation (SD) of the filtration replicates. The LODs and LOQs were calculated from the filtration blanks instead of analysis blanks for all elements. This is a rather conservative approach, leading to higher LODs and LOQs. Since the filtration blanks were not normally distributed, the median and the median absolute deviation (MAD) of the filtration blanks (*n* = 35) were used instead of the mean and the SD to calculate the LOD (median + 3*MAD) and LOQ (median + 10*MAD) (MacDougall et al., 1980). In case the LOD was lower than the measurement background of one analysis run, the LOD was set to this background value. The LODs and LOQs for the trace element analysis are given in Table S2. For most trace elements, all measured concentrations were above the LOQ, while Ce, europium (Eu), gallium (Ga), Gd, lanthanum (La), Mn, neodymium (Nd), praseodymium (Pr), and samarium (Sm) showed concentrations between the LOD and LOQ for some stations. Only for Fe and lead (Pb), a large share of the samples exhibited concentrations below the LOD, as we followed our previously described conservative approach (Przibilla et al., 2023) for the determination of the LOD and LOQ. However, to calculate an unbiased median, also, values below the LOD and LOQ were included.

For the nutrient concentrations, the uncertainty corresponds to the SD of the duplicate measurement. For nutrient analysis, the LOD and LOQ were calculated based on the error of the calibration line according to the German DIN 32645 (1994) for each campaign separately as the campaigns were analyzed in two different runs. The LODs and LOQs for the nutrients are given in Table S3. NO_3_^−^ and PO_4_^3−^ concentrations were mostly higher than the LOD, but 78 and 48 samples were below the LOQ. For NH_4_^+^, NO_2_^−^, and SiO_4_^4−^, almost one-third of the samples were below the LOD and another third below the LOQ.

The significant number of digits is given according to GUM and EURACHEM guidelines, whereby the uncertainty determines the significant number of digits to be presented with the value (Ellison & Williams, 2012; Magnusson et al., 2015).

For plots, the free programming language R (R Core Team, 2022) with the package tidyverse (Wickham et al., 2019) was used. A principal component analysis (PCA) with varimax rotation was calculated with the package psych (Revelle, 2024). To increase the robustness of outliers and account for the different orders of magnitude of the analytes, the data was rank-transformed and then scaled and centered prior to the PCA. For stability testing, the PCA was repeated with the upper and lower values of the measurement uncertainties of each variable.

For the PCA, the rare earth elements (REE) were summarized as light rare earth elements (LREE; Ce, Eu, Gd, La, Nd, Pr, Sm; Kulaksiz & Bau, 2007) and heavy rare earth elements (HREE; Dy, Er, Ho, Lu, Tb, Tm, Yb; Kulaksiz & Bau, 2007). The Gd anomaly (Gd/Gd*) was calculated from Nd and Sm concentrations and describes the ratio of the measured Gd concentration to the expected Gd concentration (Gd*), both normalized to the Post-Archean Australian Shale (PAAS; Kulaksiz & Bau, 2007; McLennan, 1989). From this anomaly, the percentage of anthropogenic Gd (Gd_anth_) was estimated (Kulaksiz & Bau, 2007). However, the value can be biased by anomalies in Nd and Sm and the natural Gd anomaly which is typical for seawater, which might lead to an overestimation of Gd_anth_.

## Results

In total, 337 water samples with salinities > 28.5 psu (Table S1) were analyzed for the concentration of 26 elements (cadmium (Cd), cerium (Ce), cobalt (Co), copper (Cu), dysprosium (Dy), erbium (Er), europium (Eu), iron (Fe), gadolinium (Gd), holmium (Ho), lanthanum (La), lutetium (Lu), manganese (Mn), molybdenum (Mo), neodymium (Nd), nickel (Ni), lead (Pb), praseodymium (Pr), samarium (Sm), terbium (Tb), thulium (Tm), uranium (U), vanadium (V), tungsten (W), yttrium (Y), and ytterbium (Yb)) and for the nutrients ammonium (NH_4_^+^), nitrite (NO_2_^−^), nitrate (NO_3_^−^), phosphate (PO_4_^3−^), and silicate (SiO_4_^4−^). Salinity, water depth, coordinates, and concentrations are given in Table S1 and Badewien et al. (2022). Surface concentrations and depth profiles for each analyte as well as for the sums of LREE and HREE are given in Fig. S2 to Fig. S37.

**Fig. 2:**
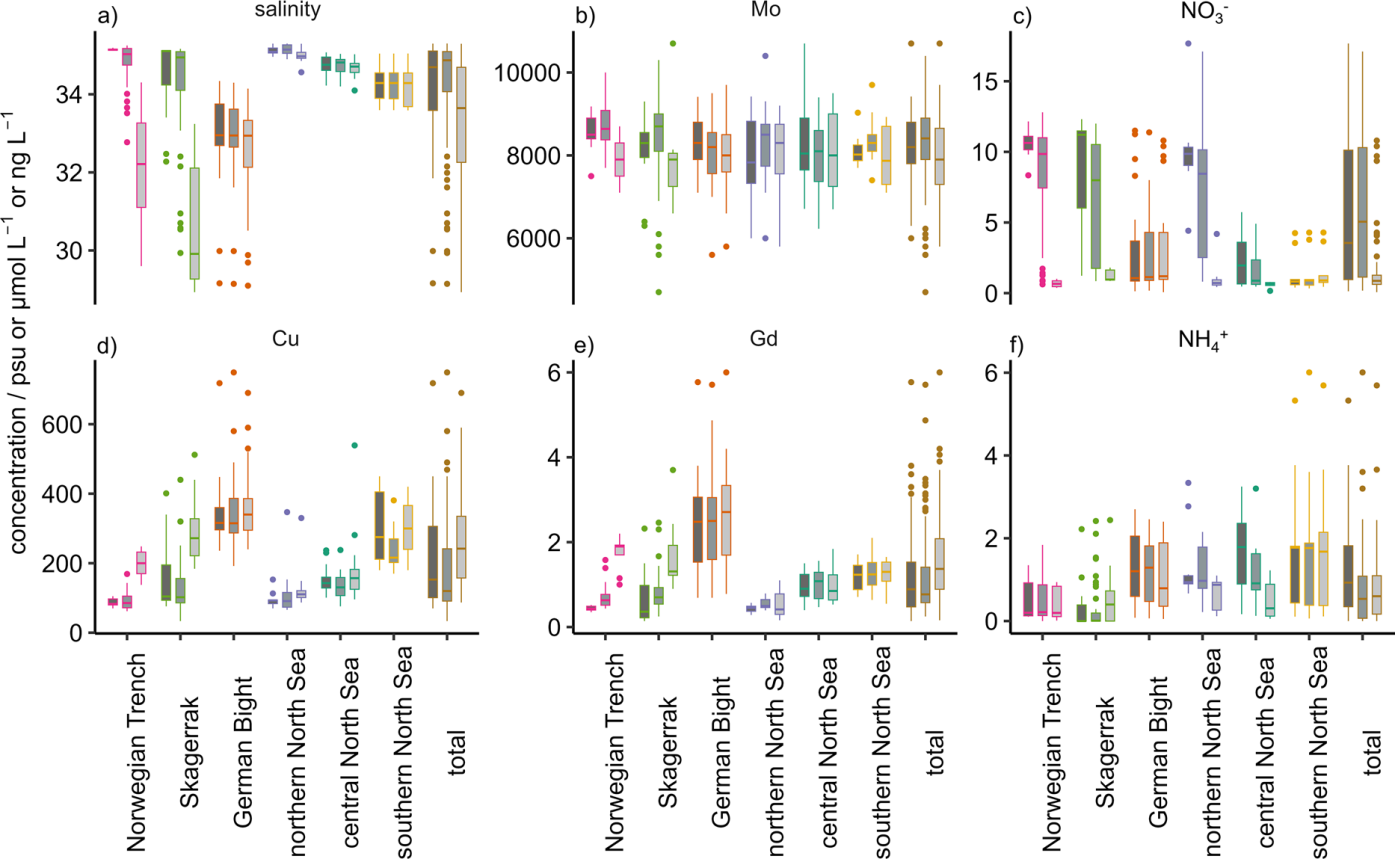
Boxplot of salinity (**a**) and concentrations of Mo (b), NO_3_^−^ (c), Cu (d), Gd (e), NH_4_^+^ (**f**) in the North Sea (total) and six regions of the North Sea that were grouped according to their location, see text for more information. The three boxplots per region indicate bottom (dark gray), intermediate depth (medium gray), and surface (light gray) samples. Boxplots of all investigated parameters are shown in Fig. S38

**Fig. 3:**
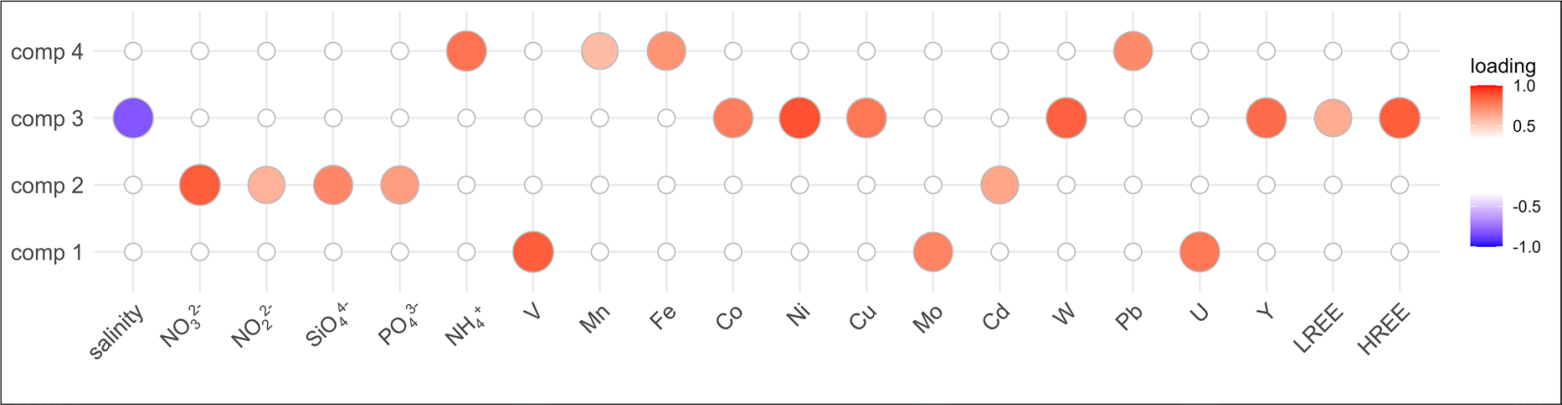
Factor loadings of the analyzed variables for the four rotated components. Note that only loadings larger than 0.5 are shown. The loadings for all analytes are given in Table S4

Due to the size of the data set, a principal component analysis (PCA) was performed to identify major trends. To give an overview of the data set, analytes that represent each component are summarized in Fig. 2 and will be described before the results of the PCA (Fig. 3) are discussed. For a better understanding of trends in the data set, the sampling stations were divided into six groups (Fig. 1) by means of their geographic location: The southern, central, and northern North Sea were subdivided along the 54° and 57° latitudes. Additionally, the regions Skagerrak, German Bight, and Norwegian Trench were approximated as they are influenced by either water mass mixing or riverine input (Topcu et al., 2011a, 2011b; Winther & Johannessen, 2006) and were hence expected to show a deviating behavior. Where applicable, the samples were further divided into the deepest (bottom), shallowest (surface), and all intermediate samples per station.

**Fig. 4:**
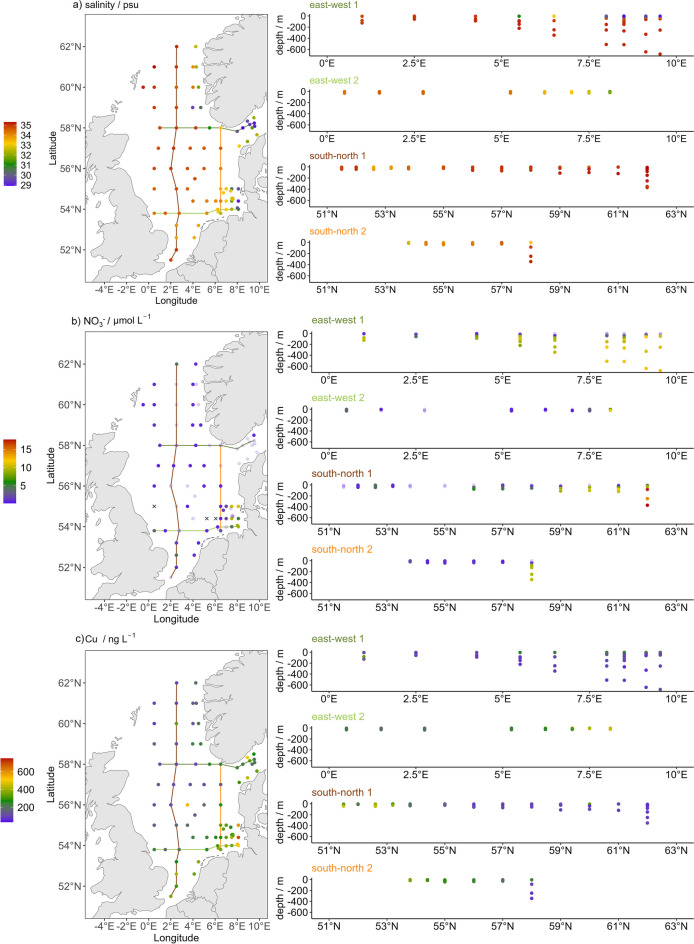
Surface concentrations and depth profiles of salinity (**a**), NO_3_^−^ (**b**), and Cu (**c**) across two south–north and two east–west transects. The northernmost transect is east–west 1 and the westernmost transect is south–north 1. x indicates that concentrations were below the LOD, faint points indicate that concentrations were between LOD and LOQ

The salinity ranged from 28.9 psu at a surface station in the Skagerrak to 35.3 psu at an intermediate depth in the northern North Sea (Figs. 2a and 4a). The northern North Sea showed the highest median salinity (35.1 psu) and the lowest variability, as illustrated by the interquartile range (IQR, 0.23 psu) while the German Bight had the lowest median salinity (33.0 psu; IQR = 1.0 psu). The Skagerrak showed the highest variability (IQR = 2.7 psu; Fig. 2a) due to the low surface salinity from the Baltic Sea inflow compared to intermediate and bottom water samples. Salinity increased with depth and to the Northwest, while especially in the German Bight, Skagerrak, and Norwegian Trench, low-salinity surface water was found (Fig. 4a).

Mo concentrations were homogeneously distributed (IQR between 700 and 1500 ng L^−1^) between all regions and showed both minimum (4700 ± 2300 ng L^−1^) and maximum (10700 ± 2500 ng L^−1^) concentrations within the Skagerrak (Fig. 2b). The highest median Mo concentrations (8500 ng L^−1^) occurred in the Norwegian Trench, and the lowest median Mo concentration was found for the central North Sea (8050 ng L^−1^, Fig. 2b).

The concentration of NO_3_^−^ ranged between < 0.17 µmol L^−1^ in the German Bight and 17.67 ± 0.23 µmol L^−1^ in the northern North Sea. NO_3_^−^ concentrations showed a high variation within all regions (IQR 3–9 µmol L^−1^), except for the southern and central North Sea (IQR 0.5 and 1.7 µmol L^−1^, respectively) which also showed lowest median NO_3_^−^ concentrations (0.9 and 0.7 µmol L^−1^, respectively; Fig. 2c). Surface concentrations of NO_3_^−^ were below 4 µmol L^−1^ throughout most of the North Sea but reached more than 10 µmol L^−1^ in the German Bight where they correlated negatively with salinity (Fig. 4b; S41). NO_3_^−^ concentrations increased with depth towards the Skagerrak and North Atlantic where the maximum concentration occurred (Fig. 4b).

Cu concentrations ranged from 33.6 ± 1.8 ng L^−1^ in the Skagerrak to 749 ± 8 ng L^−1^ in the German Bight (Fig. 2d). The median Cu concentrations ranged between 90 and 140 ng L^−1^ for the Skagerrak, Norwegian Trench, central and northern North Sea, while median concentrations were higher in the southern North Sea (260 ng L^−1^) and German Bight (320 ng L^−1^; Fig. 2d). This trend was also visible for the east–west and south–north transects (Fig. 4c): Only in the German Bight and southern North Sea, elevated Cu concentrations were found which decreased towards the deeper waters of the central and northern North Sea. Elevated Cu concentrations were found in Skagerrak surface waters close to the Norwegian coast (512 ± 5 ng L^−1^) and in the water column close to the Danish coast (up to 440 ± 50 ng L^−1^; Fig. 4c). In general, Cu concentrations correlated negatively with salinity (Fig. S41, Table S5).

Gd concentrations ranged between 0.141 ± 0.013 ng L^−1^ in the Skagerrak and 6.0 ± 2.3 ng L^−1^ in the German Bight (Fig. 2e). Median Gd concentrations ranged between 0.5 and 1.3 ng L^−1^ for most regions but reached 2.6 ng L^−1^ in the German Bight (Fig. 2e). These elevated surface concentrations decreased from the German Bight towards the Northwest and towards deeper waters (Fig. 2e; S11) and correlated negatively with salinity (Fig. S41).

NH_4_^+^ concentrations were below the LOD in most surface samples and in deep water samples from the Skagerrak, for which the median NH_4_^+^ concentration was the lowest (< 0.11 µmol L^−1^; Fig. 2f). The maximum NH_4_^+^ concentration (6.0070 ± 0.0010 µmol L^−1^) occurred within the southern North Sea which also showed the highest median NH_4_^+^ concentration (1.8 µmol L^−1^; Fig. 2f). Overall, quantifiable NH_4_^+^ concentrations were found mostly in the southern North Sea and in the coastal waters of the Skagerrak and German Bight (Fig. S19).

For the principal component analysis (PCA), only samples for which both nutrient and elemental data were available were used, and all concentrations below the LOD were set to the LOD. In total, 262 samples from 79 stations were included in the PCA. For the REE, only the sums of LREE (Ce, Eu, Gd, La, Nd, Pr, Sm) and HREE (Dy, Er, Ho, Lu, Tb, Tm, Yb) were included in the PCA to reduce their impact on the PCA. Four rotated components (RC) were chosen after varimax rotation as they explained 75% of the sample variance. Factor loadings of all input variables larger than 0.5 are shown in Fig. 3, numbers are given in Table S4. Stability testing additionally assigned Mn positively and PO_4_^3−^ negatively to component 3 while all other analytes were considered stable (Table S4).

### Component 1—Mo, U, V

Component 1 correlated positively with Mo, U, and V (0.74, 0.80, 0.86) and explained 15% of the sample variance.

U showed a similarly homogeneous distribution (1100 ± 500 to 4100 ± 500 ng L^−1^) as Mo. The lowest surface concentrations and lowest median concentrations for U were observed in the German Bight and Skagerrak (2400 ng L^−1^), and median concentrations ranged between 2800 and 3000 ng L^−1^ in all other regions with the maximum occurring at HE586 st.26 close to the Danish coast (Figs. S31, S38). V concentrations ranged from 800 ± 400 to 2060 ± 5 ng L^−1^ with the lowest median concentration (1300 ng L^−1^) and lowest surface concentrations in the German Bight and highest median concentrations in the northern North Sea (1800 ng L^−1^). For V and U, the concentrations declined towards the bottom waters of the Skagerrak (Figs. S31, S32).

### Component 2—NO_3_^−^, NO_2_^−^, SiO_4_^4−^, PO_4_^3−^, Cd

Component 2 correlated positively with the major nutrients (NO_3_^−^, NO_2_^−^, SiO_4_^4−^, PO_4_^3−^; 0.85,0.60, 0.73, 0.66) and Cd (0.65). This component explained 15% of the sample variance.

The distribution patterns of PO_4_^3−^ and SiO_4_^4−^ were comparable to NO_3_^−^ and ranged from < 0.007 to 0.977 ± 0.005 µmol L^−1^ for PO_4_^3−^ and < 0.4 to 10.50 ± 0.04 µmol L^−1^ for SiO_4_^4−^ with low surface concentrations and highest concentrations in the Skagerrak bottom waters (Fig. S24, S27). PO_4_^3−^ additionally correlated negatively with component 3 (−0.48, Table S4). NO_2_^−^ concentrations were comparably low throughout the North Sea (median 0.03 µmol L^−1^, LOD 0.03 µmol L^−1^) and reached maximum concentrations (0.760 ± 0.001 µmol L^−1^) in the deeper waters of the northern North Sea (Fig. S21). Cd showed a similar distribution as NO_3_^−^ with the lowest values at the surface of the northern North Sea (4.4 ± 0.9 ng L^−1^) and a maximum concentration (25 ± 12 ng L^−1^) at the northernmost station (Fig. S2).

### Component 3—salinity, Cu, Co, Ni, W, Y, LREE, HREE

Component 3 described 31% of the overall sample variance and correlated negatively with salinity (−0.81) and positively with Cu, Co, Ni, W, Y, LREE, and HREE (0.79, 0.76, 0.89, 0.84, 0.83, 0.72, 0.84). Co (0.67 ± 0.08 to 47 ± 4 ng L^−1^), Ni (90 ± 40 to 661 ± 4 ng L^−1^), and W (3.7 ± 1.8 to 17 ± 5 ng L^−1^) showed a similar distribution as Cu with maximum concentrations in the German Bight (Figs. S4, S20, S33). However, W also showed distinctly increased concentrations close to the Danish coast. HREE ranged between < 0.01 ng L^−1^ for Tb and 2.71 ± 0.30 ng L^−1^ for Dy (Figs. S6-S34). They showed a similar trend as Cu with highest concentrations in the German Bight (AL557 st.4; 2.45 ± 0.07, 2.199 ± 0.022, 0.638 ± 0.005, 0.45 ± 0.03, 0.330 ± 0.008, 0.35461 ± 0.00022, 2.480 ± 0.005 ng L^−1^ for Dy, Er, Ho, Lu, Tb, Tm, Yb) and in a surface sample close to the Norwegian coast (HE586, st.10; 2.71 ± 0.30, 2.05 ± 0.22, 0.63 ± 0.06, 0.32 ± 0.04, 0.42 ± 0.04, 0.296 ± 0.024, 2.06 ± 0.29 ng L^−1^ for Dy, Er, Ho, Lu, Tb, Tm, Yb). Although Y concentrations were higher throughout the analyzed samples (2.03 ± 0.05 to 29.88 ± 0.13 ng L^−1^) than for the HREE, Y is often associated with the HREE and showed similar trends with hotspots in the German Bight (29.88 ± 0.13 ng L^−1^) and the Skagerrak (28.51 ± 0.04 ng L^−1^). The LREE ranged from < 0.0097 ng L^−1^ for Eu to 16.47 ± 0.04 ng L^−1^ for La (Figs. S3-S28) with maximum concentrations for all elements at the surface of a station close to the Norwegian coast (HE586 st.10; 14.5 ± 1.6, 0.39 ± 0.05, 3.7 ± 0.6, 16.47 ± 0.04, 13.2 ± 1.1, 3.24 ± 0.30, 2.49 ± 0.24 ng L^−1^ for Ce, Eu, Gd, La, Nd, Pr, Sm). The LREE showed increased surface concentrations in the Norwegian Trench (Fig. S36).

### Component 4—Fe, Mn, Pb, NH_4_^+^

Component 4 correlated positively with Fe, Mn, Pb, and NH_4_^+^ (0.70, 0.57, 0.73, 0.80) and explained 15% of the sample variance. As for NH_4_^+^, the concentrations of Fe, Mn, and Pb were highest in the German Bight and southern North Sea.

Mn showed highest median concentrations within the southern North Sea (2025 ng L^−1^) and German Bight (2190 ng L^−1^) and significantly lower median concentrations (< 800 ng L^−1^) in all other regions (Figs. S16, S38). The maximum Mn concentration was found in the surface water of AL557 st.4 in the German Bight (12287 ± 23 ng L^−1^) and the lowest in the Norwegian Trench (55 ± 17 ng L^−1^). Mn also correlated with component 3 (0.48, Table S4). Pb values ranged from < 3.5 to 86 ± 16 ng L^−1^ with the highest median concentration (18.6 ng L^−1^) in the southern North Sea and lowest median concentrations in the Norwegian Trench (4.85 ng L^−1^) and Skagerrak (5.8 ng L^−1^; Fig. S38). Median Fe concentrations were highest in the German Bight (310 ng L^−1^) and the southern North Sea (300 ng L^−1^) and lowest in the northern North Sea (< 230 ng L^−1^, Fig. S38).

## Discussion

### Tracers for seawater and oxygen conditions

The salinity distribution was in good agreement with the dominant currents in the North Sea (Figs 1 and 4a; Brückner and Mackensen, 2006). The median salinities decreased from the northern to the southern North Sea with decreasing impact of North Atlantic water and increasing freshwater impact (Fig. 2a). In the Skagerrak, the surface waters had low salinities due to mixing with the Baltic Sea water, while the deeper waters featured higher salinities due to the inflow of Atlantic and central North Sea deep water (Fig. 4a). The low salinity in the German Bight (Fig. 4a) was caused by riverine discharges.

Mo, U, and V (component 1) concentrations are high in oxygenated seawater but decrease during mixing with fresh water in the North Sea (Schneider et al., 2016; Tribovillard et al., 2006). This was illustrated by the lowest median concentrations of U and V in the German Bight and Skagerrak where seawater mixes with lower-salinity water originating from rivers and the Baltic Sea. However, for Mo, the trend was not as clear as for U and V and component 1 only correlated weakly with salinity (0.43, Table S4). Hence, also other processes than conservative mixing from the Atlantic Ocean, Baltic Sea, and rivers played a role. Mo concentrations in the German Bight were in the same range as previously reported by Dellwig et al. (2007) who showed deviations from conservative mixing for Mo that is involved in biogeochemical processes (Dellwig et al., 2007; Kowalski et al., 2013). This is in good agreement with the increased Mo concentrations from HE586 (October) samples compared to AL557 (June) samples (Fig. S41), indicating the release of Mo that was adsorbed to organic matter during summer. Mo and V may also have anthropogenic sources and reach the ocean by atmospheric deposition (Schlesinger et al., 2017; Schneider et al., 2016; Wong et al., 2021), but we did not find clear evidence for this in our data.

The bottom waters of the Skagerrak are only renewed every 2 to 3 years while oxygen is continuously consumed by the degradation of organic matter (Brückner & Mackensen, 2006; Johannessen & Dahl, 1996). This likely leads to a depletion of U and V in Skagerrak bottom waters under suboxic conditions (Figs. S31, S32). Although Mo and V are involved in microbial processes of the nitrogen cycle and PP (Butler, 1998; Meisch & Bielig, 1975; Morel & Price, 2003; Rehder, 2015) and correlate with PP in the open Atlantic Ocean (Pinedo-González et al., 2015), they are sufficiently high concentrated in oxic sea water to not be limiting for phytoplankton growth (Rehder, 2015). However, due to climate warming and anthropogenic inputs, oxygen minimum zones and regions with anoxic sediments might expand in the future, removing dissolved Mo, U, and V from the water column (Emerson & Huested, 1991). Hence, an impact on phytoplankton growth might be observed in the future.

### Nutrients and nutrient-like analytes

The measured nutrient concentrations were in the same range as reported for the North Sea by previous studies (Andersson, 1996; Chaichana et al., 2019; Wiltshire et al., 2010). The increasing concentrations of NO_3_^−^, PO_4_^3−^, and SiO_4_^4−^ with increasing water depth and towards the North Atlantic (Fig. 4b, S24, S27) reflect the remineralization of organic matter in the deeper waters of the open North Sea and the export of nutrients into the North Atlantic (Thomas et al., 2004). The nutrient concentrations in the German Bight were in the same range as reported for summer and autumn 2011 (BSH, 2016) and in the Skagerrak comparable to those reported for July 1997 for the upper 50 m (Croot et al., 2002). In the Skagerrak, the nutrient concentrations were lowest in surface waters with higher fractions of low-salinity Baltic Sea water (Croot et al., 2002). The cruise in June (AL557) likely took place after an algae bloom, while for the autumn cruise (HE586), the increased PO_4_^3−^ and SiO_4_^4−^ concentrations in the German indicate nutrient recycling (Fig. S41). This was not evident for NO_3_^−^ (Fig. S41), likely due to high riverine inputs during summer and the high variability of biogeochemical processes in this region. Nevertheless, the data was in good agreement with monitoring data from the German Bight which indicates high seasonal and year-to-year variability of nutrients (BSH, 2016). In the German Bight, nutrients are mainly supplied by rivers, whereas deeper waters of the North Sea are influenced by recycling of organic matter and mixing with high-nutrient Atlantic Ocean water (Chaichana et al., 2019; Jickells, 1998; Topcu et al., 2011a, 2011b). In the central and southern North Sea, Atlantic water inflow with lower N-to-P ratios is more important than terrestrial input (Burson et al., 2016) and recycled nutrients are directly consumed, leading to low nutrient concentrations. In addition to riverine input, atmospheric deposition can act as another source of nutrients. Atmospheric nitrogen inputs to the North Sea were estimated to be as high as 36% of riverine input and are mainly deposited in the southern North Sea and German Bight (de Leeuw et al., 2003; Pätsch & Kühn, 2008). Phosphorus and silicon are mainly supplied with dust (Jickells et al., 2016; Mahowald et al., 2008). However, since most samples in the southern North Sea and German Bight were taken in summer, possible atmospheric inputs of nutrients were likely quickly consumed.

**Fig. 5:**
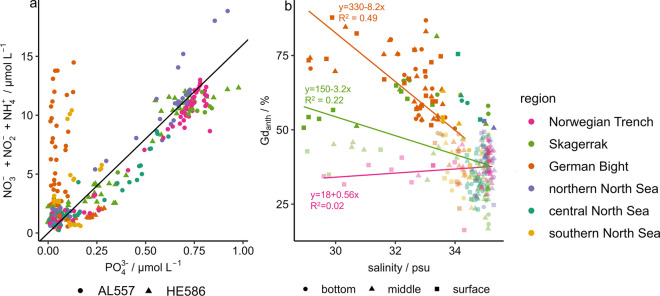
Correlation of inorganic nitrogen with PO_4_^3−^ for AL557 (circle) and HE586 (triangle) (**a**) and percentage of Gd_anth_ versus salinity for the bottom (circle), middle (triangle), and surface (square) samples (**b**). The diagonal line (**a**) indicates the N-to-P ratio of 16 (*n*/*n*). Pale points indicate the samples with Gd anomalies < 2 (**b**)

For PP, the ratio of dissolved inorganic nitrogen species (DIN; NO_2_^−^, NO_3_^−^, NH_4_^+^) and PO_4_^3−^ should be close to 16:1 (*n*/*n*; Redfield et al., 1963) and is typically found in seawater where biomass recycling is followed by nutrient uptake. Most samples from the Skagerrak, Norwegian Trench, northern and central North Sea followed this ratio (median 15–17 (*n*/*n*); Fig. 5a). However, the samples from the German Bight and southern North Sea deviated from this distribution with a high median N-to-P ratio of 50 (*n*/n; IQR = 130) for the German Bight and 30 (*n*/*n*; IQR = 60) for the southern North Sea. This was due to high NO_3_^−^ concentrations during June (up to 11.5 ± 0.5 µmol L^−1^), indicating a significant surplus of NO_3_^−^ compared to PO_4_^3−^. Comparable N-to-P ratios were found for the major tributaries of the North Sea (van Beusekom et al., 2019) and for the bottom waters of the coastal German Bight (> 50 (*n*/*n*)) during the winter seasons of 2008–2011 but with high year-to-year variability (BSH, 2016). NO_3_^−^ concentrations of the German Bight correlated negatively with salinity during June (*R*^2^ = 0.8), indicating a strong NO_3_^−^ input from terrestrial sources (e.g., agriculture) with distinct seasonal variability (Pätsch et al., 2004). In contrast, in October (HE586), the NO_3_^−^ concentrations were lower, and the PO_4_^3−^ concentrations were slightly higher in the German Bight compared to June (Fig. 5a). This indicates less anthropogenic nitrogen input and more nutrient recycling during autumn. Although most of our samples were taken in June, this shows the increasing P-limitation of the German Bight and southern North Sea that is caused by the regulation of riverine P input while nitrogen inputs remained high during the past decades (Balkoni et al., 2023; Burson et al., 2016; Pätsch et al., 2004).

NO_2_^−^ is an intermediate product of nitrification and denitrification but is easily oxidized to NO_3_^−^ under oxic conditions and reduced under anoxic conditions (Zhang et al., 2020). Therefore, its concentration was mostly below the LOD in this study. NH_4_^+^ is also produced during organic matter remineralization and quickly oxidized (Zhang et al., 2020). Hence, local hotspots for both analytes might occur during the remineralization of organic matter. The PCA attributed NH_4_^+^ to component 4, indicating that NH_4_^+^ was not only influenced by organic matter remineralization but also by redox processes or atmospheric deposition (de Leeuw et al., 2003). The increased NH_4_^+^ concentrations in the coastal waters of the Skagerrak, German Bight, and southern North Sea were probably caused by remineralization of decaying phytoplankton blooms (Chaichana et al., 2019; Johnson et al., 2007). The highest median NH_4_^+^ concentrations (1.8 µmol L^−1^, IQR 1.6 µmol L^−1^) were found in the southern North Sea where it significantly contributed to high surface N-to-P ratios. This might have been caused by the decoupling of remineralization and PP or atmospheric deposition (Chaichana et al., 2019; de Leeuw et al., 2003).

In addition to the traditional nutrients, Cd correlated positively with component 2. Cd is needed by diatoms for CO_2_ sequestration (Lane et al., 2005) and has a nutrient-like depth profile in the North Atlantic (Kremling et al., 1999; Pinedo-González et al., 2015). Higher Cd concentrations in the deeper waters of the northern North Sea might therefore be due to the recycling of organic matter that contained or adsorbed Cd. The maximum Cd concentrations reported in this study are lower than the maximum of 51 ng L^−1^ Cd reported for the open North Sea by Laslett (1995). Over the past decades, riverine and atmospheric inputs were the main Cd sources (Laane et al., 2013; Pacyna et al., 2009), but it seems that anthropogenic Cd contamination of the coastal regions decreased drastically, making the Atlantic Ocean a source for Cd to the North Sea.

Various studies revealed contrasting effects of eutrophication and climate change on the North Sea: Especially for the beginning of the spring bloom, the winter reserve from internal nutrient recycling and light availability is more important in the North Sea than riverine input (Frigstad et al., 2020; Leote et al., 2016; McQuatters-Gollop et al., 2007; Wiltshire et al., 2015). However, riverine input is important for the duration of the bloom and phytoplankton growth, especially in coastal regions (Frigstad et al., 2020; Gross et al., 2022; Skogen et al., 2004). Limitations of specific nutrients can cause a shift in the phytoplankton community, due to the different demands of the species (Gross et al., 2022; Grosse et al., 2017; Gypens et al., 2017). Hence, unbalanced nutrient inputs can support toxic algae blooms (Emeis et al., 2015; Lefebvre & Dezecache, 2020). Especially in coastal regions, diatom growth can also be limited by SiO_4_^4−^ availability (Burson et al., 2016; Lefebvre & Dezecache, 2020).

As climate change might reduce the vertical water mass mixing in the Atlantic Ocean, nutrient concentrations of the water entering the North Sea from the North Atlantic might decrease (Gröger et al., 2013). In case riverine inputs remain P-limited, this might lead to further changes in the phytoplankton community within the North Sea.

### Tracers of riverine input

Co, Cu, Ni, and W were summarized by component 3 which correlated negatively with salinity (Fig. 3; S4-S35, S41). The main source for these elements is riverine input, and they are diluted with increasing distance to the river mouths in the German Bight and southern North Sea (BSH, 2016; Hydes & Kremling, 1993; Kremling et al., 1999; Kulaksiz & Bau, 2007; Mart et al., 1985; Sohrin et al., 1987). In the open ocean, these elements are prone to scavenging or biological uptake (Kremling et al., 1999; Sohrin et al., 1987). However, due to the scattering of the sampling points in our study, only conservative mixing with North Sea water was observed (Fig. S41). Only for Co in the German Bight, a positive correlation with salinity was observed (Fig. S41). The higher concentrations of these analytes in the Skagerrak surface waters originated from Baltic Sea water (Croot et al., 2002; Kremling & Petersen, 1978), as well as transport of German Bight water to the Skagerrak via the southern Jutland current (Fig. 1). During the winters of 2008–2011, maximum Cu and Ni concentrations in the Elbe river mouth were 800 ng L^−1^ and decreased with increasing distance within the German Bight, which is in good agreement with our findings (BSH, 2016). Co and Cu concentrations in the Skagerrak were in the same order of magnitude as for a transect measured in July 1997, with the highest Co concentrations at the coastal station (Croot et al., 2002). Co, Cu, Fe, Mn, and Ni are required for the nitrogen cycle and other processes within marine phytoplankton (Morel & Price, 2003; Twining & Baines, 2013). Since the elemental requirements vary between species, Co, Cu, and Mn availability can affect the phytoplankton community structure (Sunda, 2012). However, increased Cu and Ni concentrations might also be toxic or increase P-limitation by complexation (Thomas et al., 1980; Yang et al., 2019). Dissolved Cu might inhibit growth at concentrations above 65 ng L^−1^ for some species while most species have a significantly higher tolerance and are mostly harmed by Cu^2+^ which usually features low concentrations in seawater (Yang et al., 2019). Nevertheless, this indicates that anthropogenic Cu inputs need to be limited, especially since it is also supplied by atmospheric deposition (Paytan et al., 2009). To cover the year-to-year variability of these elements, as shown for the German Bight (BSH, 2016), further studies are required to monitor trends in anthropogenic inputs.

The REE and Y are mainly supplied by rivers and behave inversely to salinity, but LREE might also adsorb to organic matter and be released upon remineralization (Goldstein & Jacobsen, 1988; Sutorius et al., 2022). In our study, this is reflected by the high median concentrations of all REE in the German Bight and the high concentrations of the REE in Norwegian Trench surface waters compared to deeper water samples (Fig. S38). The REE concentrations at AL557 st.2 and st.8, closest to the Elbe and Weser rivers were comparable to those described by Kulaksiz & Bau (2007) a few km south at similar salinities. The high REE concentrations in the surface waters of the Norwegian Trench were likely due to freshwater inputs with low anthropogenic impact, e.g., from fjords.

For most REE, the median concentrations were highest in the German Bight. However, for Gd, a large discrepancy between the German Bight and all other regions was found (Fig. 2e, Fig. S38). REE and Y share a very similar biogeochemistry, resulting in consistent ratios of these elements throughout marine and terrestrial environments. Deviations of certain elements from this behavior (anomalies), e.g., by anthropogenic impact or distinct chemical processes can be revealed by normalization to a reference material (e.g., Post-Archean Australian Shale (PAAS); Kulaksiz & Bau, 2007). A natural Gd anomaly of 1.6 (Gd/Gd*) was calculated for the North Atlantic but is exceeded by anthropogenic inputs in coastal regions (Kulaksiz & Bau, 2007). Within the aquatic environment, magnetic resonance imaging contrast agents are the main sources of anthropogenic Gd (Kulaksiz & Bau, 2007). As the contained Gd complexes have a high solubility and stability, they are not removed by wastewater treatment plants but reach the oceans via rivers, making Gd a tracer for anthropogenic riverine input (Kulaksiz & Bau, 2007). From the Gd anomaly, Gd_anth_ (percentage of anthropogenic Gd) was calculated, as shown in Fig. 5b and Fig. S12. To account for the natural seawater Gd anomaly and the uncertainties associated with the calculation of Gd_anth_, only samples with anomalies > 2 (~50% Gd_anth_) will be discussed for anthropogenic input. We calculated that up to 88% of the Gd in our samples was of anthropogenic origin and in general, a decrease of Gd_anth_ with increasing salinity was evident for the Skagerrak and German Bight (Fig. 5b; S12). For 2005, Kulaksiz and Bau (2007), reported anomalies of 5–7 for the Weser river (~85% Gd_anth_, 3–7 psu), decreasing within the estuary (anomaly of 4, ~75% Gd_anth_, 14–20 psu) towards an anomaly of 1.6–2 (~40–50% Gd_anth_, 32 psu) off the East Frisian Islands. In our study, the rivers and estuaries were not covered, but the highest Gd_anth_ values were found in the surface waters of the German Bight and decreased to the Northwest. The maximum values of 82 ± 8% and 70 ± 4% Gd_anth_ at the southernmost stations in the German Bight (AL557 st.2 and st.8) were significantly higher than those estimated by Kulaksiz and Bau (2007) for similar locations and salinities nearly two decades ago. This indicates that the high Gd_anth_ that was reported for the Weser estuary by Kulaksiz and Bau (2007) is propagating into the German Bight from the main river estuaries. This reflects the doubling of MRI exams in Germany between 2007 and 2021 (OECD, n.d). However, more samples are needed as the applied calculation results in high uncertainties (Table S1). Elevated Gd_anth_ values were also calculated for the Danish coast (up to 80%, Fig. S12). These likely originated from the German Bight, as a surface water drift model^1^ (Callies et al., 2021) indicated the northward transport of these water masses via the southern Jutland current (Fig. S39). The low fractions of Gd_anth_ in the surface waters of the Norwegian Trench support the hypothesis that the elevated REE concentrations originated from freshwater inputs with low anthropogenic disturbance (Fig. S12). There are no studies available on the effects of Gd on phytoplankton but studies with higher marine organisms suggest negative effects of Gd on marine life (Trapasso et al., 2021).

Two stations showed noticeably elevated elemental concentrations: One surface water sample close to the Norwegian coast off Arendal (HE586, st.10) with significantly higher REE concentrations and all samples of AL557 st.4 in the German Bight with higher concentrations of Cd, Cu, Fe, Mn, Ni, W, and all HREE, while LREE remained low (Figs. S2-S35). Sediment resuspension or submarine groundwater input was unlikely for both stations as the maximum concentrations occurred at the surface. For the surface sample near Arendal, the salinity was lower (30 psu) than the deeper and surrounding samples (33–35 psu), indicating that this water mass might have originated from the Kattegat (Aure et al., 1998; Kristiansen & Aas, 2015). This is supported by a surface drift model^1^ (Callies et al., 2021) showing that the water mass originated from the eastern Skagerrak during HE586, while the currents were different during AL557 (Fig. S40). In contrast, for AL557 st.4, the surface drift model^1^ (Fig. S39; Callies et al., 2021) showed that the water mass has been moving in the region due to tidal activity but might have originated from the Eider or Elbe estuary. This would be supported by the low salinity of this sample (Fig. 4a).

Fe, Mn, and Pb are also supplied mainly from rivers and are therefore discussed in this section (BSH, 2016; Laane et al., 2013; Ussher et al., 2007). However, although Mn was also assigned to component 3 during stability testing, Fe, Mn, and Pb were best represented by component 4 (Fig. 3, Table S4). This was likely caused by atmospheric deposition (e.g., from dust or anthropogenic sources) and scavenging by organic matter or other particles that are known for these elements (Jickells et al., 2016; Kremling et al., 1999; Pacyna et al., 2009; Ussher et al., 2007). Pb concentrations were highest in the southern and central North Sea and in the German Bight, likely due to riverine input and atmospheric deposition (Pacyna et al., 2009), but sediment resuspension might also have played a role (Tappin et al., 1995). This patchiness is in good agreement with monitoring results for the winters of 2008-2011 in the German Bight (BSH, 2016). However, except for one water sample from AL557 st.53 (86 ± 16 ng L^−1^), our Pb concentrations were lower than the reported hotspot concentrations (~50–100 ng L^−1^; BSH, 2016), likely due to scavenging of Pb on fresh organic matter during summer. In general, the Pb concentrations found for the North Sea were significantly lower than toxic concentrations for Atlantic Ocean phytoplankton (20 µg L^−1^; Echeveste et al., 2012). As a high year-to-year variability was observed for Pb before (BSH, 2016), further studies are needed to monitor the development of Pb concentrations in the North Sea.

Dissolved Fe and Mn are quickly oxidized and precipitated when they are introduced to oxygenated seawater. Hence, their concentrations correlated well with salinity in the German Bight with high river discharge. Except for AL557 st.4 and st.9, the Mn concentrations in the German Bight were in the same range as those found for the winters 2008–2011, decreasing from the river mouths to the open North Sea (BSH, 2016). In the Skagerrak, the trend was not as clear because low-salinity Baltic water, from which Fe and Mn might have already precipitated, mixes with high-salinity water. Mn and Fe are important for phytoplankton growth and Fe often acts as a limiting micronutrient (Sunda, 2012; Twining & Baines, 2013). However, due to high blank concentrations, no significant spatial or seasonal differences were found for Fe in this study. Mn concentrations are most likely not limiting in the North Sea, as concentrations were mostly higher than in the open ocean due to the terrestrial input (Sunda, 2012). However, Mn availability might impact the phytoplankton community structure (Sunda, 2012). With increasing sea surface temperature, metal oxidation will increase, while decreasing seawater pH will increase metal reduction (Hoffmann et al., 2012) and thereby might change the availability of these redox-active scavenged elements.

## Conclusion

Our study gives a comprehensive overview of dissolved trace element and nutrient concentrations for the entire North Sea with a high sampling resolution. Although biogeochemical processes are highly variable and underlay interannual and seasonal changes, the results are in the same range as previous studies. This data set will be useful for ocean models and to better understand inorganic influences on PP, carbon uptake, and carbon storage within the North Sea. The presented data set illustrates riverine impact most clearly for the German Bight. However, riverine impact is also evident for the Southern Bight even though the Belgian, British, and Dutch coastal areas were not covered by the sampling strategy. To also monitor seasonal and year-to-year differences, further large datasets are needed in the future.

The PCA confirmed that metal and nutrient concentrations in the North Sea are mainly dependent on river run-off, water mass mixing, and recycling of organic matter but also atmospheric deposition might play a role. Therefore, anthropogenic inputs and their regulation remain important. In further studies, species-specific analysis of selected elements like Fe and Cu would be a valuable tool to determine the bioavailability of the analyzed elements. However, in this study, we focused on a first comprehensive data set which is important to outline those areas that are most important for further investigations related to carbon cycling and long-term storage.

### Supplementary Information

Below is the link to the electronic supplementary material.

Supplementary file1 (XLSX 164 KB)

Supplementary file2 (DOCX 10823 KB)

## Data Availability

All data is provided in the supplementary information. The data is also accessible on PANGEA (https://doi.pangaea.de/10.1594/PANGAEA.967369; https://doi.pangaea.de/10.1594/PANGAEA.967370).

## Footnotes

1 hcdc.hereon.de/drift-now/

## References

[CR1] Andersson L (1996). Trends in nutrient and oxygen concentrations in the Skagerrak-Kattegat. Journal of Sea Research.

[CR2] Aure J, Danielssen D, Svendsen E (1998). The origin of Skagerrak coastal water off Arendal in relation to variations in nutrient concentrations. Ices Journal Of Marine Science.

[CR3] Badewien, T. H., Hoppmann, M., Tippenhauer, S. (2022). *Physical oceanography during RV HEINCKE cruise HE586* PANGAEA. 10.1594/PANGAEA.940830

[CR4] Balkoni A, Guignard MS, Boersma M, Wiltshire KH (2023). Evaluation of different averaging methods for calculation of ratios in nutrient data. Fundamental and Applied Limnology.

[CR5] Bozec Y, Thomas H, Elkalay K, de Baar HJW (2005). The continental shelf pump for CO2 in the North Sea—Evidence from summer observation. Marine Chemistry.

[CR6] Brückner S, Mackensen A (2006). Deep-water renewal in the Skagerrak during the last 1200 years triggered by the North Atlantic oscillation: Evidence from benthic foraminiferal [delta] 18O. The Holocene.

[CR7] BSH (2016). Nordseezustand 2008-2011, Berichte des BSH, Nr. 54, 311 pp., Bundesamt für Seeschifffahrt und Hydrographie. https://www.bsh.de/download/Berichte-des-BSH-54.pdf

[CR8] Burson A, Stomp M, Akil L, Brussaard CPD, Huisman J (2016). Unbalanced reduction of nutrient loads has created an offshore gradient from phosphorus to nitrogen limitation in the North Sea. Limnology and Oceanography.

[CR9] Butler A (1998). Acquisition and utilization of transition metal ions by marine organisms. Science.

[CR10] Cai W-J, Dai M (2004). Comment on “Enhanced open ocean storage of CO2 from shelf sea pumping”. Science.

[CR11] Callies U, Kreus M, Petersen W, Voynova YG (2021). On using lagrangian drift simulations to aid interpretation of in situ monitoring data. Frontiers in Marine Science.

[CR12] Chaichana S, Jickells T, Johnson M (2019). Interannual variability in the summer dissolved organic matter inventory of the North Sea: Implications for the continental shelf pump. Biogeosciences.

[CR13] Chavez FP, Messié M, Pennington JT (2011). Marine primary production in relation to climate variability and change. Annual Review of Marine Science.

[CR14] Croot PL, Karlson B, Wulff A, Linares F, Andersson K (2002). Trace metal/phytoplankton interactions in the Skagerrak. Journal of Marine Systems.

[CR15] de Leeuw, G., Skjøth, C. A., Hertel, O., Jickells, T., Spokes, L., Vignati, E, ... , Kunz, G. J. (2003). Deposition of nitrogen into the North Sea. *Atmospheric environment*, *37*, 145-165. 10.1016/S1352-2310(03)00246-2

[CR16] Dellwig O, Beck M, Lemke A, Lunau M, Kolditz K, Schnetger B, Brumsack HJ (2007). Non-conservative behaviour of molybdenum in coastal waters: Coupling geochemical, biological, and sedimentological processes. Geochimica et Cosmochimica Acta.

[CR17] DIN 32645. (1994). *Chemical analysis; Decision limit; Detection limit and determination limit; Estimation in case of repeatability; Terms, methods, evaluation*. https://www.beuth.de/en/standard/din-32645/2290434

[CR18] Ebeling A, Zimmermann T, Klein O, Irrgeher J, Pröfrock D (2022). Analysis of seventeen certified water reference materials for trace and technology-critical elements. Geostandards and Geoanalytical Research.

[CR19] Echeveste, P., Agustí, S., Tovar-Sánchez, A. (2012). Toxic thresholds of cadmium and lead to oceanic phytoplankton: Cell size and ocean basin–dependent effects. *Environmental Toxicology and Chemistry*, *31*(8), 1887-1894. 10.1002/etc.1893 10.1002/etc.1893 22619131

[CR20] Ellison, S. L. R., & Williams, A. (2012). *Eurachem/CITAC guide: Quantifying uncertainty in analytical measurement* (3rd ed). https://www.eurachem.org/images/stories/Guides/pdf/QUAM2012_P1.pdf

[CR21] Emeis K-C, van Beusekom J, Callies U, Ebinghaus R, Kannen A, Kraus G, Kröncke I, Lenhart H, Lorkowski I, Matthias V, Möllmann C, Pätsch J, Scharfe M, Thomas H, Weisse R, Zorita E (2015). The North Sea — A shelf sea in the Anthropocene. Journal of Marine Systems.

[CR22] Emerson SR, Huested SS (1991). Ocean anoxia and the concentrations of molybdenum and vanadium in seawater. Marine chemistry.

[CR23] Field CB, Behrenfeld MJ, Randerson JT, Falkowski P (1998). Primary production of the biosphere: Integrating terrestrial and oceanic components. Science.

[CR24] Fock HO (2003). Changes in the seasonal cycles of inorganic nutrients in the coastal zone of the southeastern North Sea from 1960 to 1997: Effects of eutrophication and sensitivity to meteoclimatic factors. Marine pollution bulletin.

[CR25] Frigstad H, Andersen T, Hessen DO, Jeansson E, Skogen M, Naustvoll LJ, Miles MW, Johannessen T, Bellerby RGJ (2013). Long-term trends in carbon, nutrients and stoichiometry in Norwegian coastal waters: Evidence of a regime shift. Progress in oceanography.

[CR26] Frigstad, H., Kaste, O., Deininger, A., Kvalsund, K., Christensen, G., Bellerby, R. G. J., Sorensen, K., Norli, M., King, A. L. (2020). Influence of riverine input on Norwegian coastal systems. *Frontiers in marine science*,* 7*. 10.3389/fmars.2020.00332

[CR27] Gao Y, Brauwere A, Elskens M, Croes K, Baeyens W, Leermakers M (2013). Evolution of trace metal and organic pollutant concentrations in the Scheldt River basin and the Belgian coastal zone over the last three decades. Journal of Marine Systems.

[CR28] Goldstein SJ, Jacobsen SB (1988). Rare earth elements in river waters. Earth and planetary science letters.

[CR29] Gröger M, Maier-Reimer E, Mikolajewicz U, Moll A, Sein D (2013). NW European shelf under climate warming: Implications for open ocean-shelf exchange, primary production, and carbon absorption. Biogeosciences.

[CR30] Gross E, Di Pane J, Boersma M, Meunier CL (2022). River discharge-related nutrient effects on North Sea coastal and offshore phytoplankton communities. Journal of Plankton Research.

[CR31] Grosse J, van Breugel P, Brussaard CPD, Boschker HTS (2017). A biosynthesis view on nutrient stress in coastal phytoplankton. Limnology and oceanography.

[CR32] Gypens N, Borges AV, Ghyoot C (2017). How phosphorus limitation can control climate-active gas sources and sinks. Journal of Marine Systems.

[CR33] Hansen, H. P., & Koroleff, F (2007) Determination of nutrients. Methods of seawater analysis Third, completely revised and extended edition. Weinheim, Germany: Wiley-VCH Verlag GmbH

[CR34] Hickel W, Mangelsdorf P, Berg J (1993). The human impact in the German Bight: Eutrophication during 3 decades (1962–1991). Helgolander Meeresuntersuchungen.

[CR35] Hoffmann LJ, Breitbarth E, Boyd PW, Hunter KA (2012). Influence of ocean warming and acidification on trace metal biogeochemistry. Marine ecology progress series.

[CR36] Hydes DJ, Kremling K (1993). Patchiness in dissolved metals (Al, Cd Co, Cu, Mn, Ni) in North Sea surface waters: Seasonal differences and influence of suspended sediment. Continental shelf research.

[CR37] Ito, S., Rose, K. A., Miller, A. J., Drinkwater, K., Brander, K., Overland, J. E., Sundby, S., Curchitser, E., Hurrell, J.W., Yamanaka, Y. (2010). 287Chapter 10 ocean ecosystem responses to future global change scenarios: A way forward. In: *Marine ecosystems and global change*. Oxford University Press. 10.1093/acprof:oso/9780199558025.003.0010

[CR38] Jickells TD (1998). Nutrient biogeochemistry of the coastal zone. Science.

[CR39] Jickells TD, Baker AR, Chance R (2016). Atmospheric transport of trace elements and nutrients to the oceans. Philosophical transactions of the royal society A: Mathematical, physical and engineering sciences.

[CR40] Johannessen T, Dahl E (1996). Declines in oxygen concentrations along the Norwegian Skagerrak coast, 1927–1993: A signal of ecosystem changes due to eutrophication?. Limnology and oceanography.

[CR41] Johnson M, Sanders R, Avgoustidi V, Lucas M, Brown L, Hansell D, Moore M, Gibb S, Liss P, Jickells T (2007). Ammonium accumulation during a silicate-limited diatom bloom indicates the potential for ammonia emission events. Marine chemistry.

[CR42] Kowalski N, Dellwig O, Beck M, Grawe U, Neubert N, Nagler TF, Badewien TH, Brumsack HJ, van Beusekom JEE, Bottcher ME (2013). Pelagic molybdenum concentration anomalies and the impact of sediment resuspension on the molybdenum budget in two tidal systems of the North Sea. Geochimica et Cosmochimica Acta.

[CR43] Kremling, K., Andreae, M. O., Brügmann, L., Van den Berg, C. M. G., Prange, A., Schirmacher, M., Koroleff, E., Kus, J. (1999). Determination of trace elements. *Methods of seawater analysis*, 253-364. 10.1002/9783527613984.ch12

[CR44] Kremling K, Petersen H (1978). The distribution of Mn, Fe, Zn, Cd and Cu in Baltic seawater; a study on the basis of one anchor station. Marine chemistry.

[CR45] Kristiansen T, Aas E (2015). Water type quantification in the Skagerrak, the Kattegat and off the Jutland west coast. OCEANOLOGIA.

[CR46] Kulaksiz S, Bau M (2007). Contrasting behaviour of anthropogenic gadolinium and natural rare earth elements in estuaries and the gadolinium input into the North Sea. Earth and planetary science letters.

[CR47] Laane R, Vethaak AD, Gandrass J, Vorkamp K, Kohler A, Larsen MM, Strand J (2013). Chemical contaminants in the Wadden Sea: Sources, transport, fate and effects. Journal of Sea Research.

[CR48] Lane TW, Saito MA, George GN, Pickering IJ, Prince RC, Morel FMM (2005). A cadmium enzyme from a marine diatom. Nature.

[CR49] Laslett RE (1995). Concentrations of dissolved and suspended particulate Cd, Cu, Mn, Ni, Pb and Zn in surface waters around the coasts of England and Wales and in adjacent seas. Estuarine coastal and shelf science.

[CR50] Lefebvre, A., Dezecache, C. (2020). Trajectories of changes in phytoplankton biomass, Phaeocystis globosa and diatom (incl. Pseudo-nitzschia sp.) abundances related to nutrient pressures in the eastern English Channel, southern North Sea. *Journal of Marine Science and Engineering*,* 8*(6). 10.3390/jmse8060401

[CR51] Lenhart HJ, Pätsch J, Kühn W, Moll A, Pohlmann T (2004). Investigation on the trophic state of the North Sea for three years (1994? 1996) simulated with the ecosystem model ERSEM? The role of a sharp NAOI decline. Biogeosciences discussions.

[CR52] Leote C, Mulder LL, Philippart CJM, Epping EHG (2016). Nutrients in the western Wadden Sea: Freshwater input versus internal recycling. Estuaries and coasts.

[CR53] MacDougall D, Crummett WB (1980). Guidelines for data acquisition and data quality evaluation in environmental chemistry. Analytical chemistry.

[CR54] Macovei VA, Voynova YG, Becker M, Triest J, Petersen W (2021). Long-term intercomparison of two p CO2 instruments based on ship-of-opportunity measurements in a dynamic shelf sea environment. Limnology and oceanography: Methods.

[CR55] Magnusson, B., Ellison, S. L. R., & Örnemark, U. (2015). *Eurachem guide: Template for Eurachem guides – A guide for guide editors*. http://www.eurachem.org

[CR56] Mahowald, N., Jickells, T. D., Baker, A. R., Artaxo, P., Benitez-Nelson, C. R., Bergametti, G., ... , Tsukuda, S. (2008). Global distribution of atmospheric phosphorus sources, concentrations and deposition rates, and anthropogenic impacts. *Global biogeochemical cycles*, *22*(4). 10.1029/2008GB003240

[CR57] Mart L, Nürnberg HW, Rützel H (1985). Levels of heavy metals in the tidal Elbe and its estuary and the heavy metal input into the sea. Science of the total environment.

[CR58] McLennan, S. M. (1989). Geochemistry and mineralogy of rare earth elements Chapter 7. Rare earth elements in sedimentary rocks: Influence of provenance and sedimentary processes. In B. R. Lipin, G. A. McKay (Eds.), (pp. 169-200). De Gruyter.10.1515/9781501509032-010

[CR59] McQuatters-Gollop A, Raitsos DE, Edwards M, Pradhan Y, Mee LD, Lavender SJ, Attrill MJ (2007). A long-term chlorophyll data set reveals regime shift in North Sea phytoplankton biomass unconnected to nutrient trends. Limnology and oceanography.

[CR60] Meisch H-U, Bielig H-J (1975). Effect of vanadium on growth, chlorophyll formation and iron metabolism in unicellular green algae. Archives of microbiology.

[CR61] Morel FMM (2008). The co-evolution of phytoplankton and trace element cycles in the oceans. Geobiology.

[CR62] Morel, F. M. M., Price, N. M (2003) The biogeochemical cycles of trace metals in the oceans. *Science (New York, N.Y.)*, *300*(5621), 944–947 10.1126/science.1083545 10.1126/science.1083545 12738853

[CR63] Mortelmans, J., Deneudt, K., Cattrijsse, A., Beauchard, O., Daveloose, I., Vyverman, W., Vanaverbeke, J., Timmermans, K., Peene, J., Roose, P., Knockaert, M., Chou, L., Sanders, R., Stinchcombe, M., Kimpe, P., Lammens, S., Theetaert, H., Gkritzalis, T., Hernandez, F., Mees, J. (2019). Nutrient, pigment, suspended matter and turbidity measurements in the Belgian part of the North Sea. *Scientific data*,* 6*. 10.1038/s41597-019-0032-7 10.1038/s41597-019-0032-7 PMC6472411 30967554

[CR64] OECD, data-explorer.oecd.org, Diagnostic exams, accessed April 2024

[CR65] Pacyna JM, Pacyna EG, Aas W (2009). Changes of emissions and atmospheric deposition of mercury, lead, and cadmium. Atmospheric environment.

[CR66] Pätsch, J., & Kühn, W. (2008). Nitrogen and carbon cycling in the North Sea and exchange with the North Atlantic—A model study Part I Nitrogen budget and fluxes. *Continental shelf research*, *28*(6), 767-787 10.1016/j.csr.2007.12.013

[CR67] Pätsch, J., Lenhart, H.-J., Schütt, M. (2004). Daily loads of nutrients, total alkalinity, dissolved inorganic carbon and dissolved organic carbon of the European continental rivers for the years 1977-2002. Institut für Meereskunde.

[CR68] Paytan A, Mackey KRM, Chen Y, Lima ID, Doney SC, Mahowald N (2009). Toxicity of atmospheric aerosols on marine phytoplankton. Proceedings of the National Academy of Sciences.

[CR69] Pinedo-González P, West AJ, Tovar-Sánchez A, Duarte CM, Marañón E, Cermeño P, González N, Sobrino C, Huete-Ortega M, Fernández A (2015). Surface distribution of dissolved trace metals in the oligotrophic ocean and their influence on phytoplankton biomass and productivity. Global biogeochemical cycles.

[CR70] Przibilla A, Iwainski S, Zimmermann T, Pröfrock D (2023). Impact of storage temperature and filtration method on dissolved trace metal concentrations in coastal water samples. Water environment research.

[CR71] R Core Team (2022). R: A language and environment for statistical computing.

[CR72] Raabe T, Wiltshire KH (2009). Quality control and analyses of the long-term nutrient data from Helgoland Roads, North Sea. Journal of Sea Research.

[CR73] Radach G, Lenhart HJ (1995). Nutrient dynamics in the North Sea: Fluxes and budgets in the water column derived from ERSEM. Netherlands Journal of Sea Research.

[CR74] Radach G, Patsch J (2007). Variability of continental riverine freshwater and nutrient inputs into the North Sea for the years 1977–2000 and its consequences for the assessment of eutrophication. Estuaries and coasts.

[CR75] Redfield AC, Ketchum BH, Richards FA (1963). The influence of organisms on the composition of seawater. The sea.

[CR76] Rehder D (2015). The role of vanadium in biology. Metallomics: Integrated biometal science.

[CR77] Revelle WR (2024). psych: Procedures for Psychological, Psychometric, and Personality Research.

[CR78] Schlesinger WH, Klein EM, Vengosh A (2017). Global biogeochemical cycle of vanadium. Proceedings of the National Academy of Sciences.

[CR79] Schneider AB, Koschinsky A, Kiprotich J, Poehle S, do Nascimento PC (2016). An experimental study on the mixing behavior of Ti, Zr, V and Mo in the Elbe, Rhine and Weser estuaries. Estuarine coastal and shelf science.

[CR80] Schulz KG, Zondervan I, Gerringa LJA, Timmermans KR, Veldhuis MJW, Riebesell U (2004). Effect of trace metal availability on Coccolithophorid calcification. Nature.

[CR81] Skogen MD, Soiland H, Svendsen E (2004). Effects of changing nutrient loads to the North Sea. Journal of Marine Systems.

[CR82] Sohrin Y, Isshiki K, Kuwamoto T, Nakayama E (1987). Tungsten in north pacific waters. Marine chemistry.

[CR83] Sunda, W. G. (2012). Feedback interactions between trace metal nutrients and phytoplankton in the ocean. *Frontiers in microbiology*,* 3*. 10.3389/fmicb.2012.00204 10.3389/fmicb.2012.00204 PMC3369199 22701115

[CR84] Sutorius, M., Mori, C., Greskowiak, J., Boettcher, L., Bunse, C., Dittmar, T., Dlugosch, L., Hintz, N. H., Simon, M., Striebel, M., Pahnke, K. (2022). Rare earth element behaviour in seawater under the influence of organic matter cycling during a phytoplankton spring bloom – A mesocosm study. *Frontiers in marine science*, *9*. 10.3389/fmars.2022.895723

[CR85] Tappin AD, Millward GE, Statham PJ, Burton JD, Morris AW (1995). Trace metals in the central and southern North Sea. Estuarine, coastal and shelf science.

[CR86] Thomas WH, Hollibaugh JT, Seibert DLR, Wallace GT (1980). Toxicity of a mixture of ten metals to phytoplankton. Marine Ecology Progress Series.

[CR87] Thomas H, Bozec Y, Elkalay K, De Baar HJ (2004). Enhanced open ocean storage of CO2 from shelf sea pumping. Science.

[CR88] Thomas H, Bozec Y, de Baar HJW, Elkalay K, Frankignoulle M, Schiettecatte LS, Kattner G, Borges AV (2005). The carbon budget of the North Sea. Biogeosciences.

[CR89] Topcu D, Behrendt H, Brockmann U, Claussen U (2011). Natural background concentrations of nutrients in the German Bight area (North Sea). Environmental monitoring and assessment.

[CR90] Topcu D, Behrendt H, Brockmann U, Claussen U (2011). Natural background concentrations of nutrients in the German Bight area (North Sea). Environmental monitoring and assessment.

[CR91] Trapasso G, Chiesa S, Freitas R, Pereira E (2021). What do we know about the ecotoxicological implications of the rare earth element gadolinium in aquatic ecosystems?. The science of the total environment.

[CR92] Tribovillard N, Algeo TJ, Lyons T, Riboulleau A (2006). Trace metals as paleoredox and paleoproductivity proxies: An update. Chemical geology.

[CR93] Twining BS, Baines SB (2013). The trace metal composition of marine phytoplankton. Annual review of marine science.

[CR94] Ussher SJ, Worsfold PJ, Achterberg EP, Laes A, Blain S, Laan P, Baar HJW (2007). Distribution and redox speciation of dissolved iron on the European continental margin. Limnology and oceanography.

[CR95] van Beusekom, J. E. E., Carstensen, J., Dolch, T., Grage, A., Hofmeister, R., Lenhart, H., Kerimoglu, O., Kolbe, K., Patsch, J., Rick, J., Ronn, L., Ruiter, H. (2019). Wadden Sea eutrophication: Long-term trends and regional differences. *Frontiers in marine science*,* 6*. 10.3389/fmars.2019.00370

[CR96] van der Zee C, Chou L (2005). Seasonal cycling of phosphorus in the southern bight of the North Sea. Biogeosciences.

[CR97] Wickham, H., Averick, M., Bryan, J., Chang, W., McGowan, L., François, R., et al. (2019). Welcome to the Tidyverse. *Journal of Open Source Software, 4*(43), 1686. 10.21105/joss.01686.

[CR98] Wiltshire KH, Kraberg A, Bartsch I, Boersma M, Franke H-D, Freund J, Gebühr C, Gerdts G, Stockmann K, Wichels A (2010). Helgoland roads, North Sea: 45 years of change. Estuaries and coasts.

[CR99] Wiltshire KH, Boersma M, Carstens K, Kraberg AC, Peters S, Scharfe M (2015). Control of phytoplankton in a shelf sea: Determination of the main drivers based on the Helgoland roads time series. Journal of Sea Research.

[CR100] Winther NG, Johannessen JA (2006). North Sea circulation: Atlantic inflow and its destination. Journal of Geophysical Research: Oceans.

[CR101] Wong MY, Rathod SD, Marino R, Li L, Howarth RW, Alastuey A (2021). Anthropogenic perturbations to the atmospheric molybdenum cycle. Global Biogeochemical Cycles.

[CR102] Yang T, Chen Y, Zhou S, Li H (2019). Impacts of aerosol copper on marine phytoplankton: A review. Atmosphere.

[CR103] Zhang X, Ward BB, Sigman DM (2020). Global nitrogen cycle: Critical enzymes, organisms, and processes for nitrogen budgets and dynamics. Chemical reviews.

