# Supplementary material for: Dissolved trace elements and nutrients in the North Sea—a current baseline
Source: Environ Monit Assess. 2024 May 11;196(6):539. doi: 10.1007/s10661-024-12675-2 (PMC11088546; doi:10.1007/s10661-024-12675-2)
Supplement: Supplementary file 2 — Supplementary file2 (DOCX 10823 KB) [file 10661_2024_12675_MOESM2_ESM.docx]

**Supporting Information**

**Dissolved trace elements and nutrients in the North Sea – a current baseline**

Anna Siems^a,b^ , Tristan Zimmermann^a^ , Tina Sanders^c^ , Daniel Pröfrock^a,*^

^a^ Helmholtz-Zentrum Hereon, Institute of Coastal Environmental Chemistry, Geesthacht, Germany

^b^ Universität Hamburg, Department of Chemistry, Inorganic and Applied Chemistry, Hamburg, Germany

^c^ Helmholtz-Zentrum Hereon, Institute of Carbon Cycles, Geesthacht, Germany

*corresponding author: daniel.proefrock@hereon.de

Table S2 Measured isotopes, concentrations and standard deviations of reference materials (QC, NASS-7) and medians and MAD of blank concentrations and LOD and LOQ values. Number of measurements is shown in parentheses. * indicates that BEC (background equivalent concentration) was higher than the LOD and hence the BEC is shown.

| Element | Isotope | QC / ng L^-1^ | NASS-7 / ng L^‑1^ | Blank / ng L^-1^ | LOD / ng L^-1^ | LOQ / ng L^‑1^ |
| --- | --- | --- | --- | --- | --- | --- |
| V | 51 | 28.7 ± 2.9 (138) | 1420 ± 50 (33) | 0.11 ± 0.17 (35) | 1.48* | 1.8 |
| Mn | 55 | 24.9 ± 0.8 (137) | 782 ± 20 (33) | 11 ± 10 (35) | 41 | 111 |
| Fe | 56 | 218 ± 22 (137) | 360 ± 40 (33) | 60 ± 60 (35) | 230 | 610 |
| Co | 59 | 25.9 ± 0.9 (138) | 15 ± 0.4 (33) | 0.07 ± 0.04 (35) | 0.18 | 0.44 |
| Ni | 60 | 24.6 ± 1.6 (138) | 255 ± 8 (33) | 2.8 ± 2.9 (35) | 11.6 | 32.2 |
| Cu | 65 | 24.8 ± 1.6 (138) | 201 ± 6 (33) | 3.5 ± 2.5 (35) | 10.9 | 28.2 |
| Y | 89 | 24.5 ± 1.3 (138) | 19.4 ± 0.5 (33) | 0.07 ± 0.07 (35) | 0.29 | 0.79 |
| Mo | 95 | 28 ± 2.6 (129) | 8000 ± 700 (33) | 0.5 ± 0.5 (35) | 2.3* | 5.7 |
| Cd | 111 | 24.9 ± 0.9 (138) | 15.6 ± 0.5 (33) | 0.07 ± 0.08 (35) | 0.33 | 0.92 |
| La | 139 | 24.6 ± 0.9 (138) | 9.7 ± 0.4 (33) | 0.06 ± 0.05 (35) | 0.23 | 0.61 |
| Ce | 140 | 24.3 ± 1.2 (138) | 3.48 ± 0.17 (33) | 0.08 ± 0.07 (35) | 0.28 | 0.77 |
| Pr | 141 | 24.2 ± 1.3 (138) | 1.38 ± 0.07 (33) | 0.014 ± 0.013 (35) | 0.053 | 0.145 |
| Nd | 146 | 23.7 ± 1.4 (138) | 5.95 ± 0.23 (33) | 0.05 ± 0.05 (35) | 0.2 | 0.54 |
| Sm | 147 | 23.4 ± 1.8 (138) | 1.04 ± 0.06 (33) | 0.009 ± 0.009 (35) | 0.035 | 0.095 |
| Eu | 153 | 23.7 ± 1.8 (138) | 0.24 ± 0.04 (33) | 0.0017 ± 0.0026 (35) | 0.0097* | 0.027 |
| Gd | 157 | 23.6 ± 1.7 (138) | 1.47 ± 0.08 (33) | 0.024 ± 0.027 (35) | 0.104 | 0.292 |
| Tb | 159 | 24.5 ± 2.4 (138) | 0.23 ± 0.03 (32) | 0.0007 ± 0.001 (35) | 0.0089* | 0.011 |
| Dy | 163 | 23.6 ± 1.7 (138) | 1.53 ± 0.1 (33) | 0.006 ± 0.007 (35) | 0.028 | 0.081 |
| Ho | 165 | 23.9 ± 1.7 (138) | 0.41 ± 0.04 (33) | 0.0011 ± 0.0017 (35) | 0.0097* | 0.018 |
| Er | 166 | 23.7 ± 1.8 (138) | 1.29 ± 0.09 (33) | 0.003 ± 0.004 (35) | 0.015 | 0.044 |
| Tm | 169 | 23.4 ± 2 (138) | 0.21 ± 0.04 (33) | 0 ± 0 (35) | 0.023* |  |
| Yb | 172 | 23.1 ± 2.8 (138) | 1.25 ± 0.1 (33) | 0 ± 0 (35) | 0.04* |  |
| Lu | 175 | 23.5 ± 3.4 (138) | 0.23 ± 0.07 (32) | 0 ± 0 (35) | 0.07* |  |
| W | 182 | 25 ± 1.8 (138) | 8.1 ± 0.5 (33) | 0.06 ± 0.07 (35) | 0.4* | 0.73 |
| Pb | 208 | 24.2 ± 1.2 (138) | 2.66 ± 0.26 (30) | 1 ± 0.8 (35) | 3.5 | 9.3 |
| U | 238 | 23 ± 2.7 (138) | 2360 ± 160 (33) | 0.1 ± 0.09 (35) | 0.39 | 1.05 |

Table S3 LOD, LOQ calculated from the calibration line (n=4) and measured concentrations of the VKI standard (n=8 for AL557, n=4 for HE586) for the nutrient analysis. Values were calculated for each campaign separately.

| campaign | analyte | LOD / µmol L^‑1^ | LOQ / µmol L^‑1^ | standard / µmol L^‑1^ |
| --- | --- | --- | --- | --- |
| AL557 | NH_4_^+^ | 0.28 | 0.9 | 2.42 ± 0.13 |
| AL557 | NO_2_^-^ | 0.023 | 0.09 | 1.306 ± 0.023 |
| AL557 | PO_4_^3-^ | 0.007 | 0.028 | 2.18 ± 0.03 |
| AL557 | SiO_4_^4-^ | 0.6 | 2 | 16.1 ± 0.9 |
| AL557 | NO_3_^-^ | 0.17 | 0.6 | 10.85 ± 0.18 |
| HE586 | NH_4_^+^ | 0.11 | 0.4 | 2.17 ± 0.03 |
| HE586 | NO_2_^-^ | 0.03 | 0.11 | 1.278 ± 0.007 |
| HE586 | PO_4_^3-^ | 0.017 | 0.06 | 2.034 ± 0.017 |
| HE586 | SiO_4_^4-^ | 0.4 | 1.5 | 14.50 ± 0.10 |
| HE586 | NO_3_^-^ | 0.5 | 1.9 | 10.515 ± 0.016 |

Table S4 Loadings of the analytes on the four rotated components of PCA with varimax rotation. Blue colors indicate negative correlation, red colors positive correlations. Bold numbers indicate changes in the assignment of the variables to the components.

|  | Varimax with measured concentrations | | | | Varimax with concentrations + uncertainty | | | | Varimax with concentrations - uncertainty | | | |
| --- | --- | --- | --- | --- | --- | --- | --- | --- | --- | --- | --- | --- |
|  | comp 1 | comp 2 | comp 3 | comp 4 | comp 1 | comp 2 | comp 3 | comp 4 | comp 1 | comp 2 | comp 3 | comp 4 |
| salinity | 0.44 | 0.19 | -0.81 | -0.06 | 0.43 | 0.19 | -0.81 | -0.09 | 0.40 | 0.18 | -0.83 | -0.01 |
| NO_3_^-^ | 0.07 | 0.85 | -0.28 | -0.13 | 0.10 | 0.86 | -0.24 | -0.16 | 0.05 | 0.85 | -0.33 | -0.04 |
| NO_2_^-^ | -0.18 | 0.60 | 0.06 | 0.19 | -0.24 | 0.62 | 0.03 | 0.21 | -0.13 | 0.63 | 0.09 | 0.20 |
| SiO_4_^4-^ | 0.07 | 0.73 | -0.26 | -0.40 | 0.11 | 0.74 | -0.24 | -0.37 | 0.05 | 0.73 | -0.30 | -0.42 |
| PO_4_^3+^ | 0.28 | 0.66 | **-0.49** | -0.20 | 0.32 | 0.67 | **-0.45** | -0.20 | 0.23 | 0.65 | **-0.53** | -0.17 |
| NH_4_^+^ | -0.01 | 0.15 | 0.11 | 0.80 | -0.06 | 0.17 | 0.09 | 0.80 | 0.03 | 0.15 | 0.15 | 0.78 |
| V | 0.86 | 0.09 | -0.29 | -0.14 | 0.81 | 0.11 | -0.33 | -0.14 | 0.88 | 0.05 | -0.22 | -0.10 |
| Mn | -0.28 | -0.12 | **0.48** | 0.57 | -0.26 | -0.14 | **0.48** | 0.57 | -0.22 | -0.12 | **0.55** | 0.51 |
| Fe | -0.15 | 0.06 | 0.32 | 0.70 | 0.01 | -0.03 | 0.31 | 0.67 | -0.26 | 0.17 | 0.30 | 0.60 |
| Co | -0.24 | -0.17 | 0.76 | 0.41 | -0.25 | -0.19 | 0.73 | 0.45 | -0.15 | -0.14 | 0.83 | 0.32 |
| Ni | -0.10 | 0.08 | 0.89 | 0.25 | -0.10 | 0.09 | 0.86 | 0.27 | 0.02 | 0.09 | 0.89 | 0.21 |
| Cu | -0.28 | -0.13 | 0.79 | 0.33 | -0.29 | -0.13 | 0.76 | 0.35 | -0.19 | -0.08 | 0.84 | 0.26 |
| Mo | 0.74 | 0.27 | 0.27 | -0.20 | 0.73 | 0.20 | 0.36 | -0.21 | 0.72 | 0.20 | 0.22 | -0.16 |
| Cd | 0.38 | 0.65 | 0.09 | 0.29 | 0.44 | 0.62 | 0.14 | 0.26 | 0.44 | 0.60 | 0.11 | 0.28 |
| W | 0.18 | 0.20 | 0.84 | 0.00 | 0.17 | 0.18 | 0.85 | 0.00 | 0.28 | 0.23 | 0.77 | -0.03 |
| Pb | 0.15 | -0.25 | 0.22 | 0.73 | 0.08 | -0.26 | 0.20 | 0.74 | 0.25 | -0.27 | 0.18 | 0.71 |
| U | 0.80 | -0.15 | -0.23 | 0.18 | 0.79 | -0.10 | -0.27 | 0.17 | 0.81 | -0.15 | -0.13 | 0.19 |
| Y | 0.03 | -0.24 | 0.83 | 0.16 | 0.02 | -0.25 | 0.83 | 0.15 | 0.09 | -0.20 | 0.83 | 0.12 |
| sum LREE | -0.03 | -0.38 | 0.72 | 0.28 | -0.03 | -0.38 | 0.71 | 0.27 | 0.00 | -0.36 | 0.72 | 0.25 |
| sum HREE | -0.03 | -0.25 | 0.84 | 0.24 | -0.05 | -0.28 | 0.83 | 0.24 | 0.02 | -0.20 | 0.86 | 0.19 |


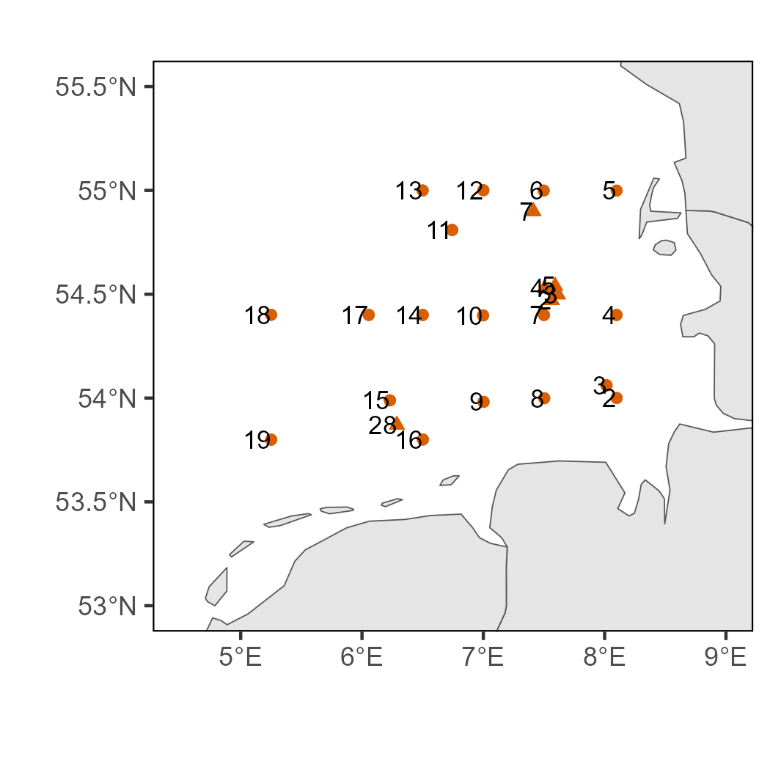

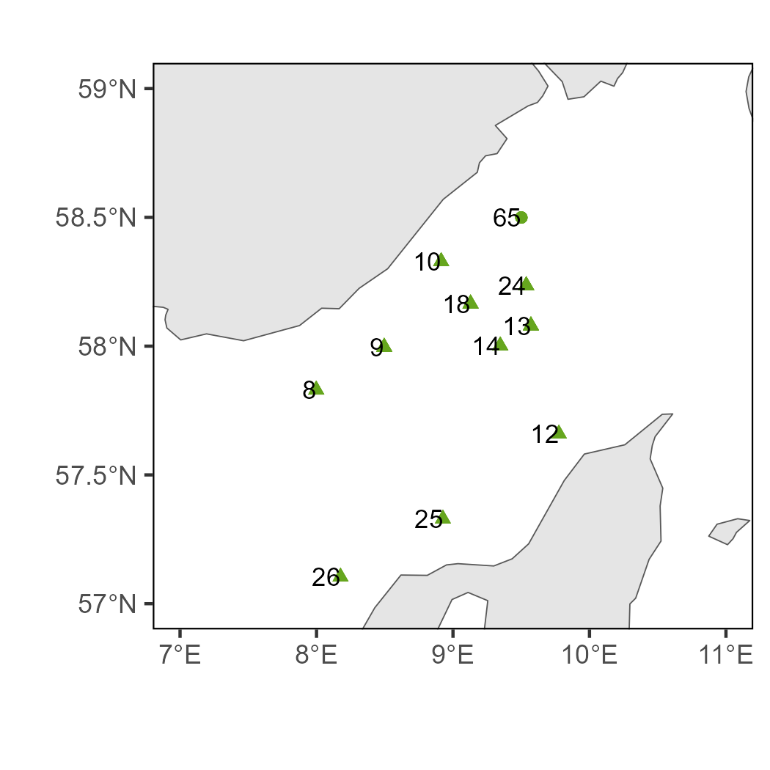

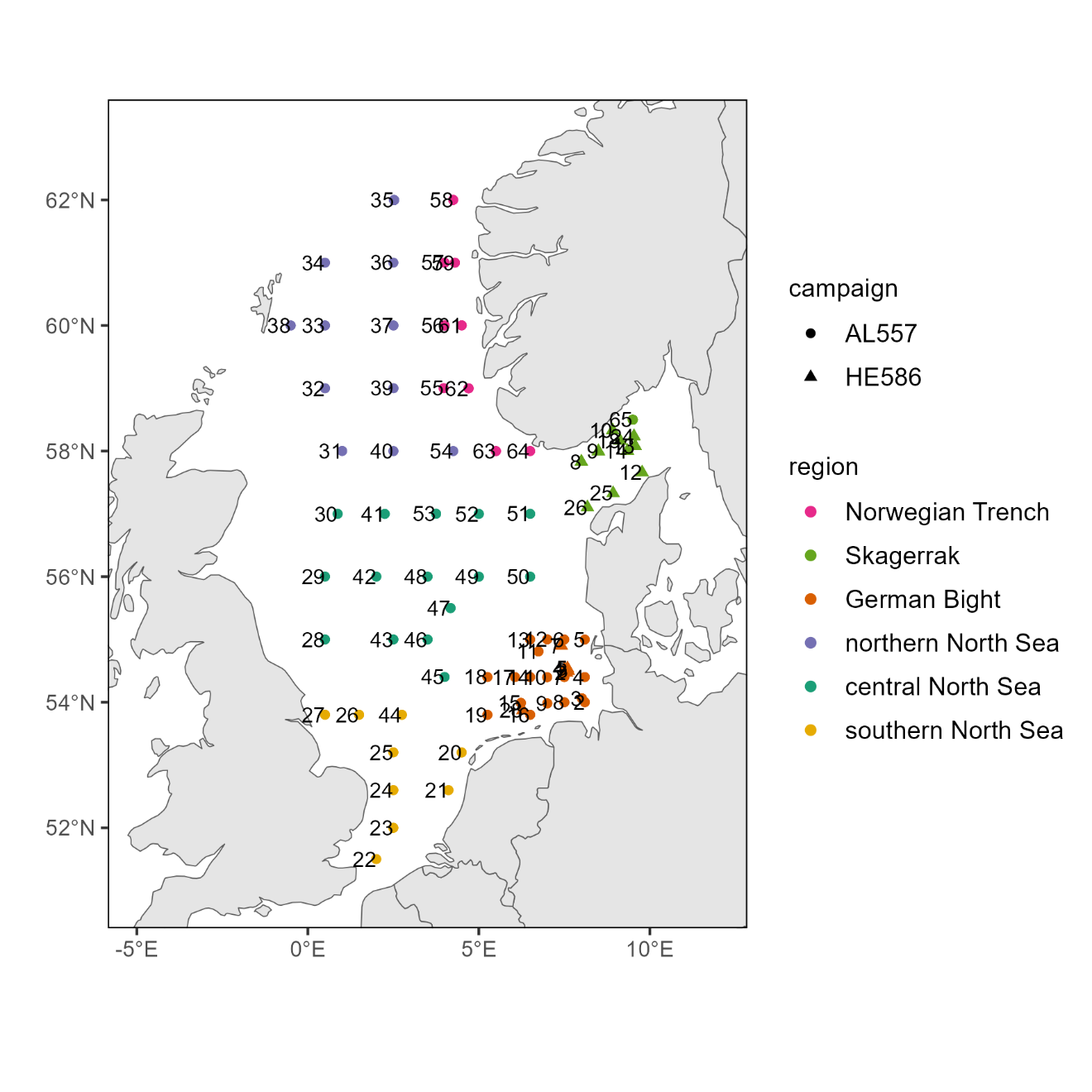


Figure S1 Sampling stations and regions for data interpretation in the North Sea. The circles indicate AL557 samples, triangles indicate HE586 samples. The maps on the left show the stations in the Skagerrak (top) and German Bight (bottom).


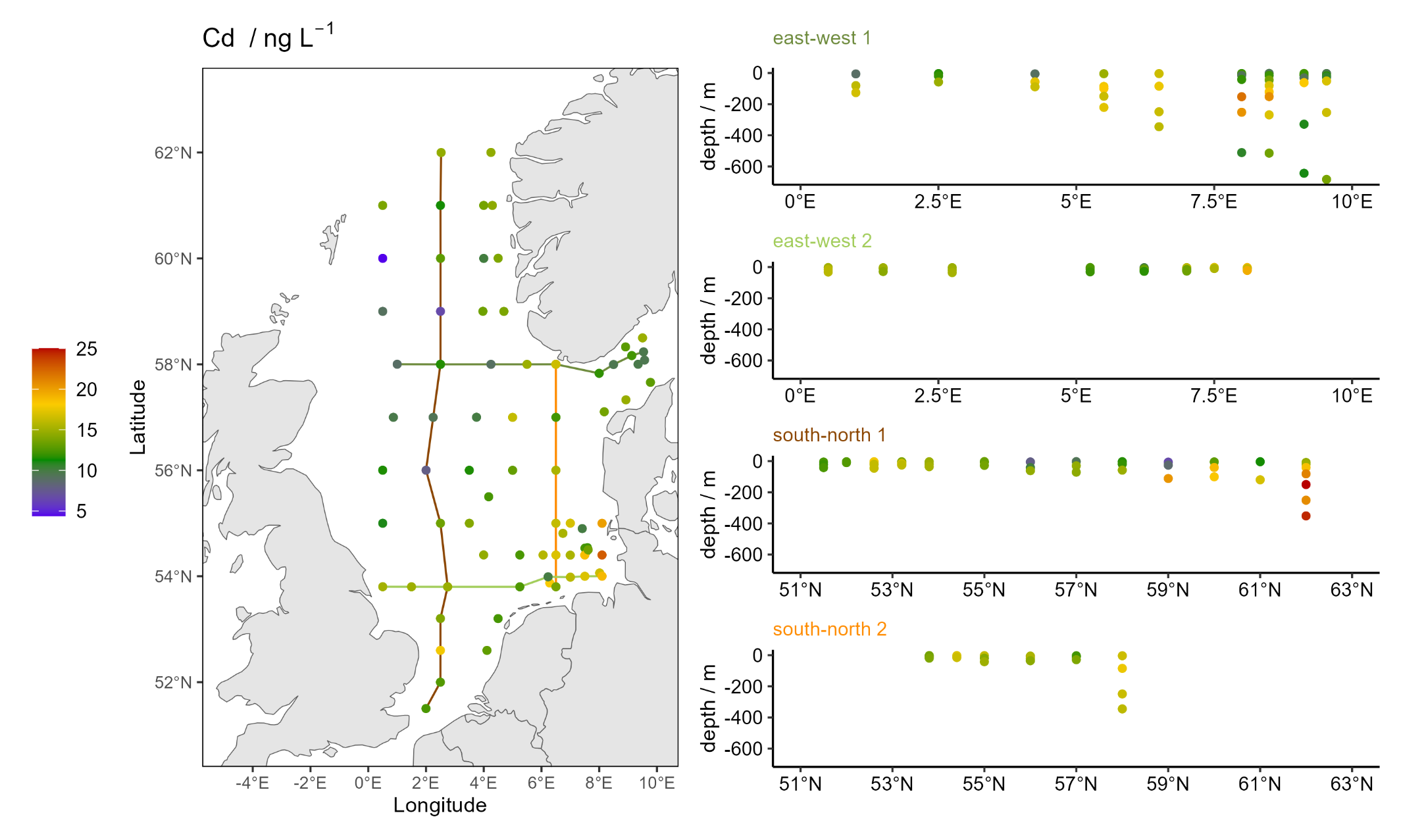


Figure S2 Surface concentrations and depth profiles of Cd across two south-north and two east-west transects. The northernmost transect is east-west 1 and the westernmost transect is south-north 1. x indicates that concentrations were below the LOD and faint points indicate that concentrations were between LOD and LOQ.


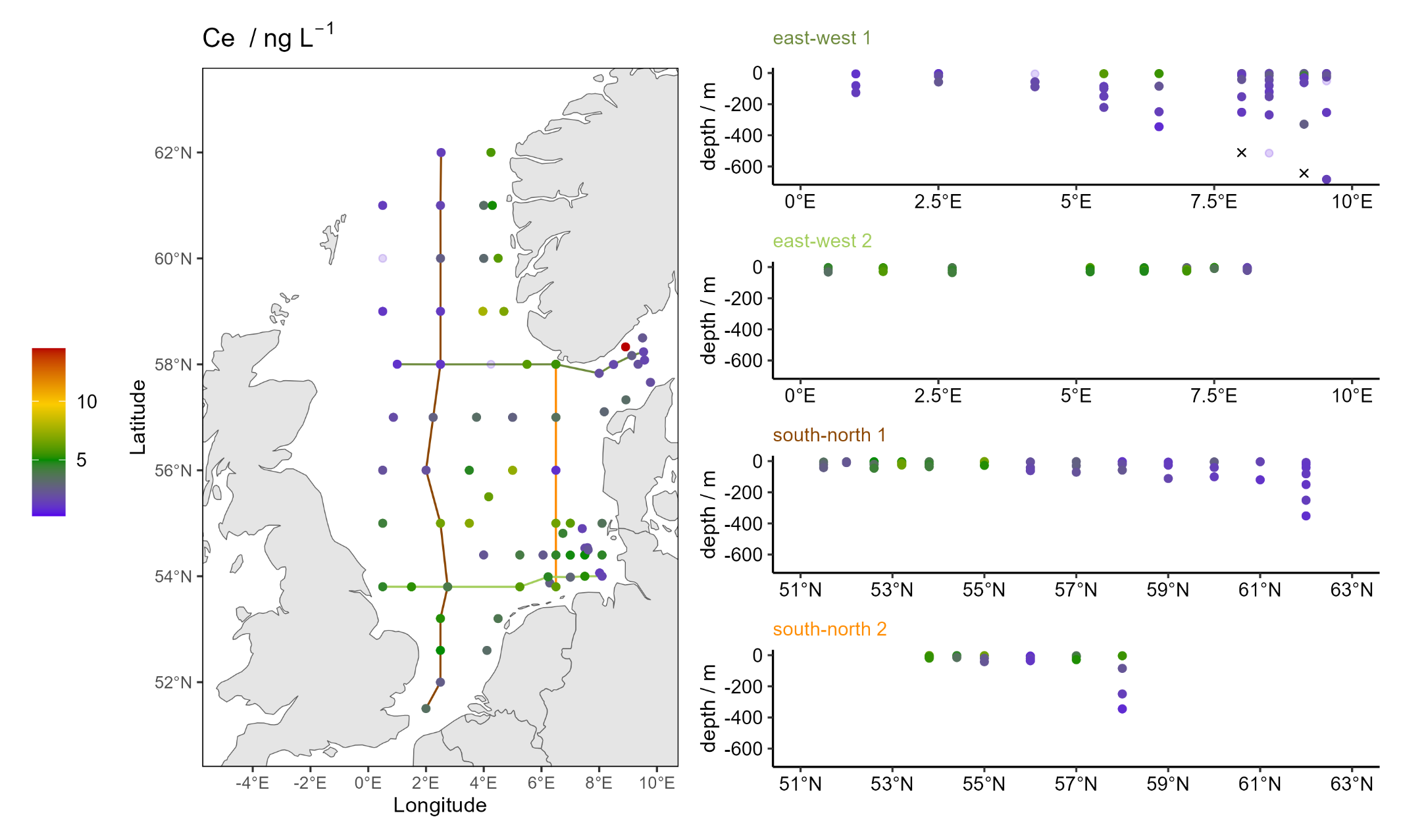


Figure S3 Surface concentrations and depth profiles of Ce across two south-north and two east-west transects. The northernmost transect is east-west 1 and the westernmost transect is south-north 1. x indicates that concentrations were below the LOD and faint points indicate that concentrations were between LOD and LOQ.


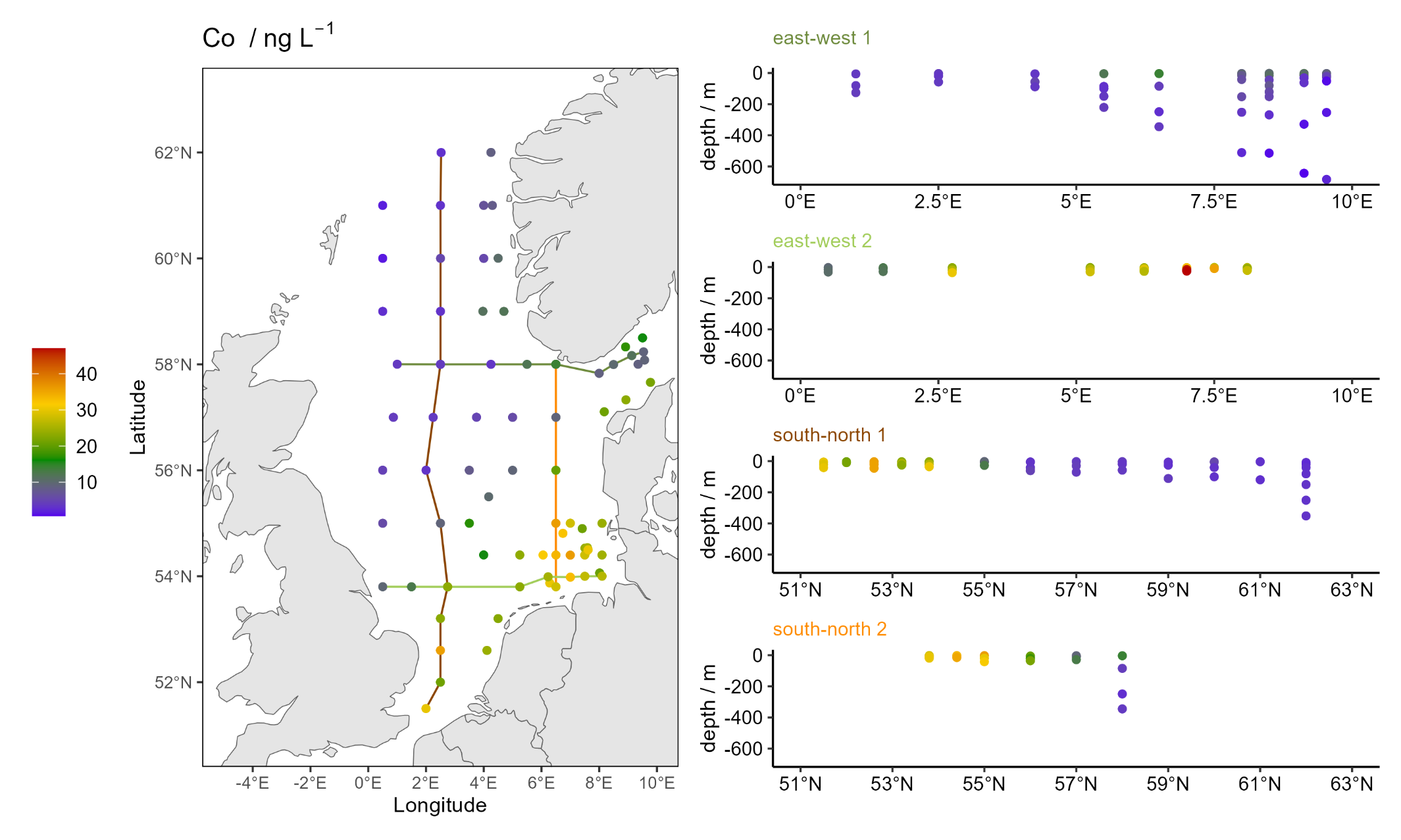


Figure S4 Surface concentrations and depth profiles of Co across two south-north and two east-west transects. The northernmost transect is east-west 1 and the westernmost transect is south-north 1. x indicates that concentrations were below the LOD and faint points indicate that concentrations were between LOD and LOQ.


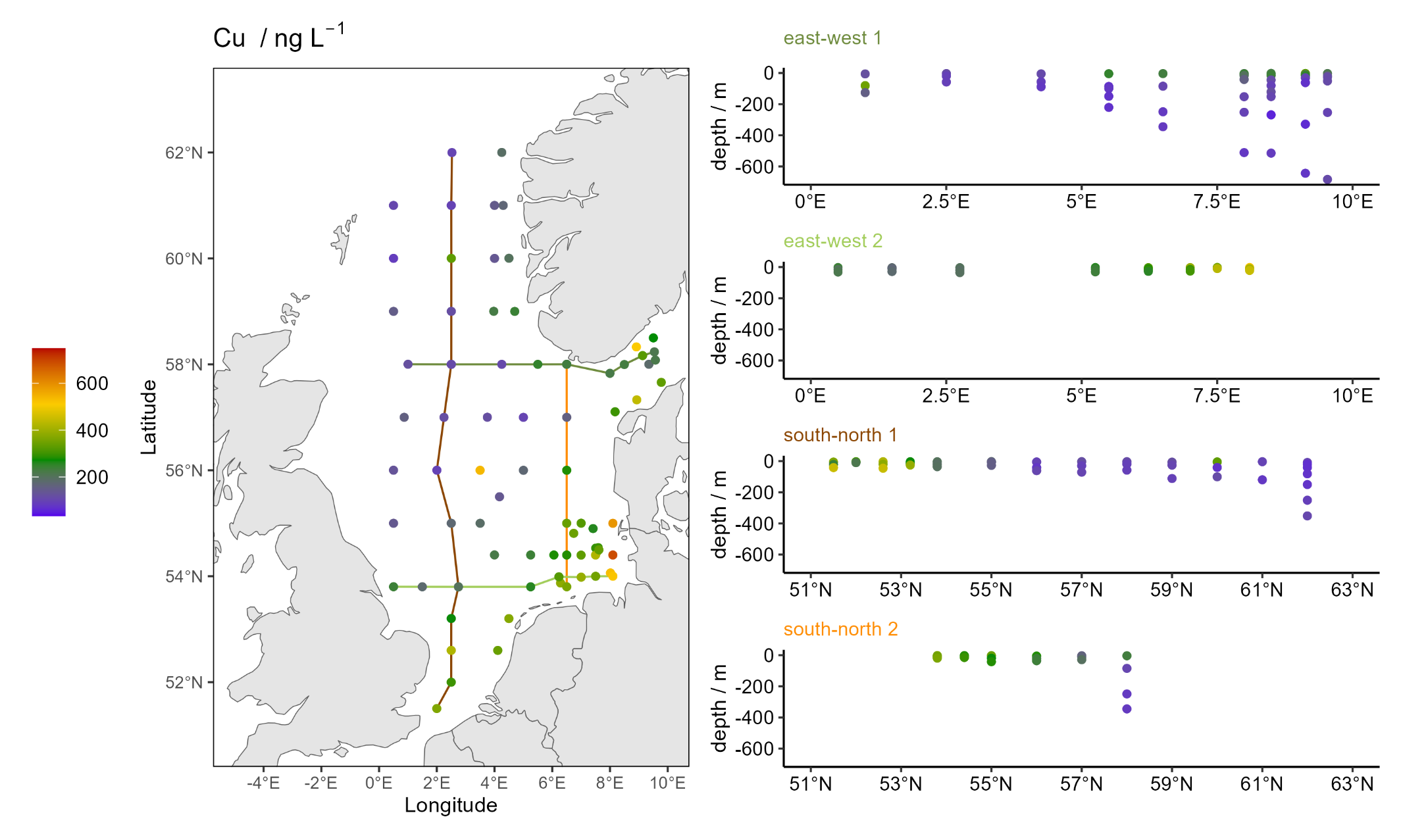


Figure S5 Surface concentrations and depth profiles of Cu across two south-north and two east-west transects. The northernmost transect is east-west 1 and the westernmost transect is south-north 1. x indicates that concentrations were below the LOD and faint points indicate that concentrations were between LOD and LOQ.


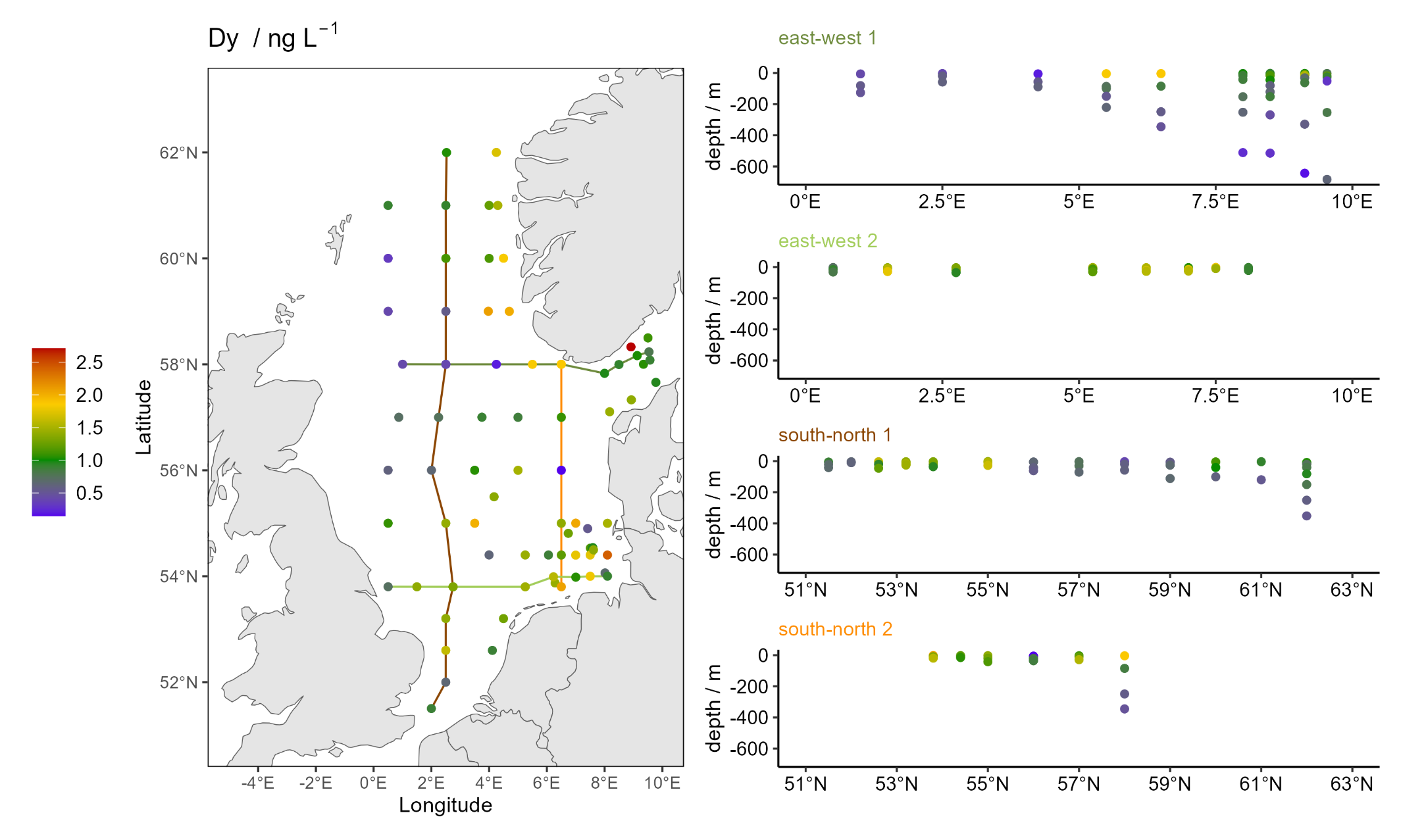


Figure S6 Surface concentrations and depth profiles of Dy across two south-north and two east-west transects. The northernmost transect is east-west 1 and the westernmost transect is south-north 1. x indicates that concentrations were below the LOD and faint points indicate that concentrations were between LOD and LOQ.


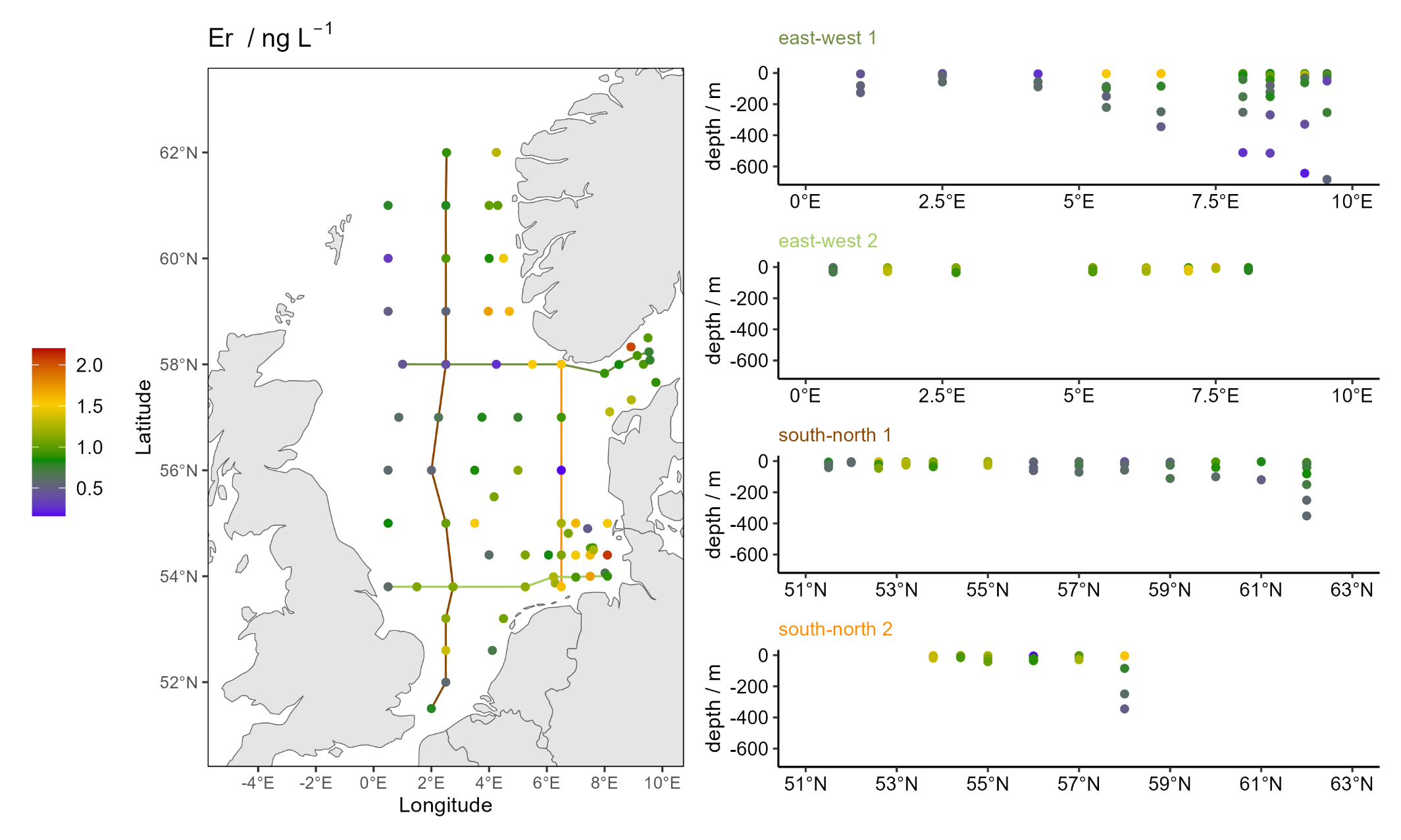


Figure S7 Surface concentrations and depth profiles of Er across two south-north and two east-west transects. The northernmost transect is east-west 1 and the westernmost transect is south-north 1. x indicates that concentrations were below the LOD and faint points indicate that concentrations were between LOD and LOQ.


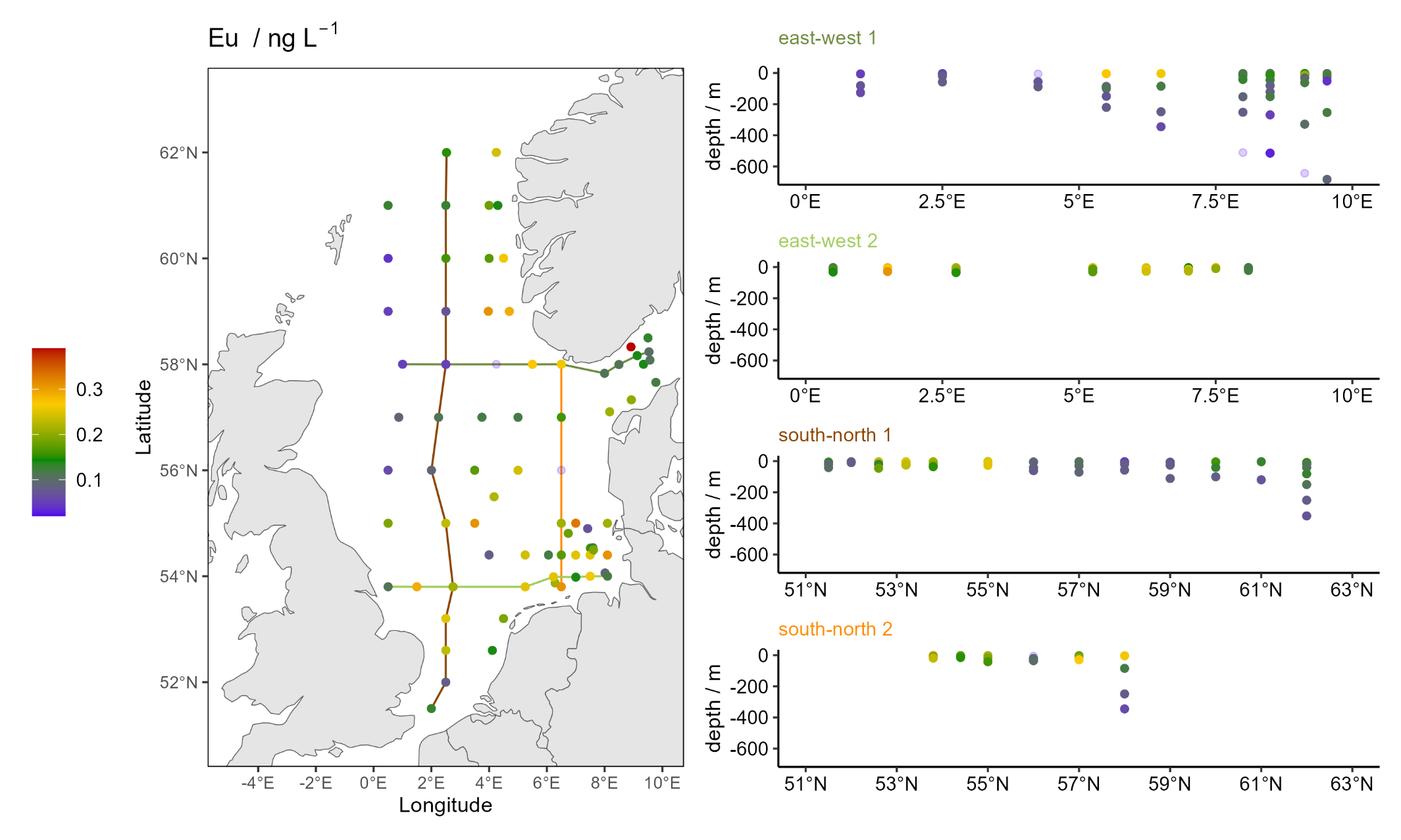


Figure S8 Surface concentrations and depth profiles of Eu across two south-north and two east-west transects. The northernmost transect is east-west 1 and the westernmost transect is south-north 1. x indicates that concentrations were below the LOD and faint points indicate that concentrations were between LOD and LOQ.


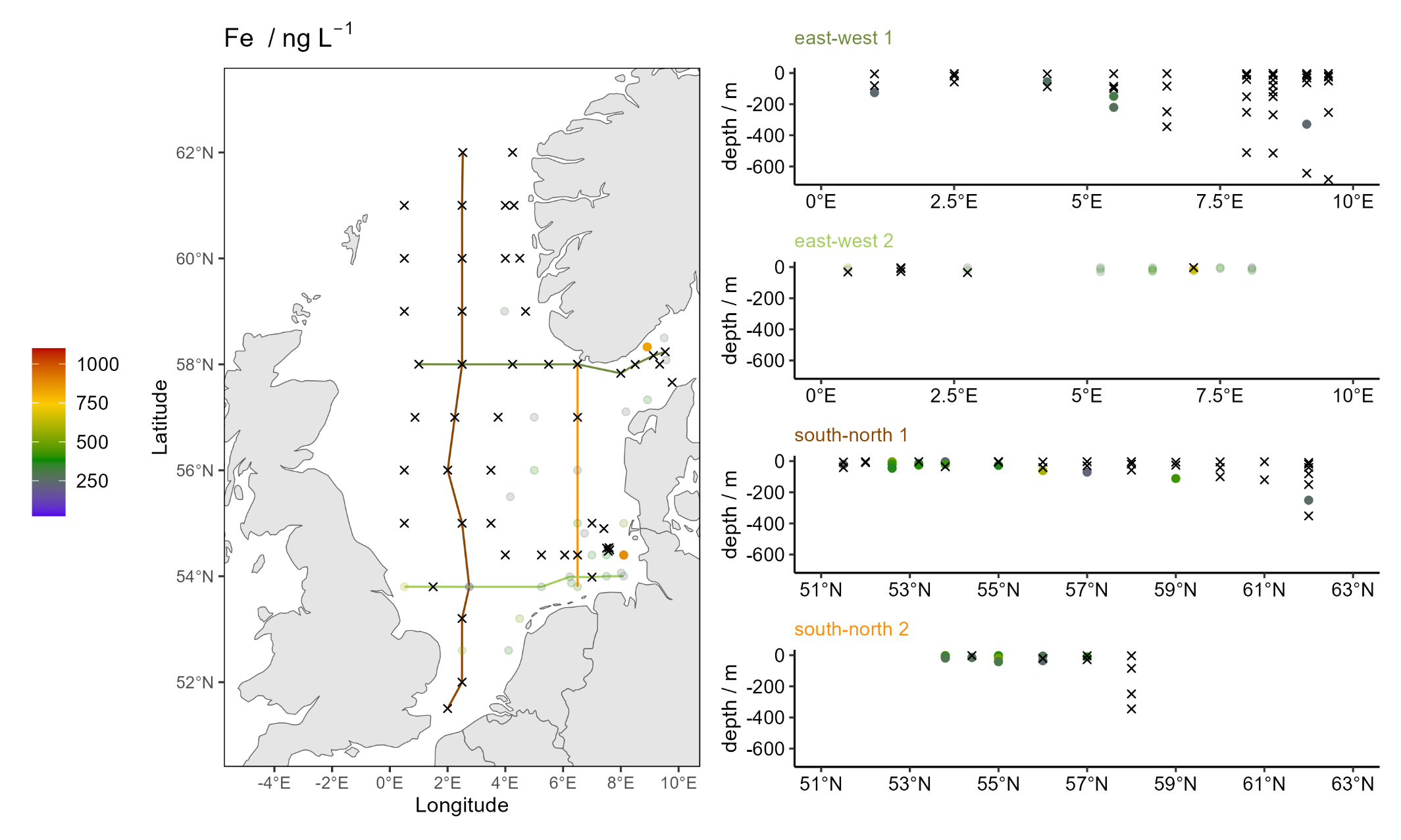


Figure S9 Surface concentrations and depth profiles of Fe across two south-north and two east-west transects. The northernmost transect is east-west 1 and the westernmost transect is south-north 1. x indicates that concentrations were below the LOD and faint points indicate that concentrations were between LOD and LOQ.


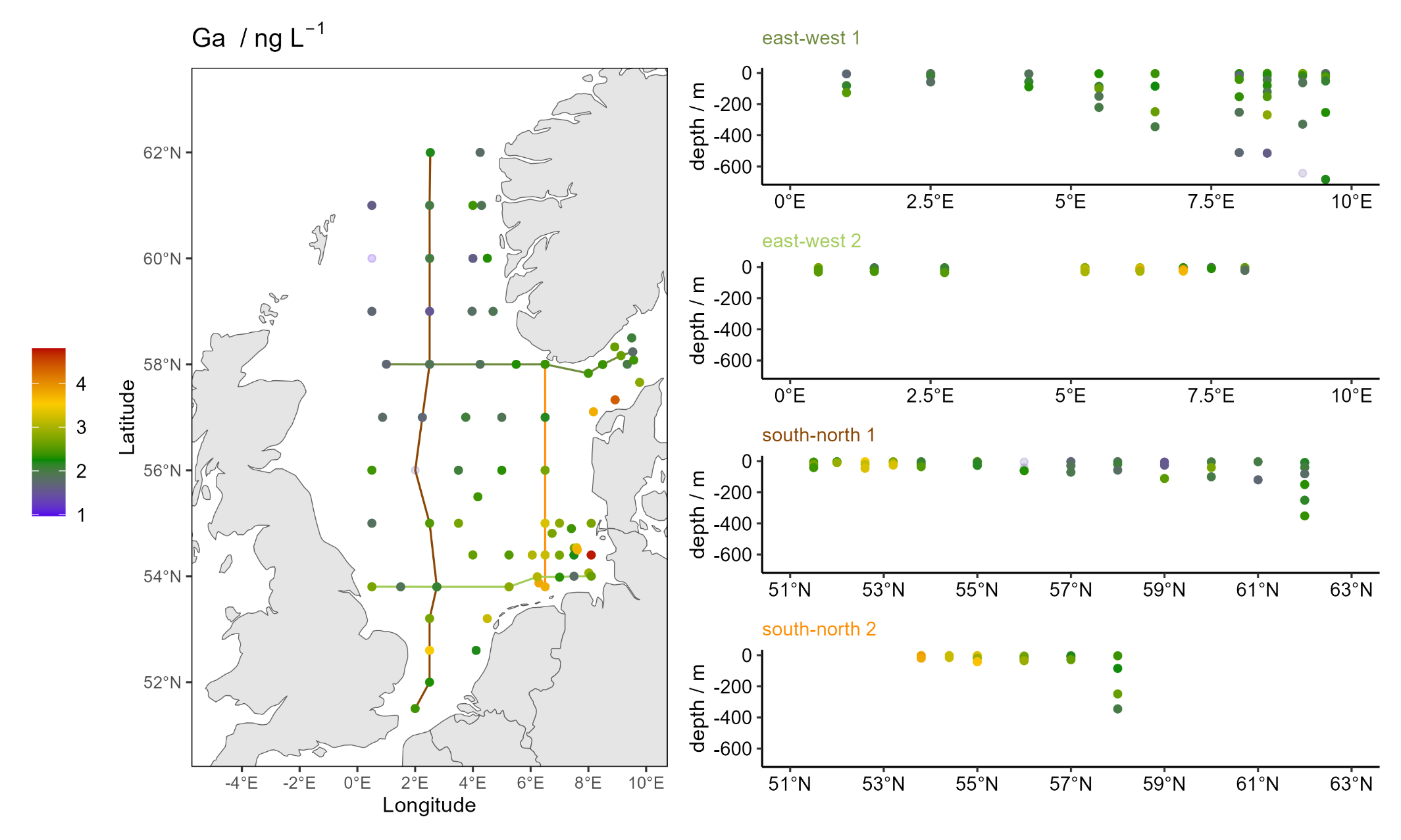


Figure S10 Surface concentrations and depth profiles of Ga across two south-north and two east-west transects. The northernmost transect is east-west 1 and the westernmost transect is south-north 1. x indicates that concentrations were below the LOD and faint points indicate that concentrations were between LOD and LOQ.


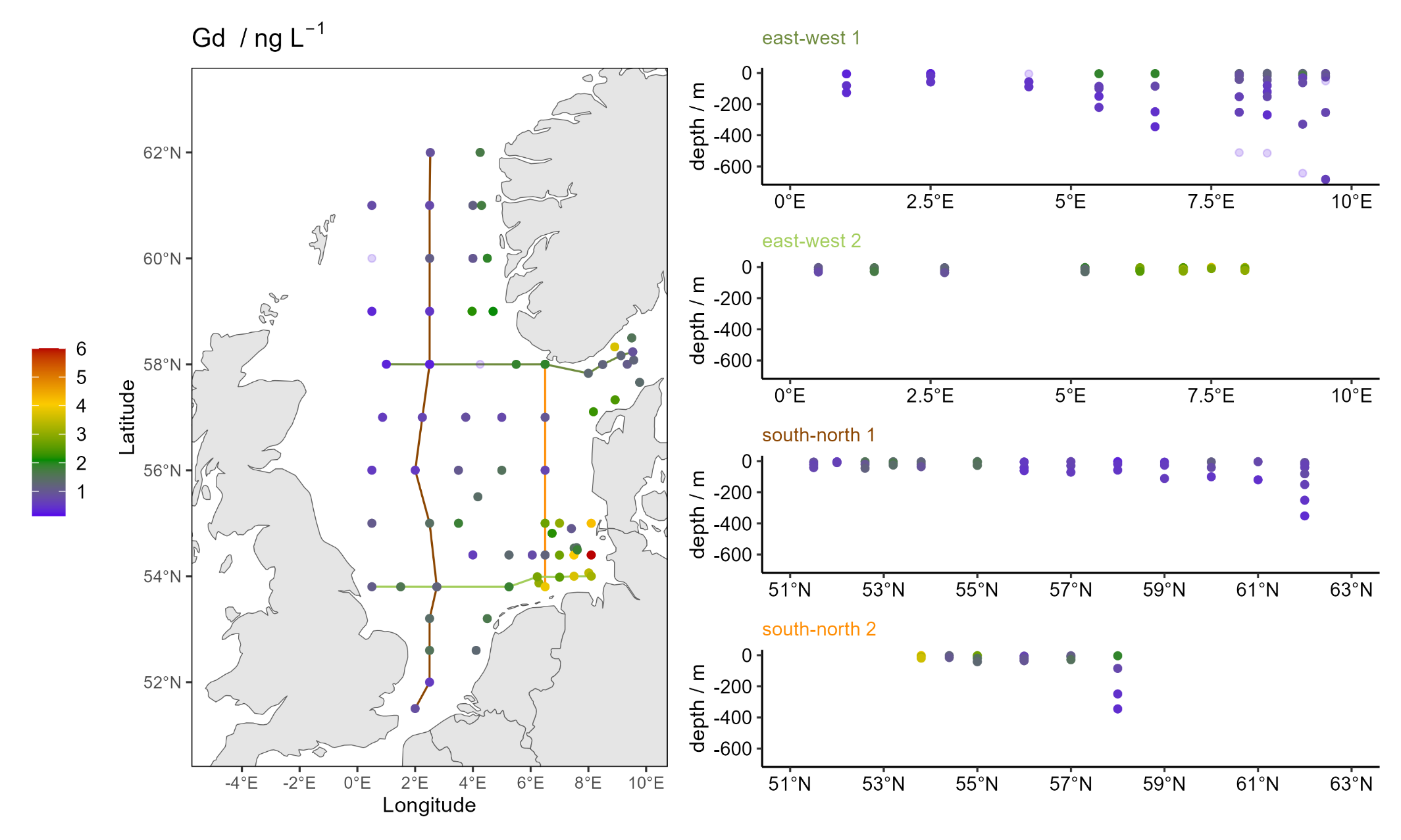


Figure S11 Surface concentrations and depth profiles of Gd across two south-north and two east-west transects. The northernmost transect is east-west 1 and the westernmost transect is south-north 1. x indicates that concentrations were below the LOD and faint points indicate that concentrations were between LOD and LOQ.


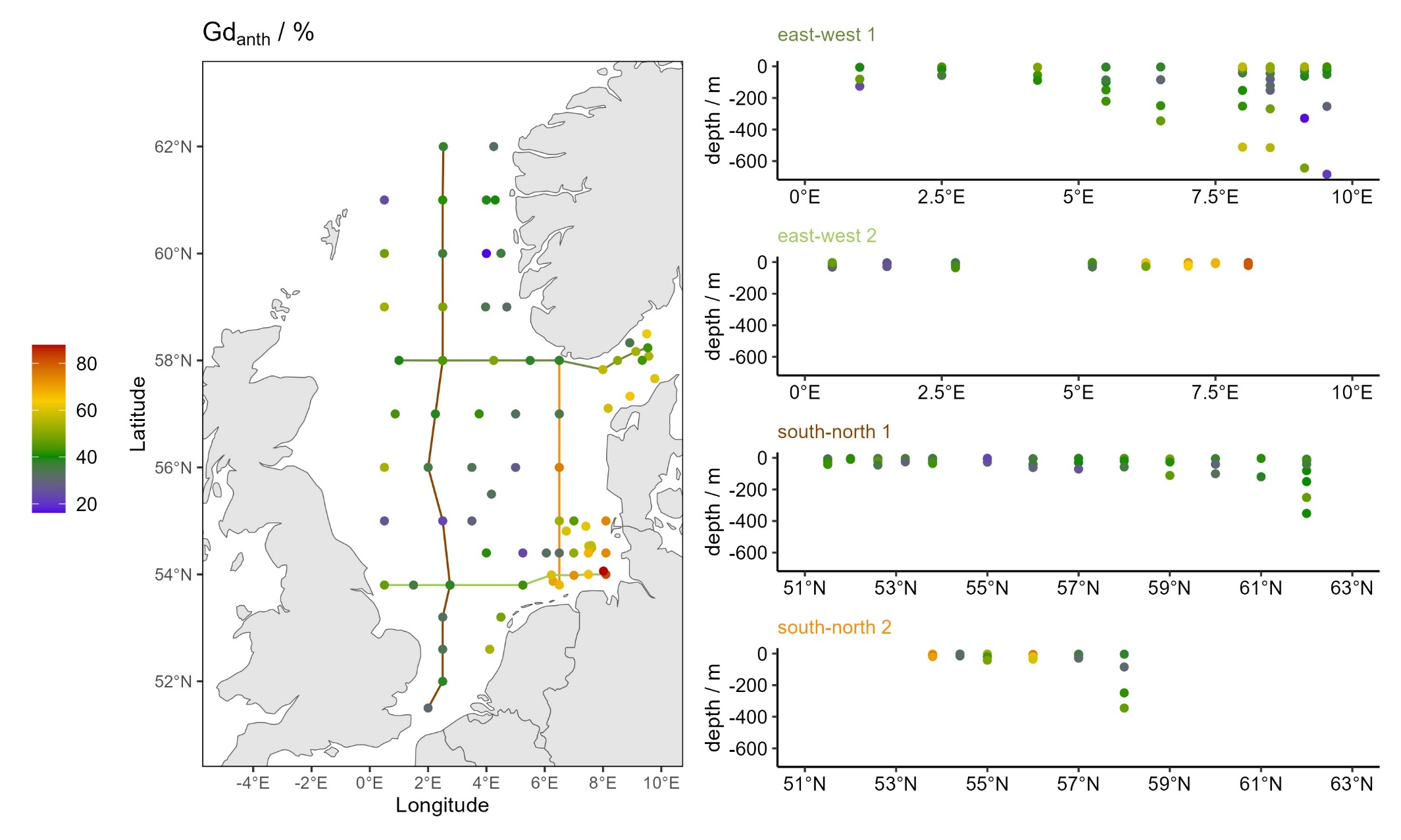


Figure S12 Surface concentrations and depth profiles of Gd_anth_ across two south-north and two east-west transects. The northernmost transect is east-west 1 and the westernmost transect is south-north 1. x indicates that concentrations were below the LOD and faint points indicate that concentrations were between LOD and LOQ.


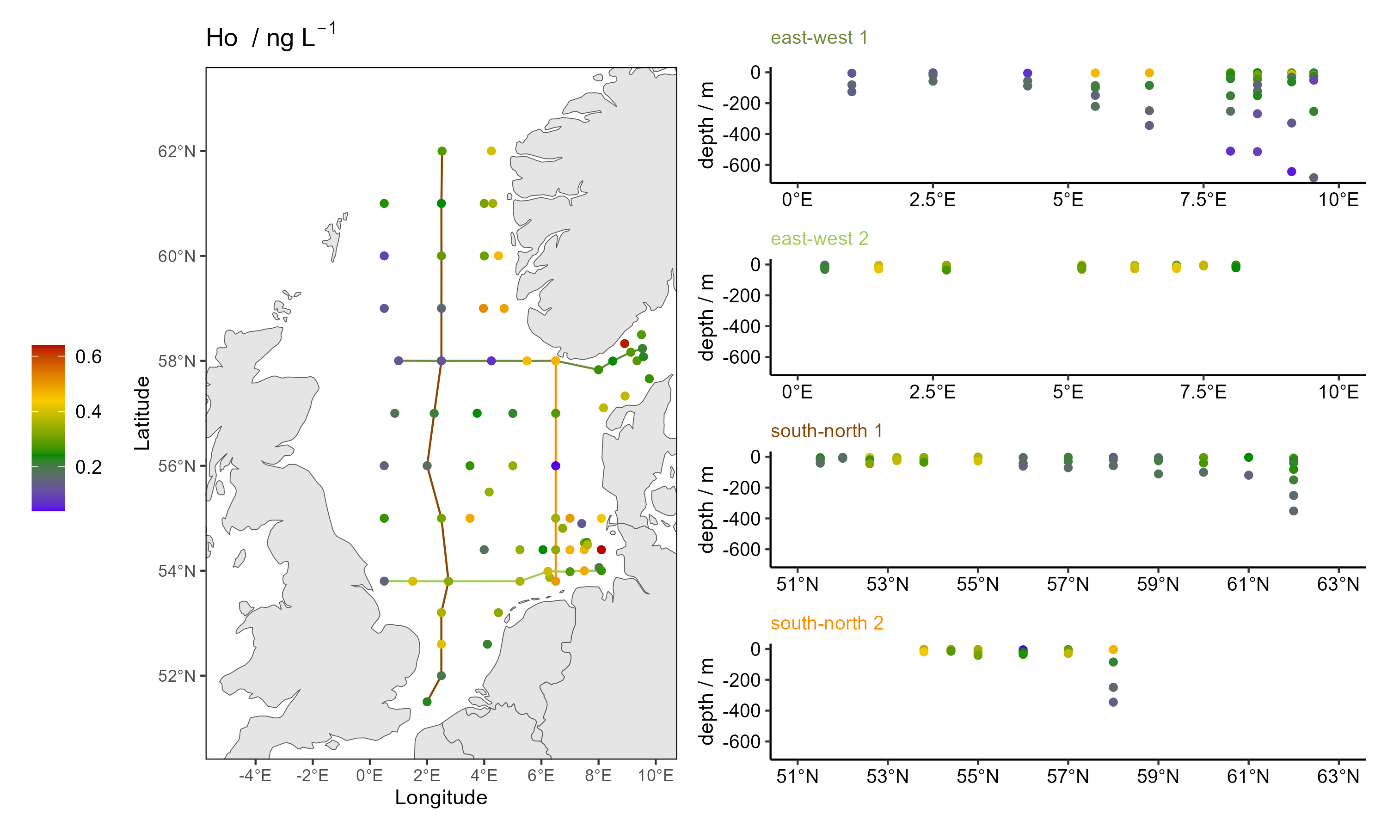


Figure S13 Surface concentrations and depth profiles of Ho across two south-north and two east-west transects. The northernmost transect is east-west 1 and the westernmost transect is south-north 1. x indicates that concentrations were below the LOD and faint points indicate that concentrations were between LOD and LOQ.


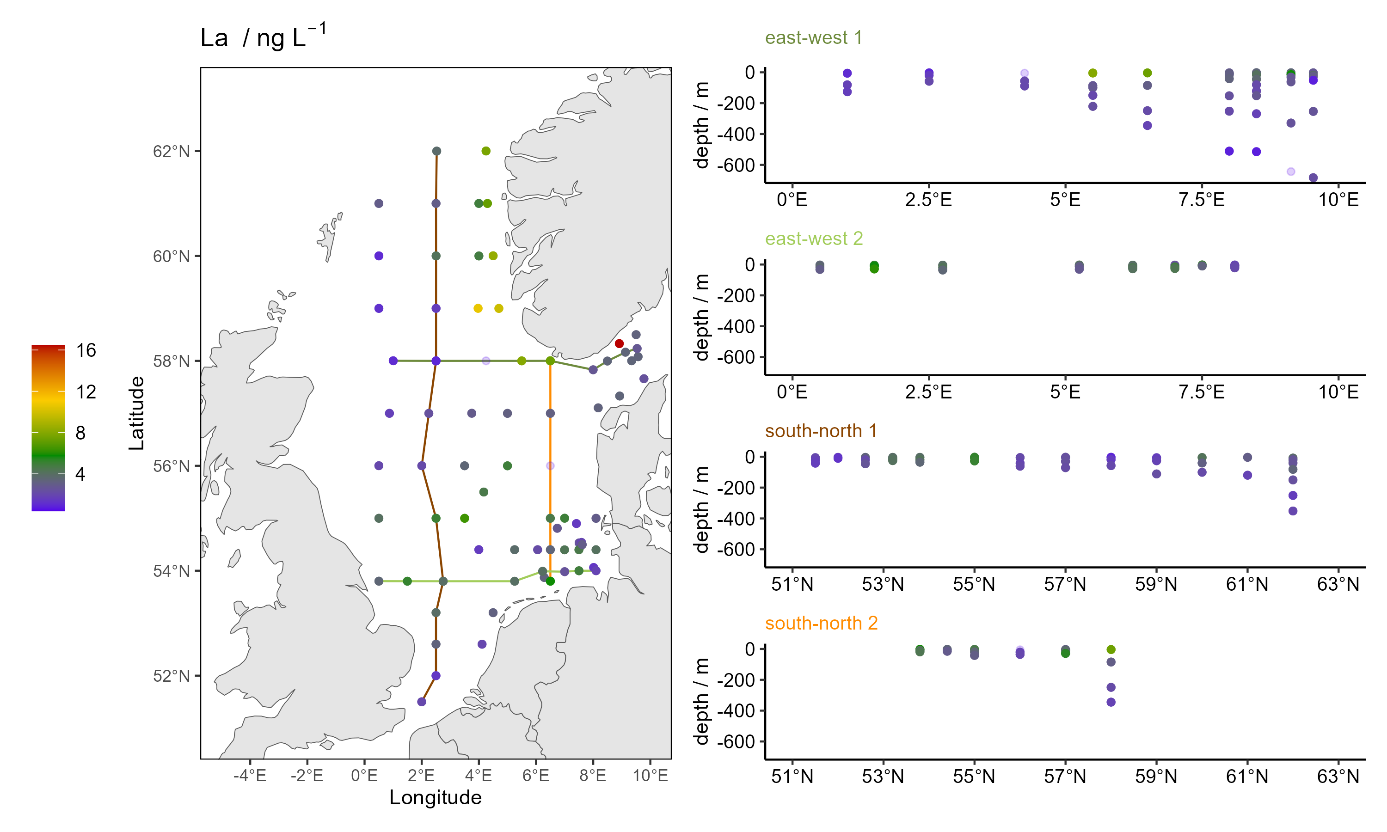


Figure S14 Surface concentrations and depth profiles of La across two south-north and two east-west transects. The northernmost transect is east-west 1 and the westernmost transect is south-north 1. x indicates that concentrations were below the LOD and faint points indicate that concentrations were between LOD and LOQ.


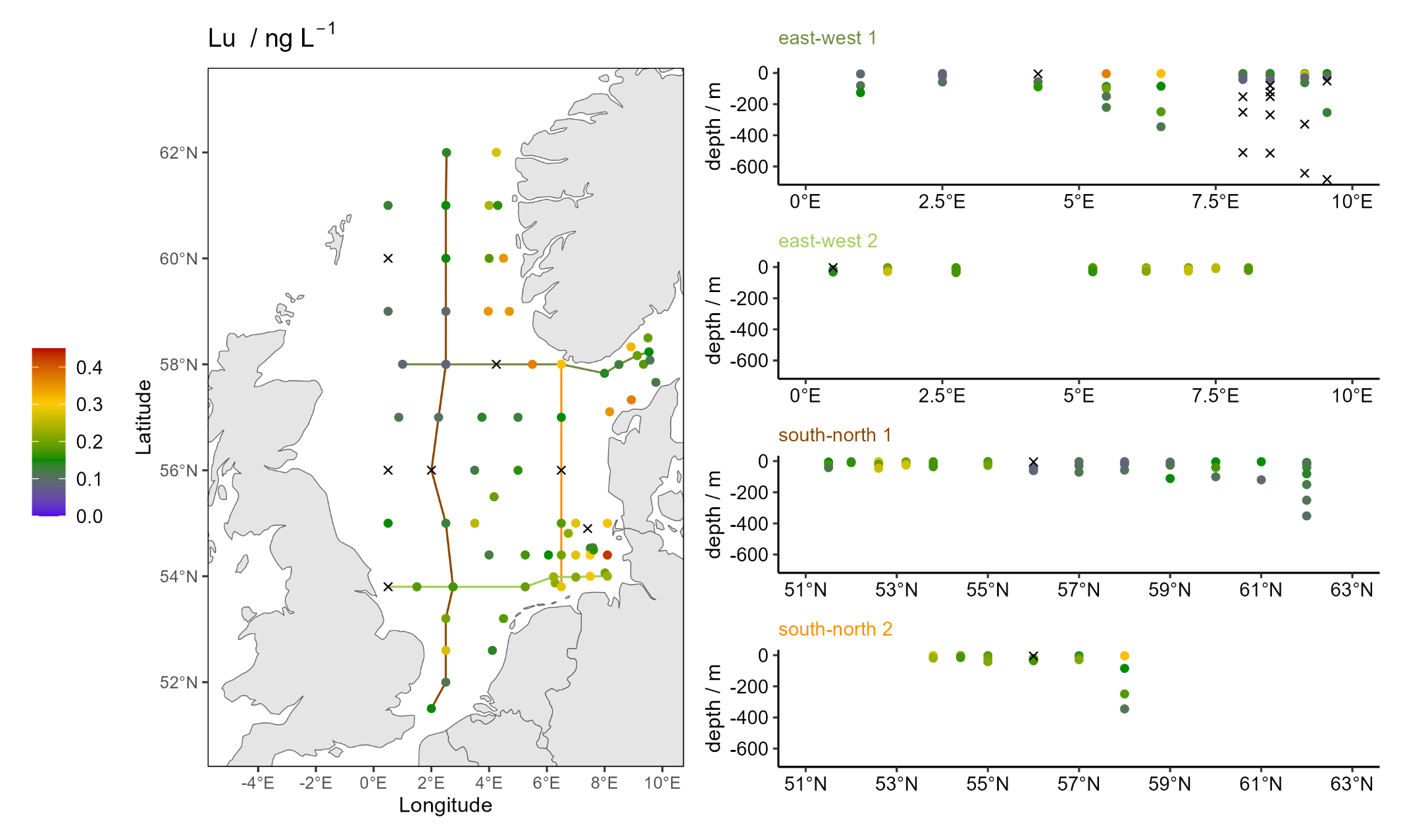


Figure S15 Surface concentrations and depth profiles of Lu across two south-north and two east-west transects. The northernmost transect is east-west 1 and the westernmost transect is south-north 1. x indicates that concentrations were below the LOD and faint points indicate that concentrations were between LOD and LOQ.


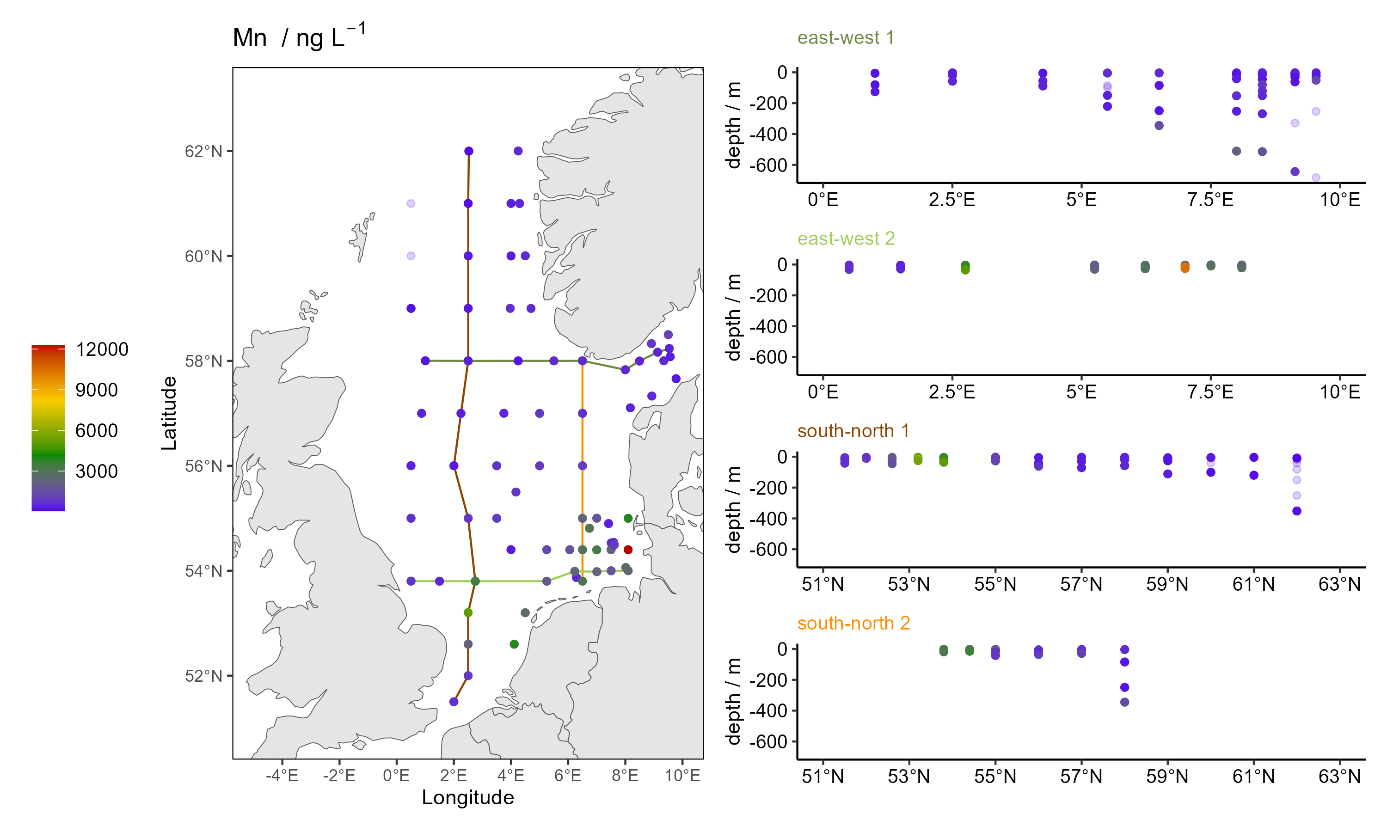


Figure S16 Surface concentrations and depth profiles of Mn across two south-north and two east-west transects. The northernmost transect is east-west 1 and the westernmost transect is south-north 1. x indicates that concentrations were below the LOD and faint points indicate that concentrations were between LOD and LOQ.


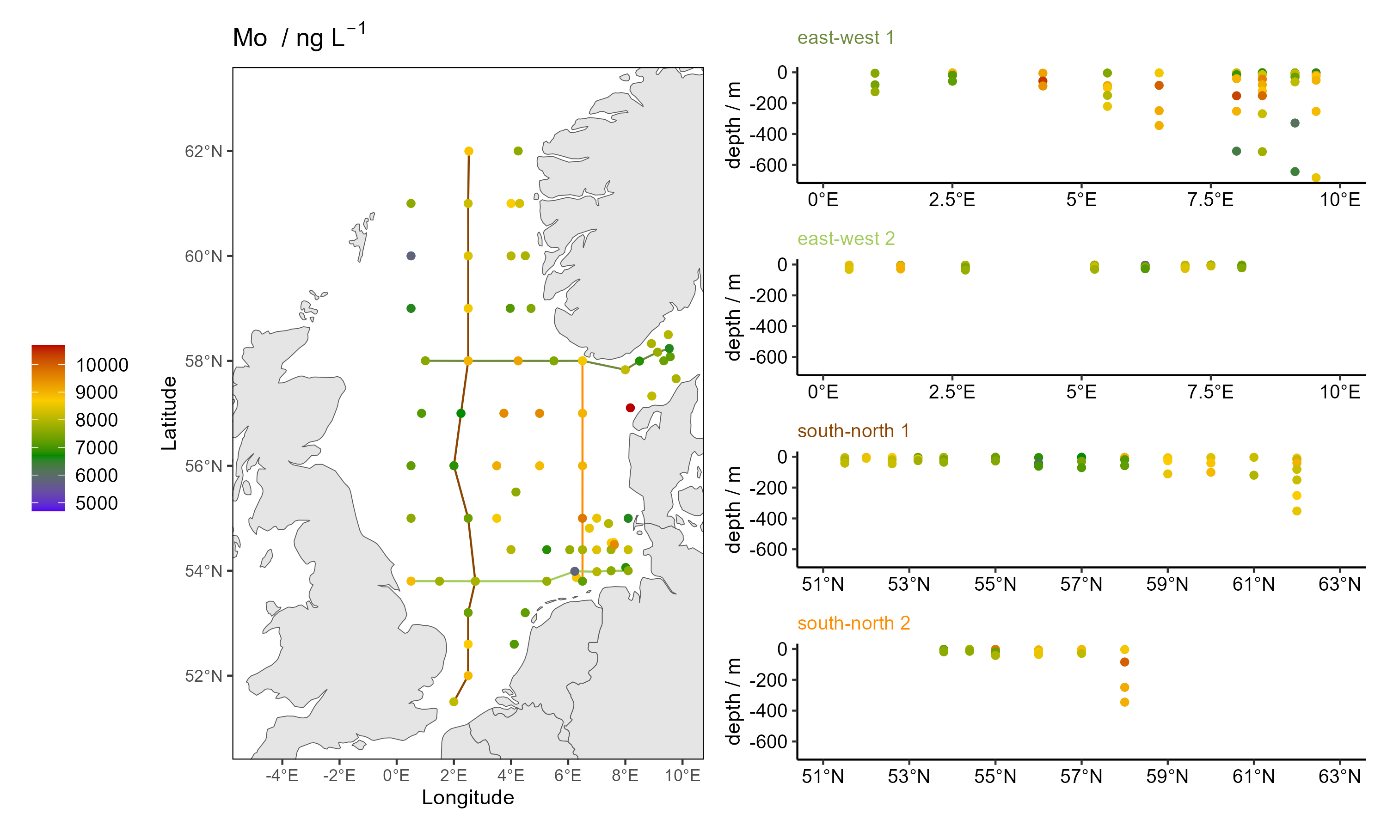


Figure S17 Surface concentrations and depth profiles of Mo across two south-north and two east-west transects. The northernmost transect is east-west 1 and the westernmost transect is south-north 1. x indicates that concentrations were below the LOD and faint points indicate that concentrations were between LOD and LOQ.


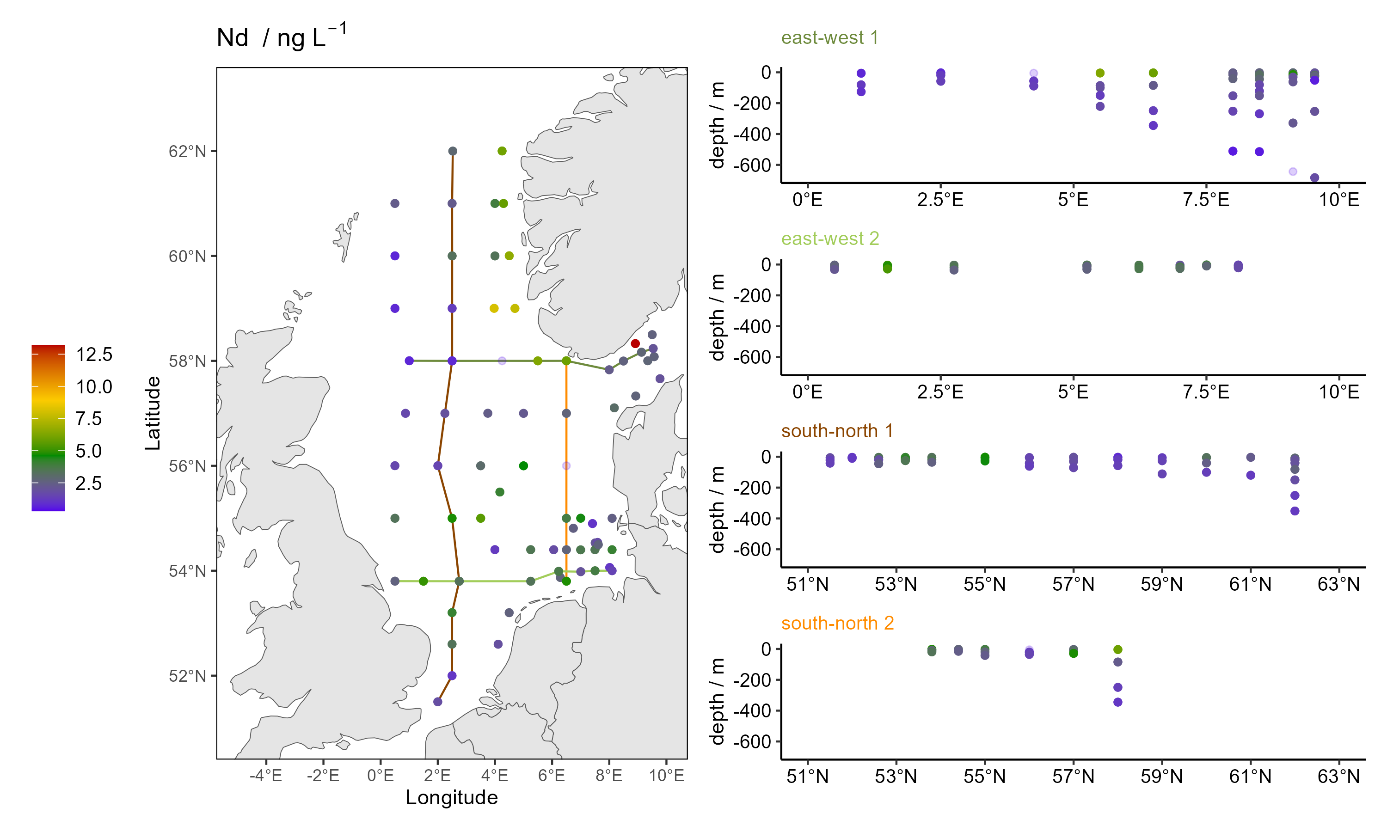


Figure S18 Surface concentrations and depth profiles of Nd across two south-north and two east-west transects. The northernmost transect is east-west 1 and the westernmost transect is south-north 1. x indicates that concentrations were below the LOD and faint points indicate that concentrations were between LOD and LOQ.


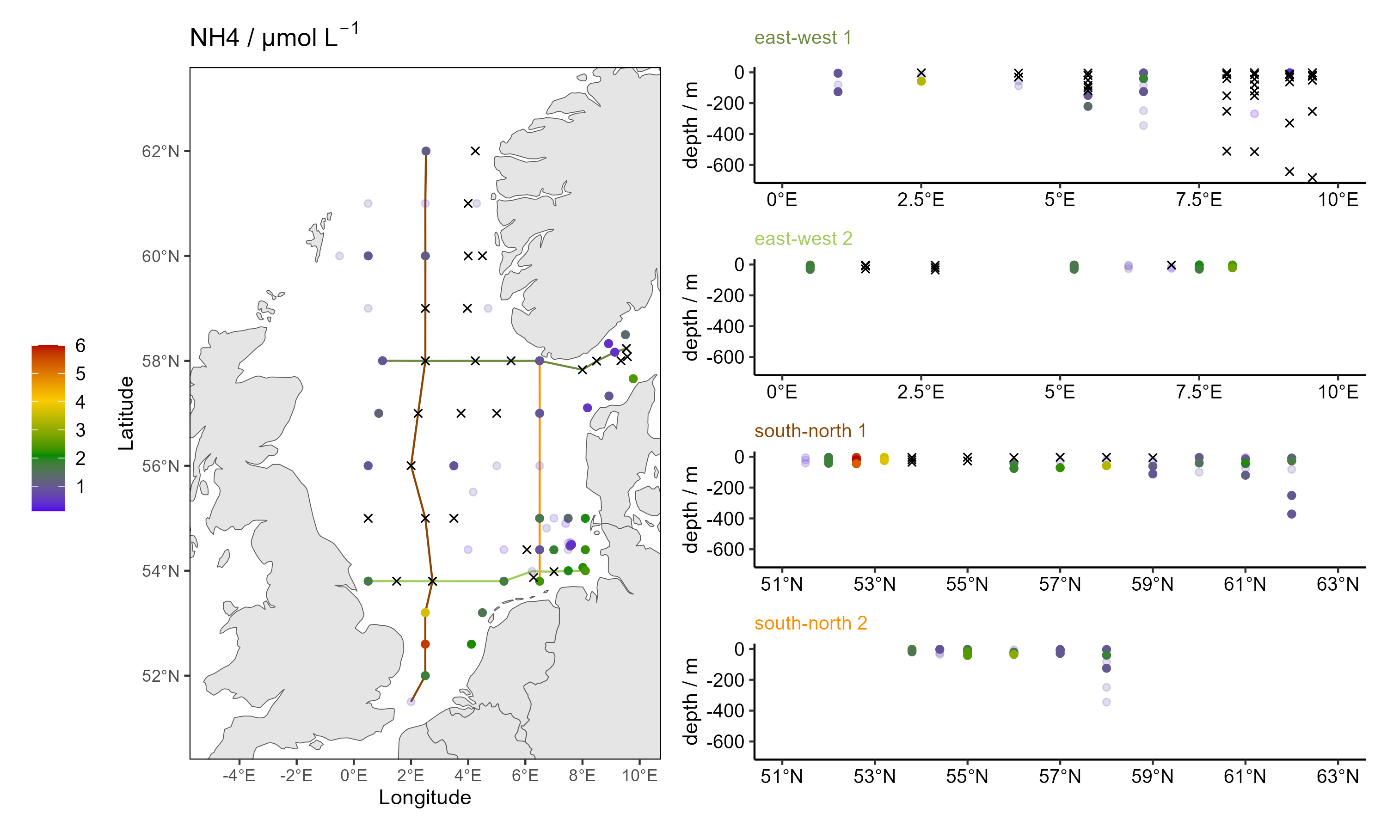


Figure S19 Surface concentrations and depth profiles of NH_4_^+^ across two south-north and two east-west transects. The northernmost transect is east-west 1 and the westernmost transect is south-north 1. x indicates that concentrations were below the LOD and faint points indicate that concentrations were between LOD and LOQ.


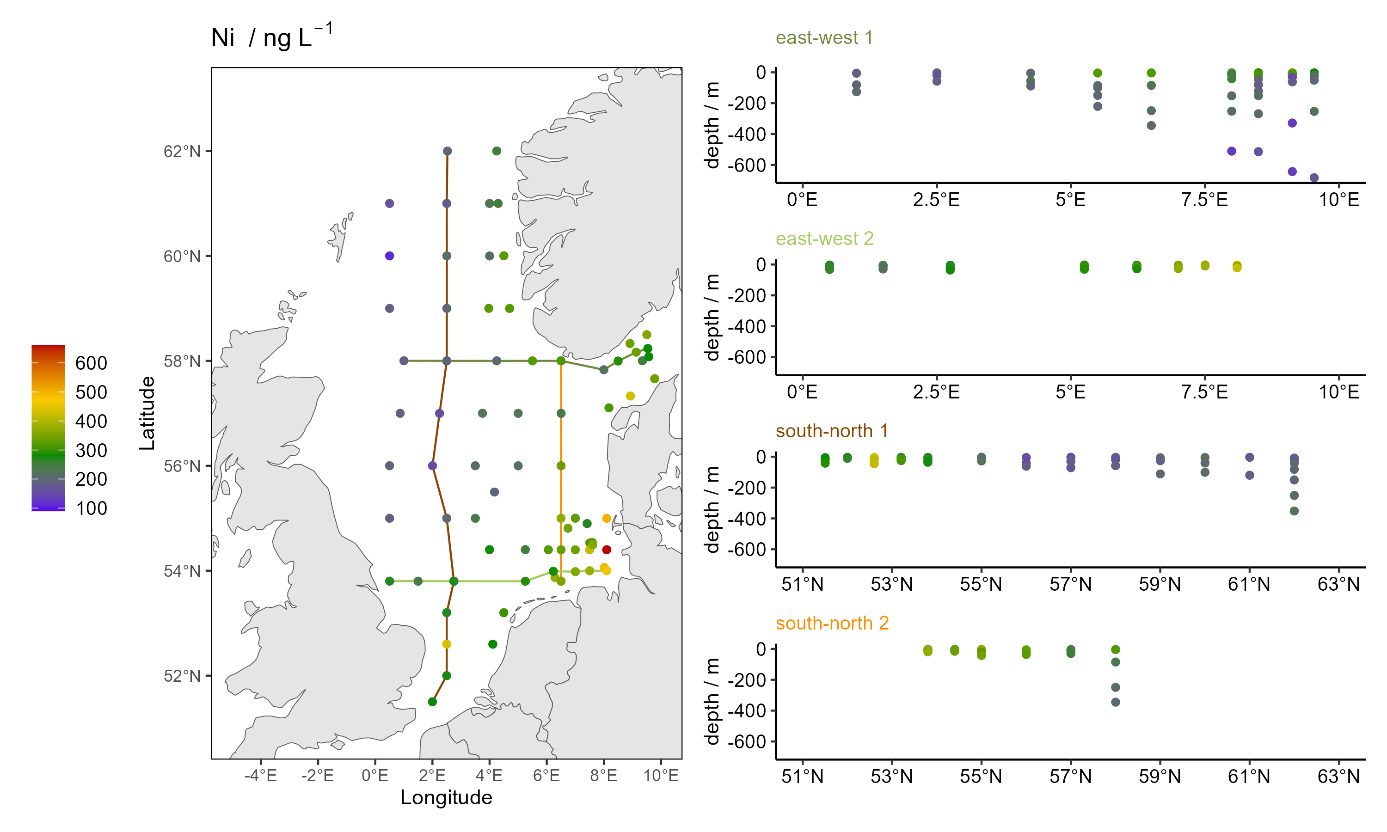


Figure S20 Surface concentrations and depth profiles of Ni across two south-north and two east-west transects. The northernmost transect is east-west 1 and the westernmost transect is south-north 1. x indicates that concentrations were below the LOD and faint points indicate that concentrations were between LOD and LOQ.


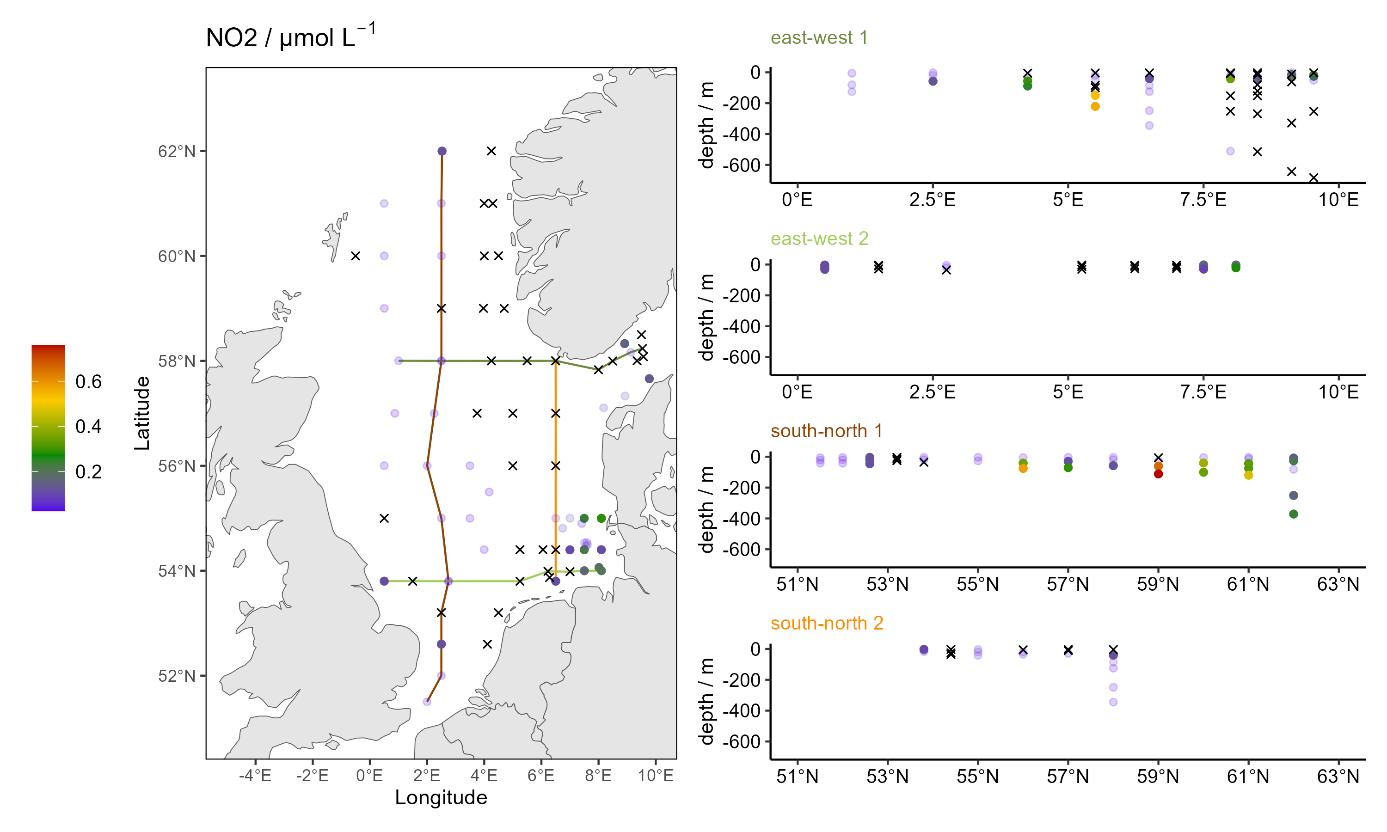


Figure S21 Surface concentrations and depth profiles of NO_2_^-^ across two south-north and two east-west transects. The northernmost transect is east-west 1 and the westernmost transect is south-north 1. x indicates that concentrations were below the LOD and faint points indicate that concentrations were between LOD and LOQ.


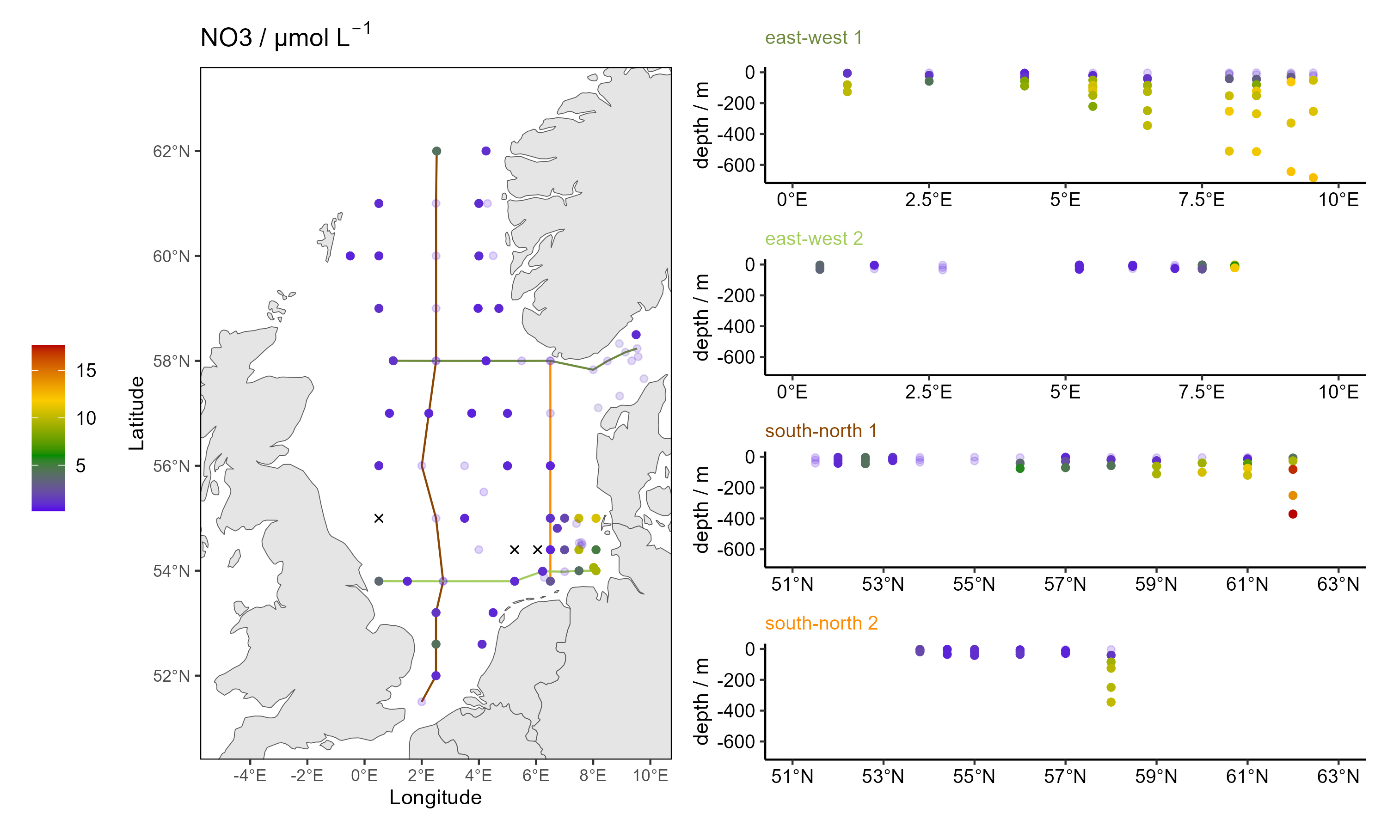


Figure S22 Surface concentrations and depth profiles of NO_3_^-^ across two south-north and two east-west transects. The northernmost transect is east-west 1 and the westernmost transect is south-north 1. x indicates that concentrations were below the LOD and faint points indicate that concentrations were between LOD and LOQ.


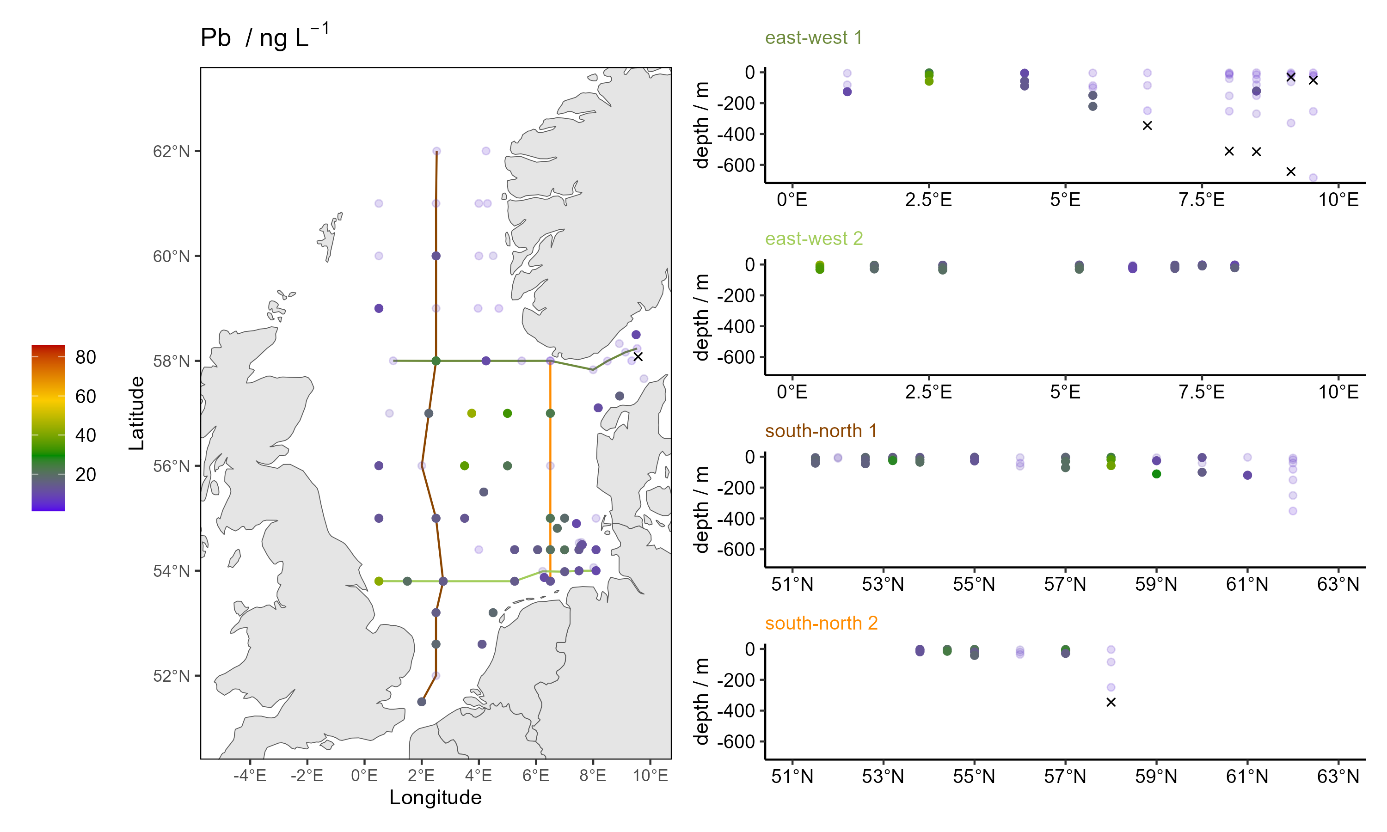


Figure S23 Surface concentrations and depth profiles of Pb across two south-north and two east-west transects. The northernmost transect is east-west 1 and the westernmost transect is south-north 1. x indicates that concentrations were below the LOD and faint points indicate that concentrations were between LOD and LOQ.


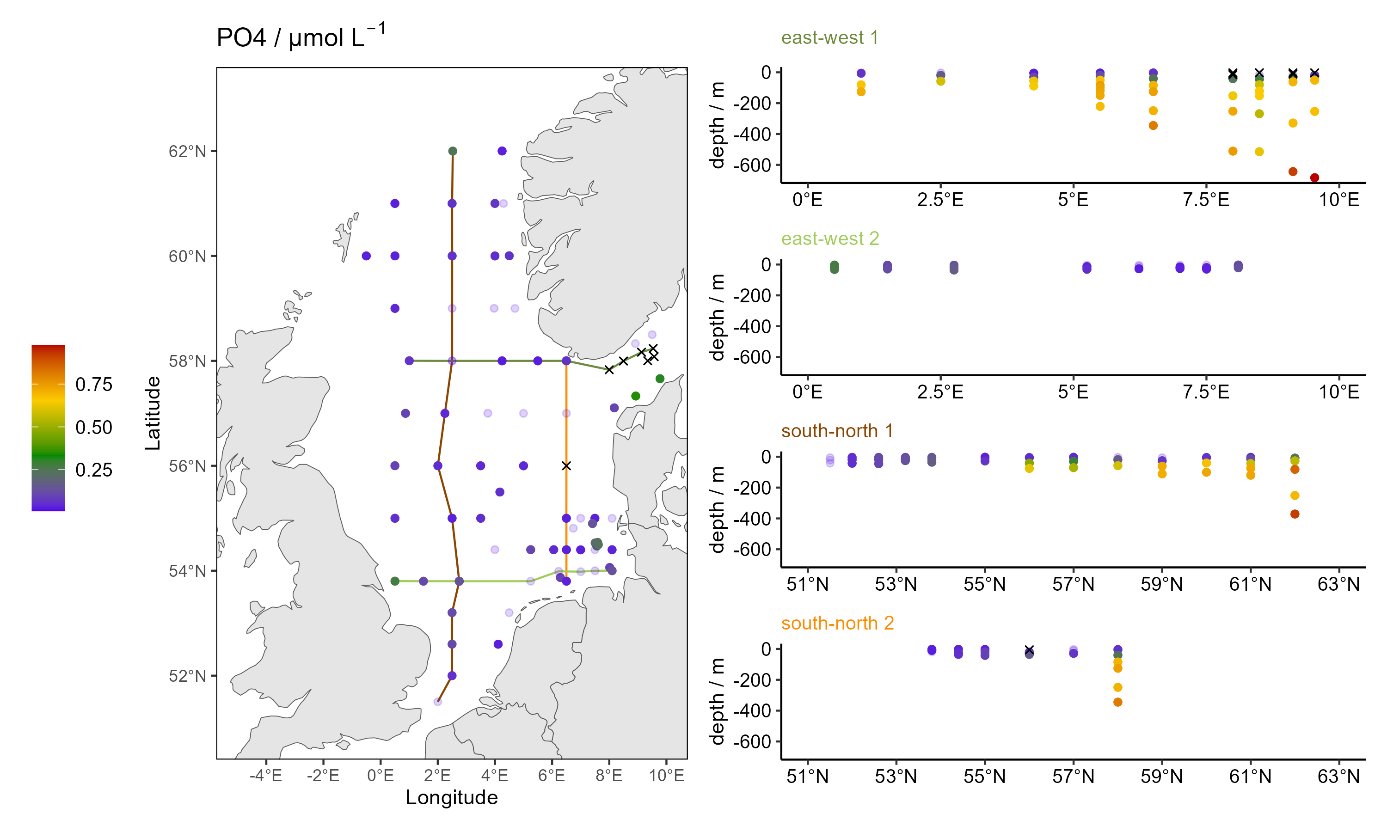


Figure S24 Surface concentrations and depth profiles of PO_4_^3-^ across two south-north and two east-west transects. The northernmost transect is east-west 1 and the westernmost transect is south-north 1. x indicates that concentrations were below the LOD and faint points indicate that concentrations were between LOD and LOQ.


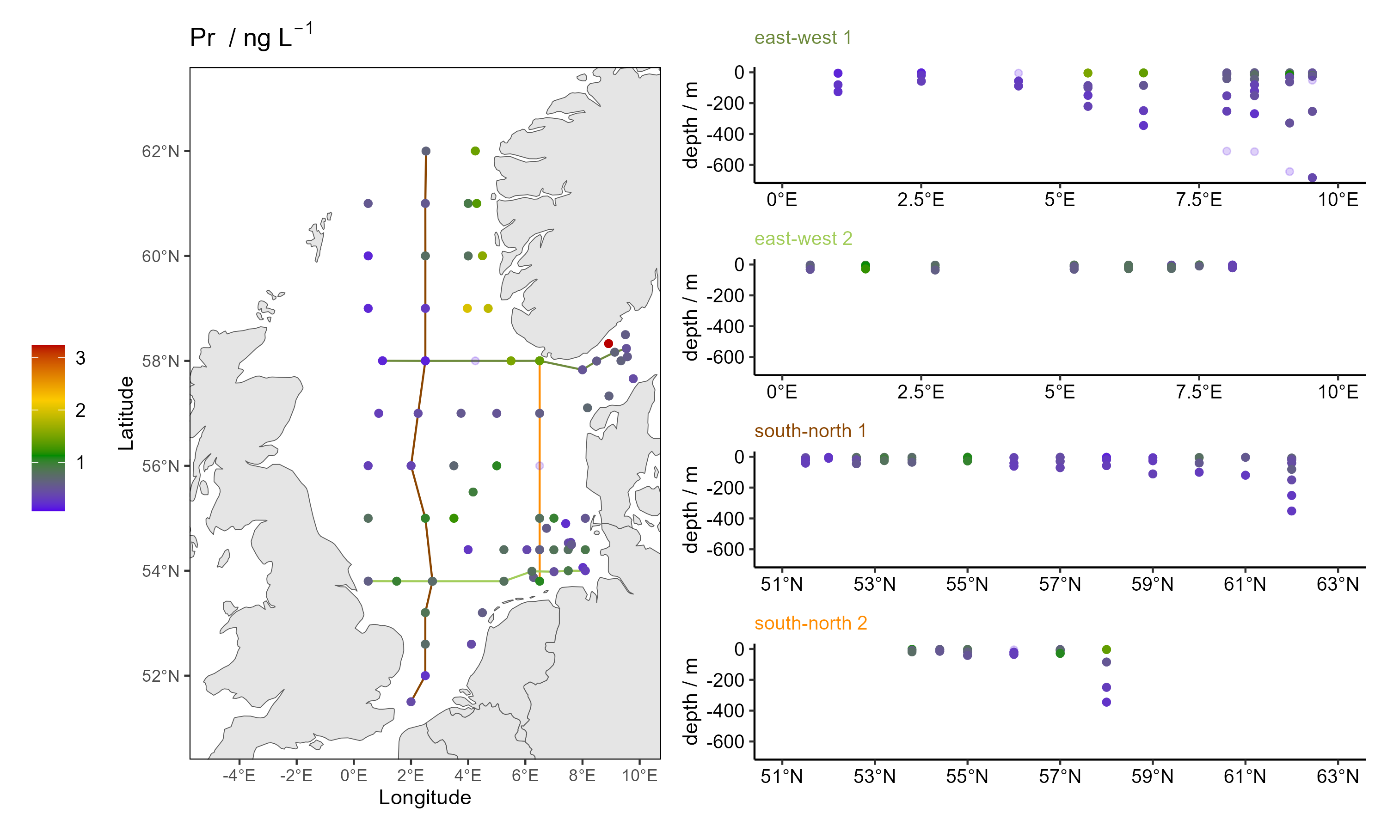


Figure S25 Surface concentrations and depth profiles of Pr across two south-north and two east-west transects. The northernmost transect is east-west 1 and the westernmost transect is south-north 1. x indicates that concentrations were below the LOD and faint points indicate that concentrations were between LOD and LOQ.


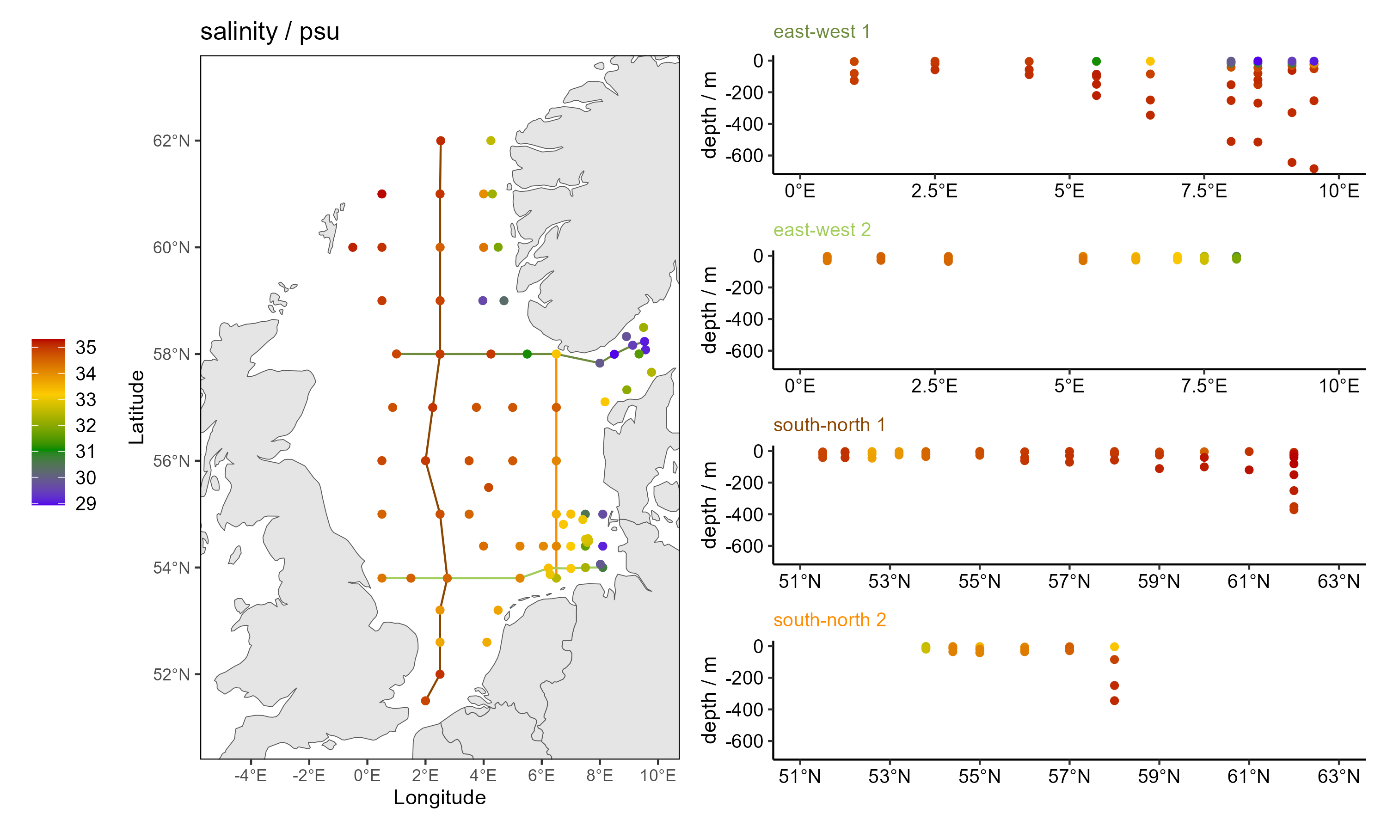


Figure S26 Surface concentrations and depth profiles of salinity across two south-north and two east-west transects. The northernmost transect is east-west 1 and the westernmost transect is south-north 1. x indicates that concentrations were below the LOD and faint points indicate that concentrations were between LOD and LOQ.


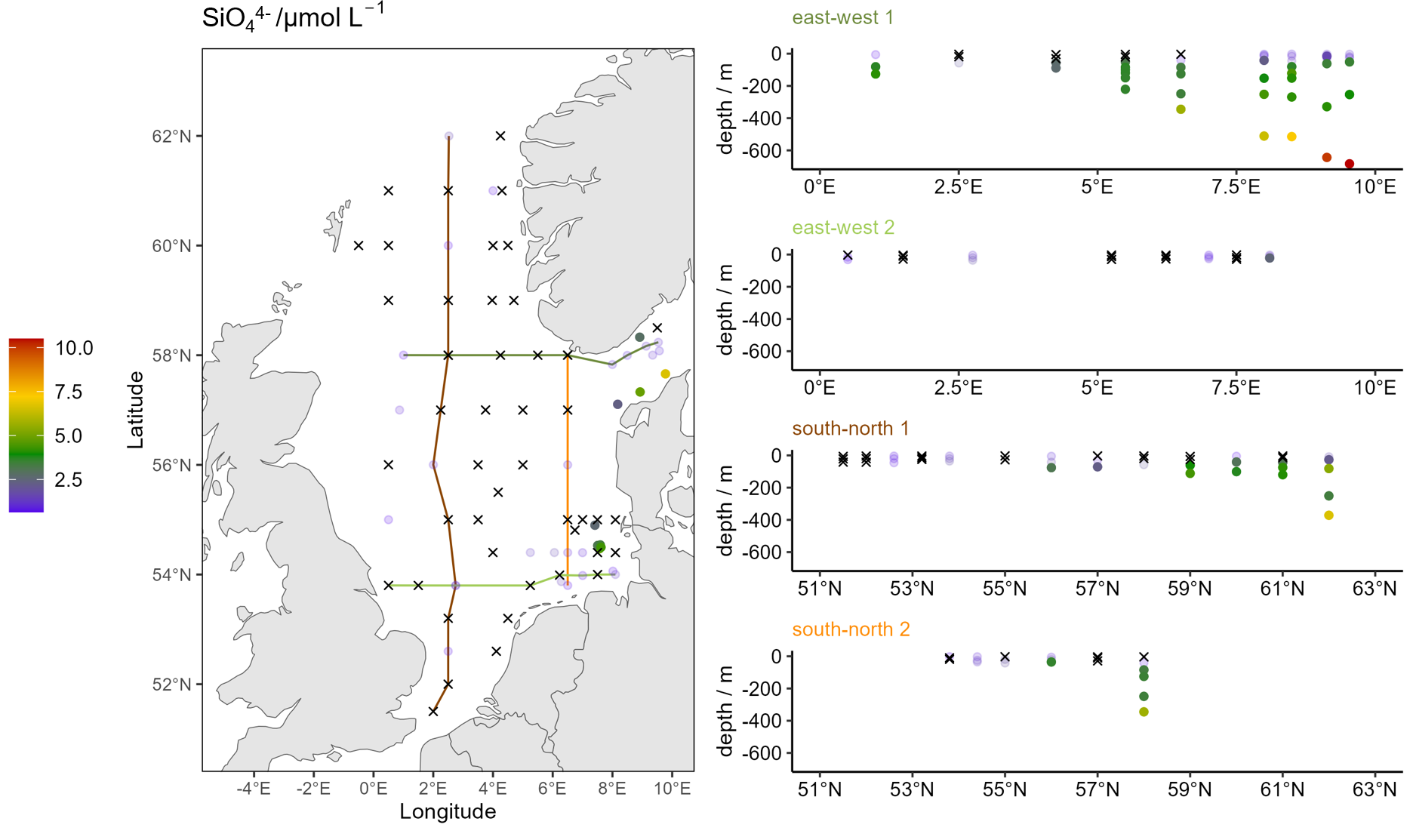


Figure S27 Surface concentrations and depth profiles of SiO_4_^4-^ across two south-north and two east-west transects. The northernmost transect is east-west 1 and the westernmost transect is south-north 1. x indicates that concentrations were below the LOD and faint points indicate that concentrations were between LOD and LOQ.


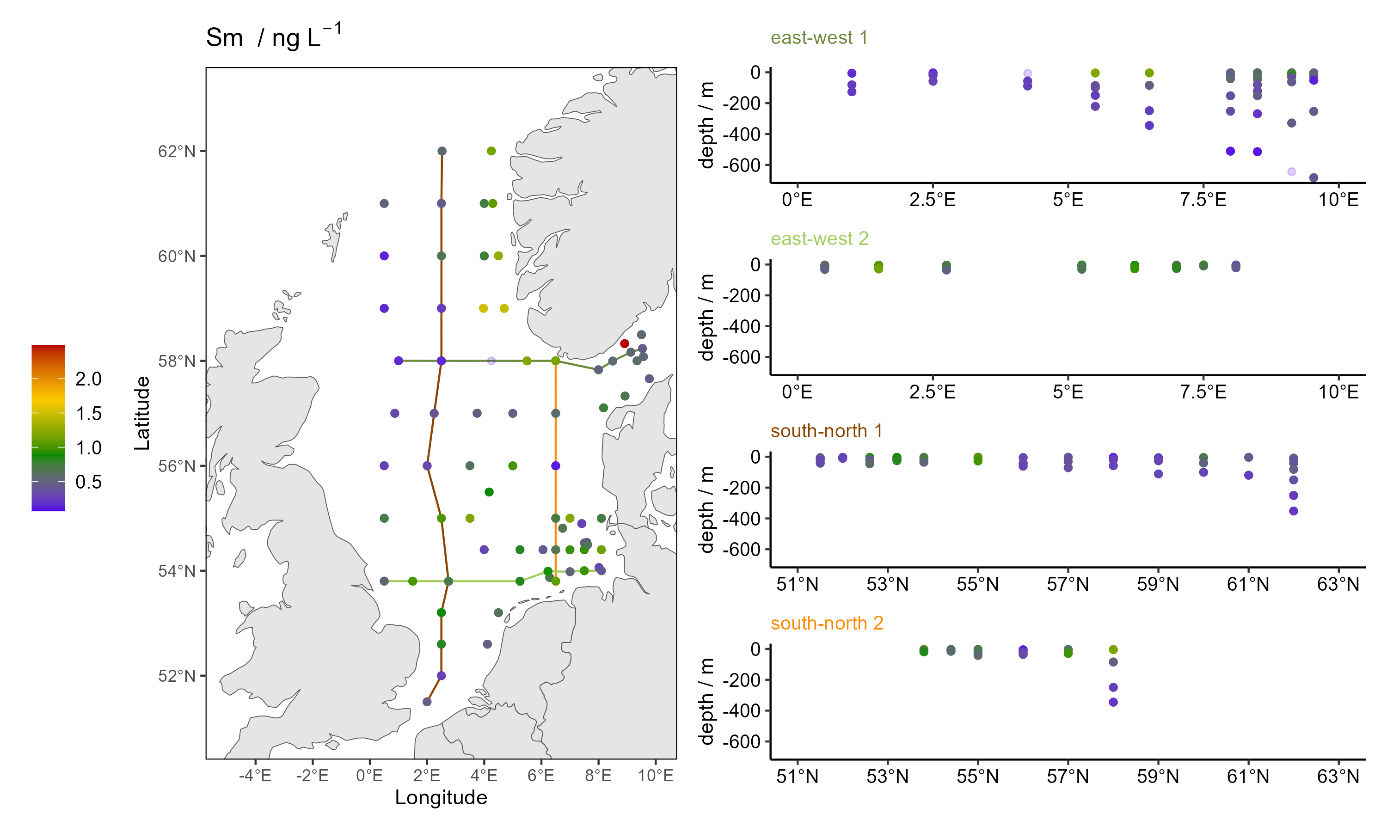


Figure S28 Surface concentrations and depth profiles of Sm across two south-north and two east-west transects. The northernmost transect is east-west 1 and the westernmost transect is south-north 1. x indicates that concentrations were below the LOD and faint points indicate that concentrations were between LOD and LOQ.


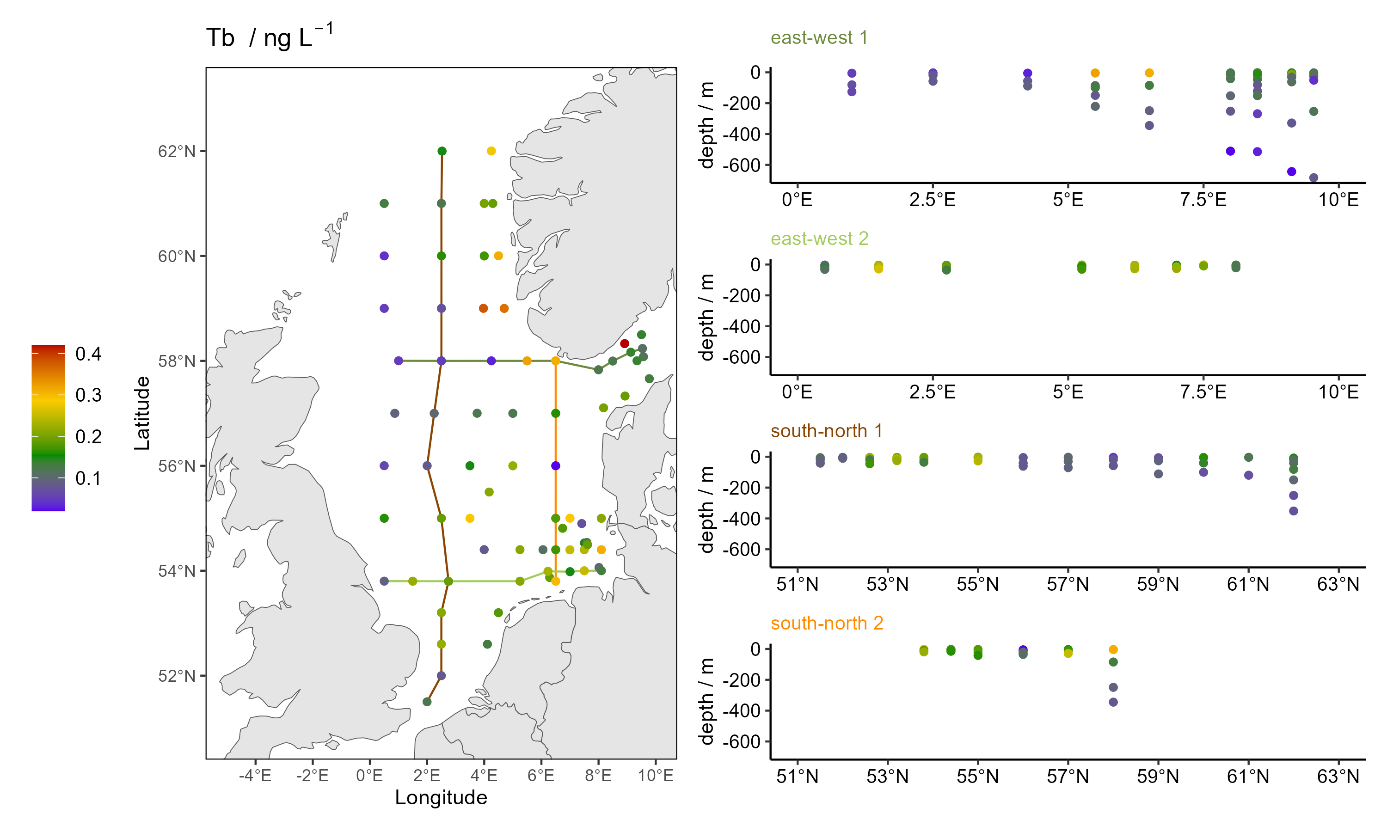


Figure S29 Surface concentrations and depth profiles of Tb across two south-north and two east-west transects. The northernmost transect is east-west 1 and the westernmost transect is south-north 1. x indicates that concentrations were below the LOD and faint points indicate that concentrations were between LOD and LOQ.


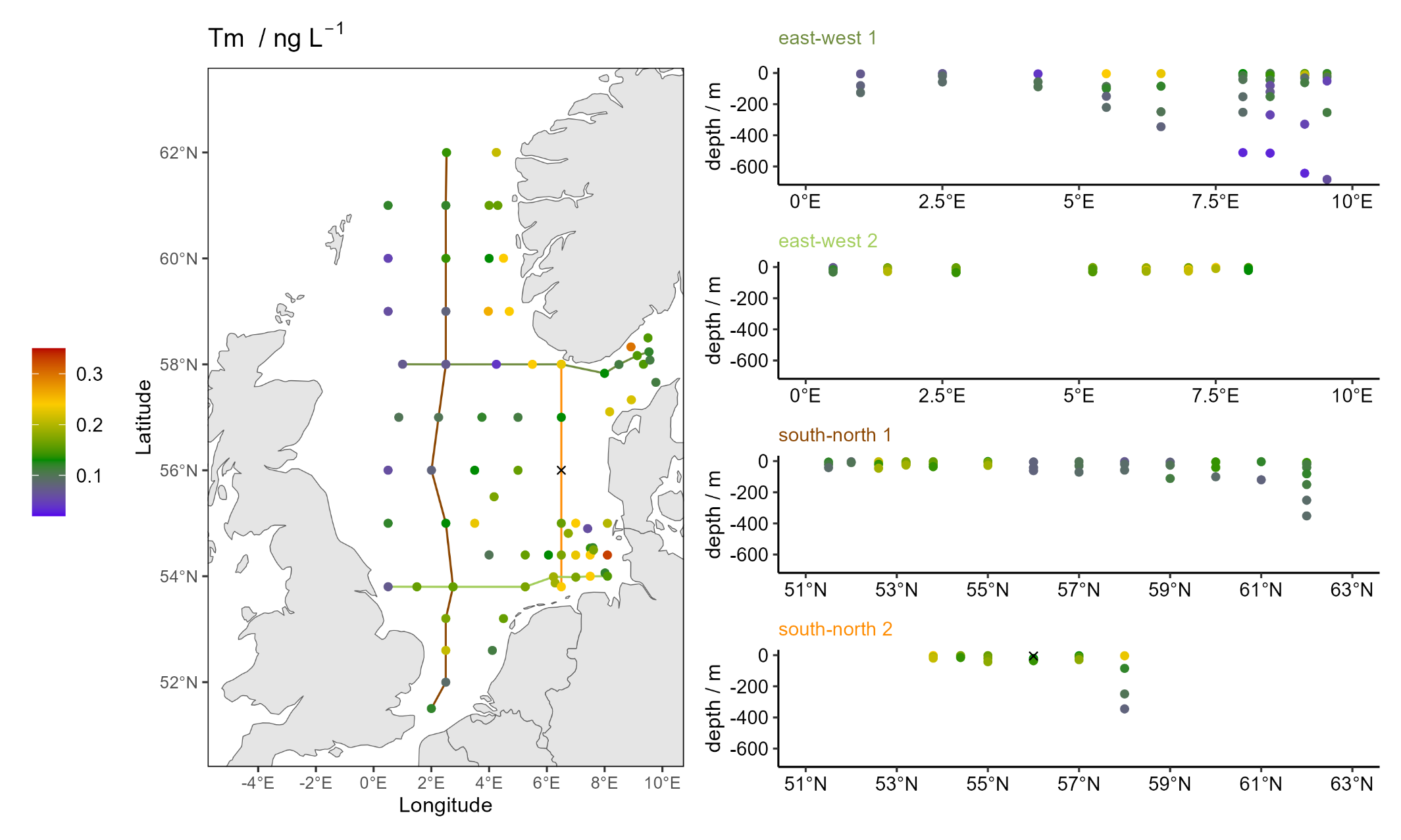


Figure S30 Surface concentrations and depth profiles of Tm across two south-north and two east-west transects. The northernmost transect is east-west 1 and the westernmost transect is south-north 1. x indicates that concentrations were below the LOD and faint points indicate that concentrations were between LOD and LOQ.


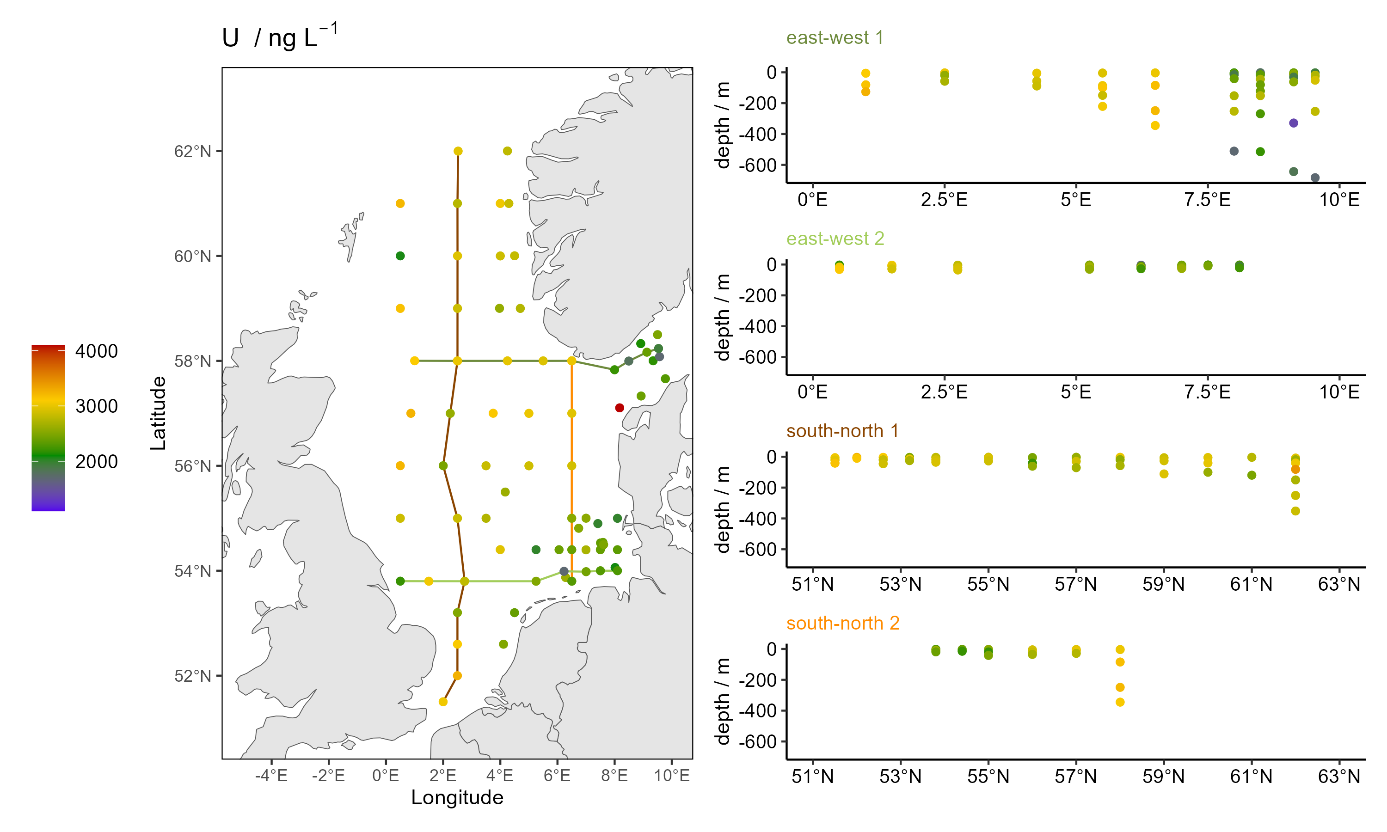


Figure S31 Surface concentrations and depth profiles of U across two south-north and two east-west transects. The northernmost transect is east-west 1 and the westernmost transect is south-north 1. x indicates that concentrations were below the LOD and faint points indicate that concentrations were between LOD and LOQ.


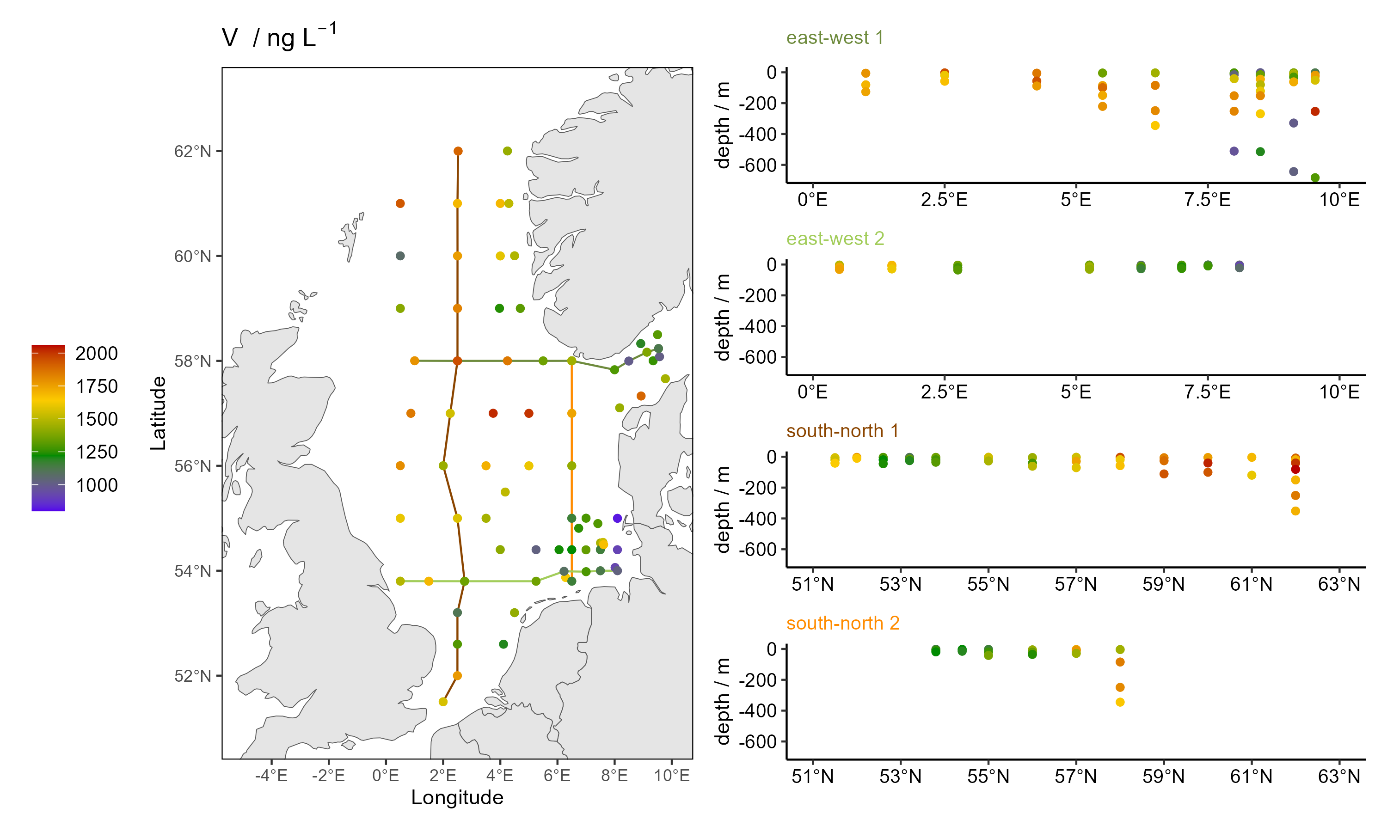


Figure S32 Surface concentrations and depth profiles of V across two south-north and two east-west transects. The northernmost transect is east-west 1 and the westernmost transect is south-north 1. x indicates that concentrations were below the LOD and faint points indicate that concentrations were between LOD and LOQ.


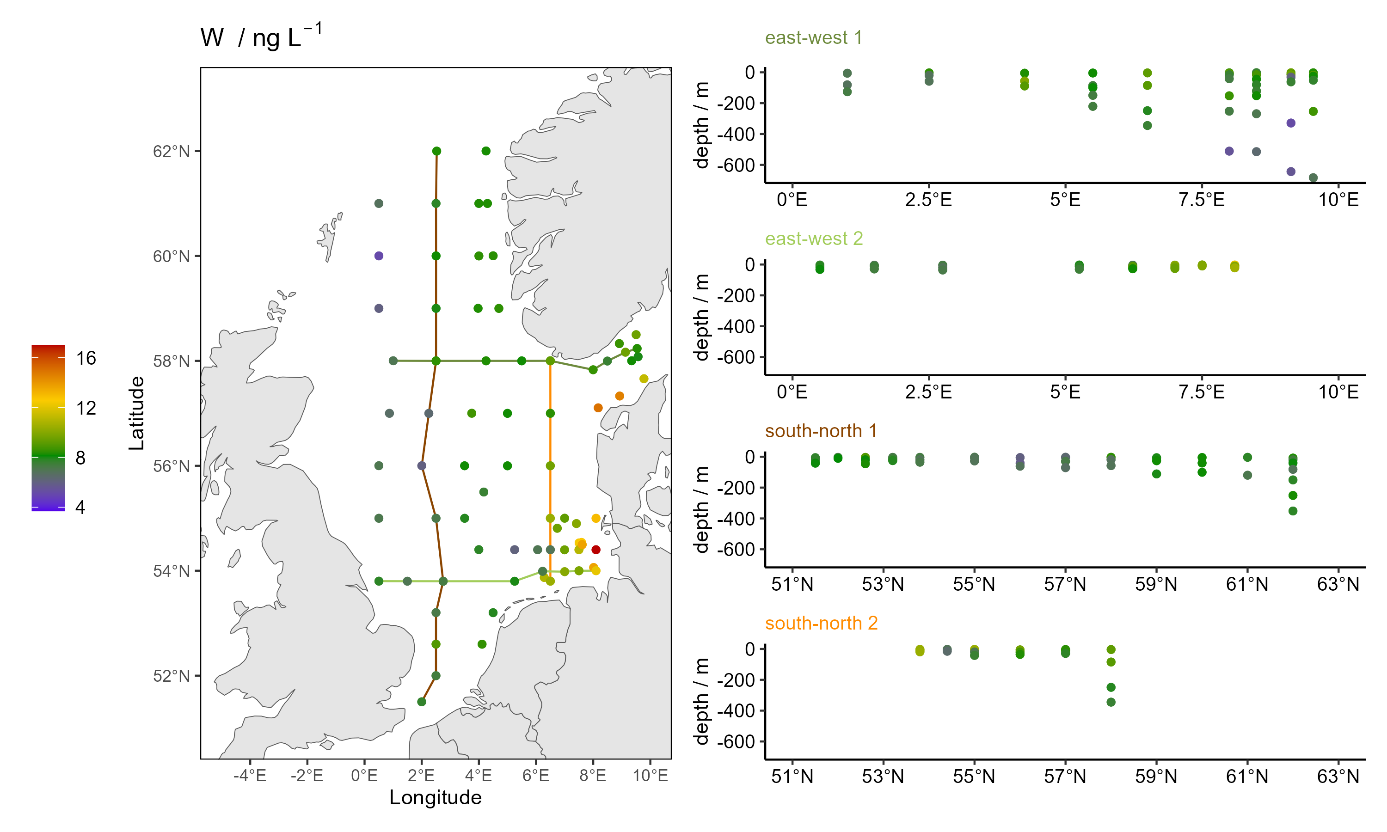


Figure S33 Surface concentrations and depth profiles of W across two south-north and two east-west transects. The northernmost transect is east-west 1 and the westernmost transect is south-north 1. x indicates that concentrations were below the LOD and faint points indicate that concentrations were between LOD and LOQ.


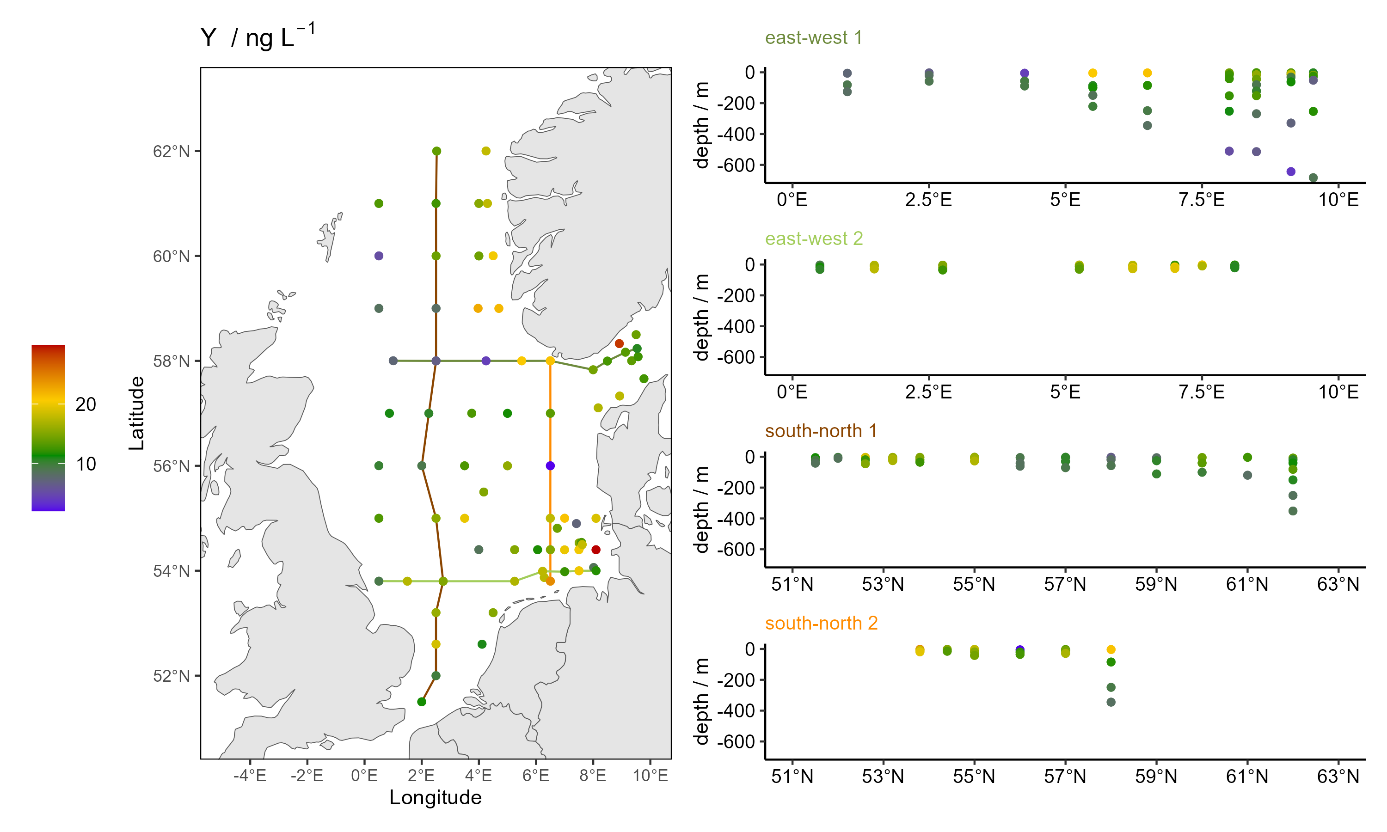


Figure S34 Surface concentrations and depth profiles of Y across two south-north and two east-west transects. The northernmost transect is east-west 1 and the westernmost transect is south-north 1. x indicates that concentrations were below the LOD and faint points indicate that concentrations were between LOD and LOQ.


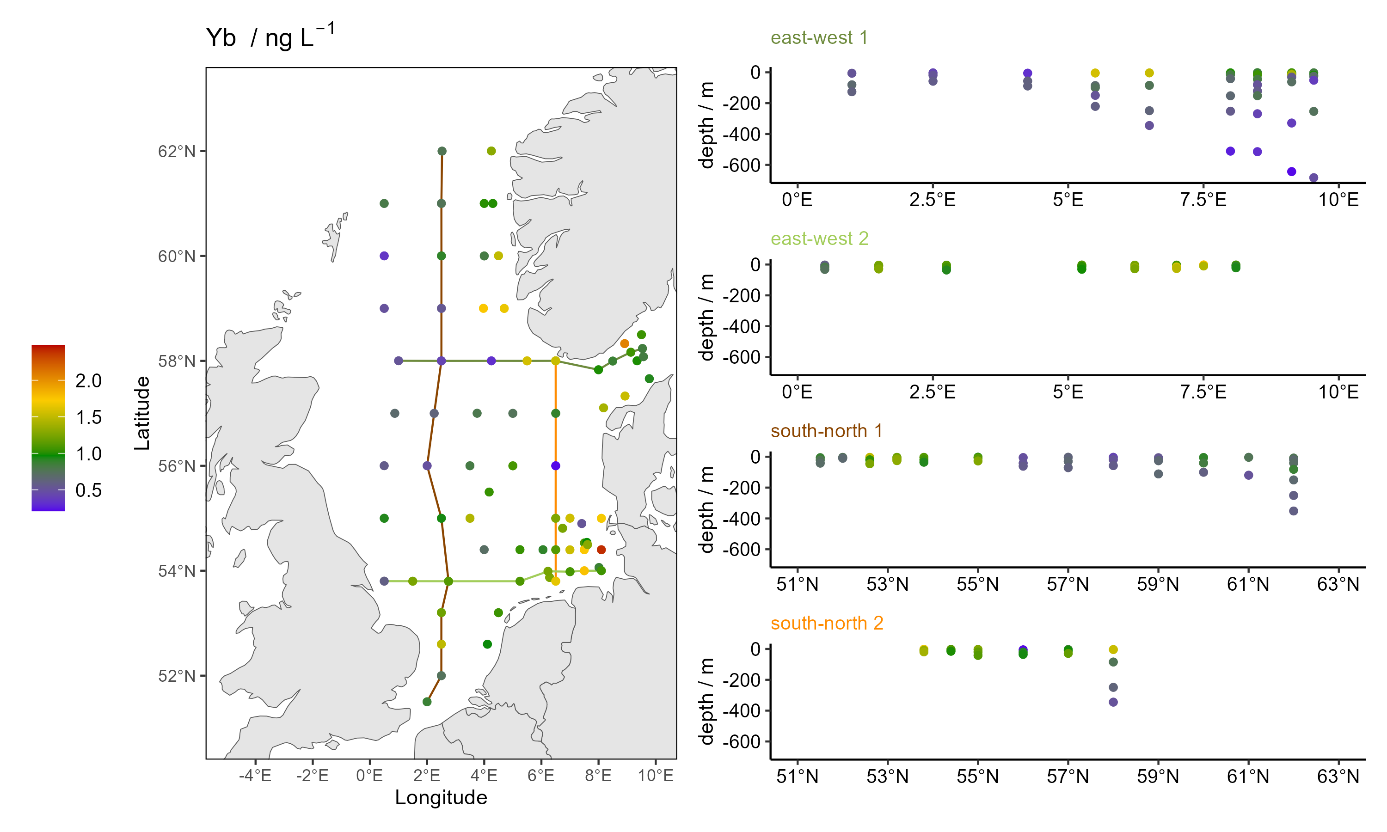


Figure S35 Surface concentrations and depth profiles of Yb across two south-north and two east-west transects. The northernmost transect is east-west 1 and the westernmost transect is south-north 1. x indicates that concentrations were below the LOD and faint points indicate that concentrations were between LOD and LOQ.


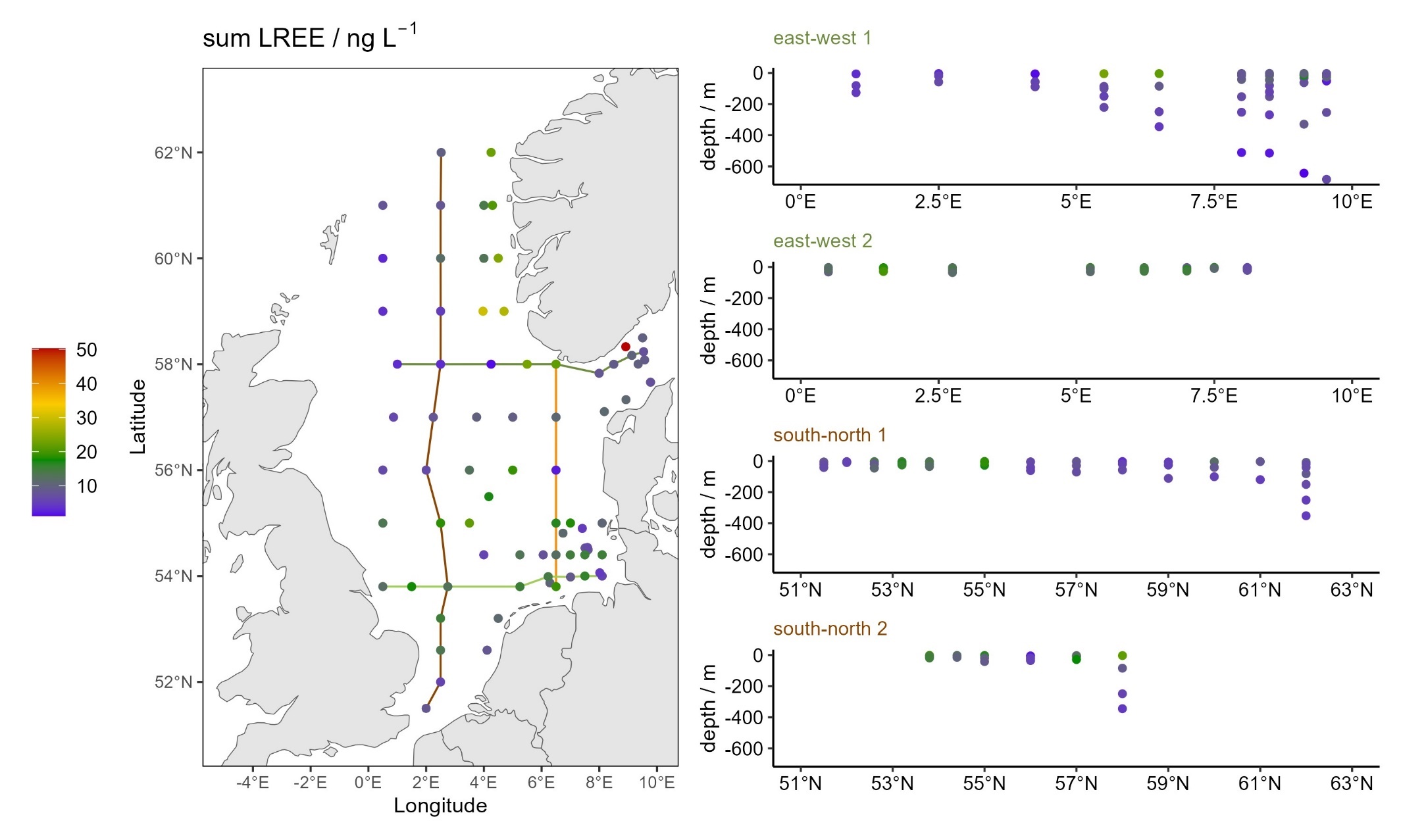


Figure S36 Surface concentrations and depth profiles of the sum of LREE across two south-north and two east-west transects. The northernmost transect is east-west 1 and the westernmost transect is south-north 1.


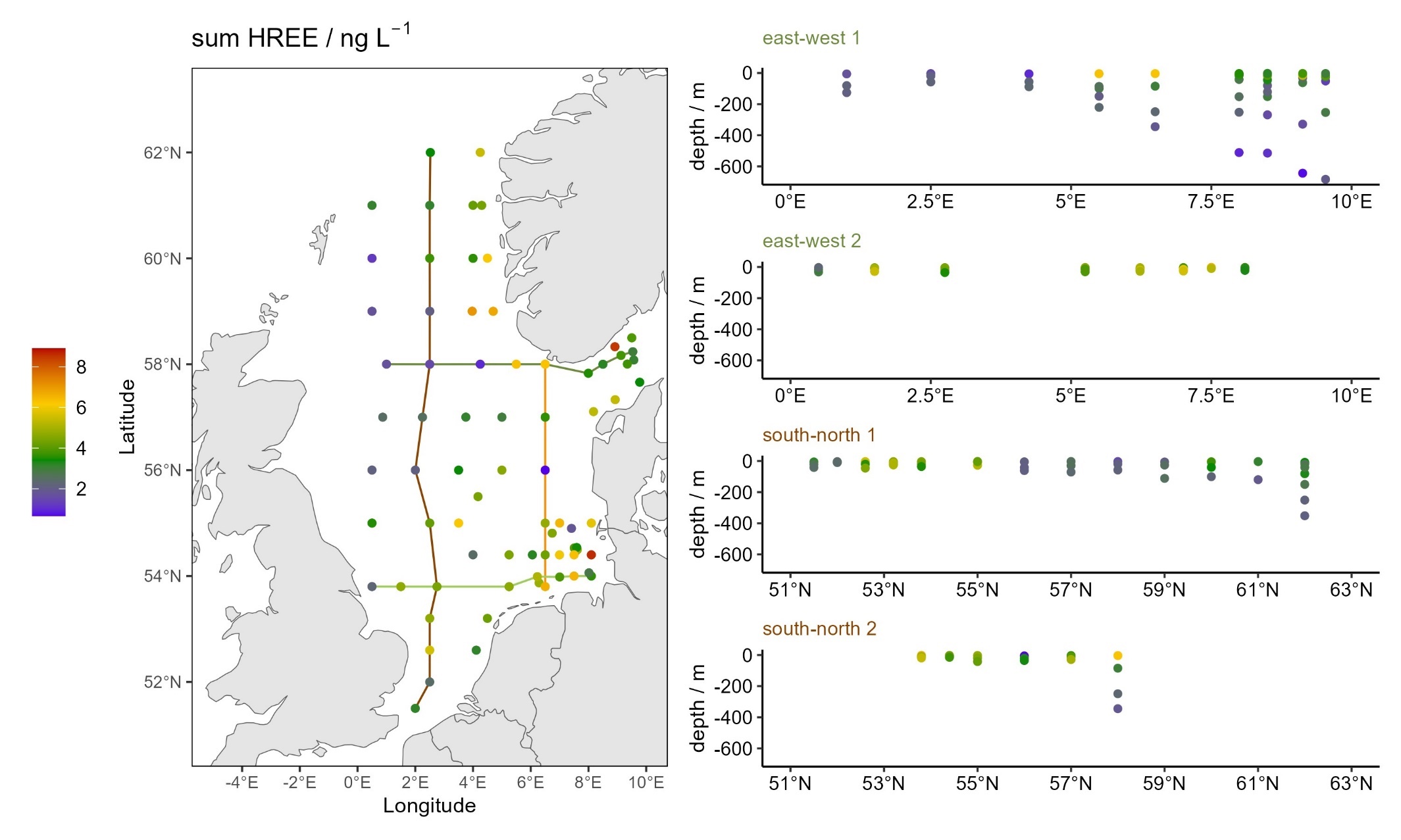


Figure S37 Surface concentrations and depth profiles of the sum of HREE across two south-north and two east-west transects. The northernmost transect is east-west 1 and the westernmost transect is south-north 1.


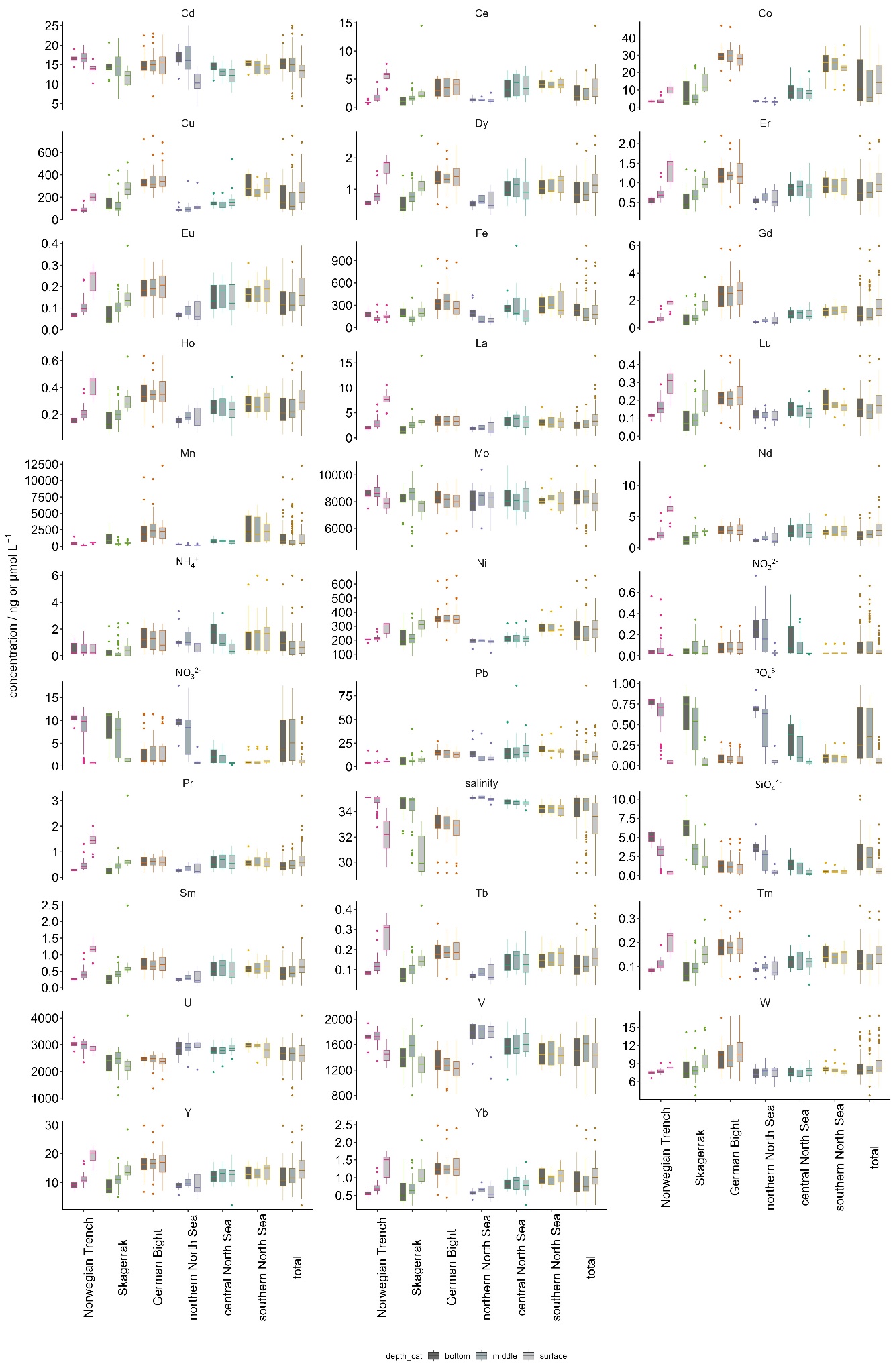


*Figure S38 Boxplot of salinity and all analytes in the North Sea (total) and six regions of the North Sea. The three boxplots per region indicate bottom (dark grey), intermediate depth (medium grey) and surface (light grey) samples.*


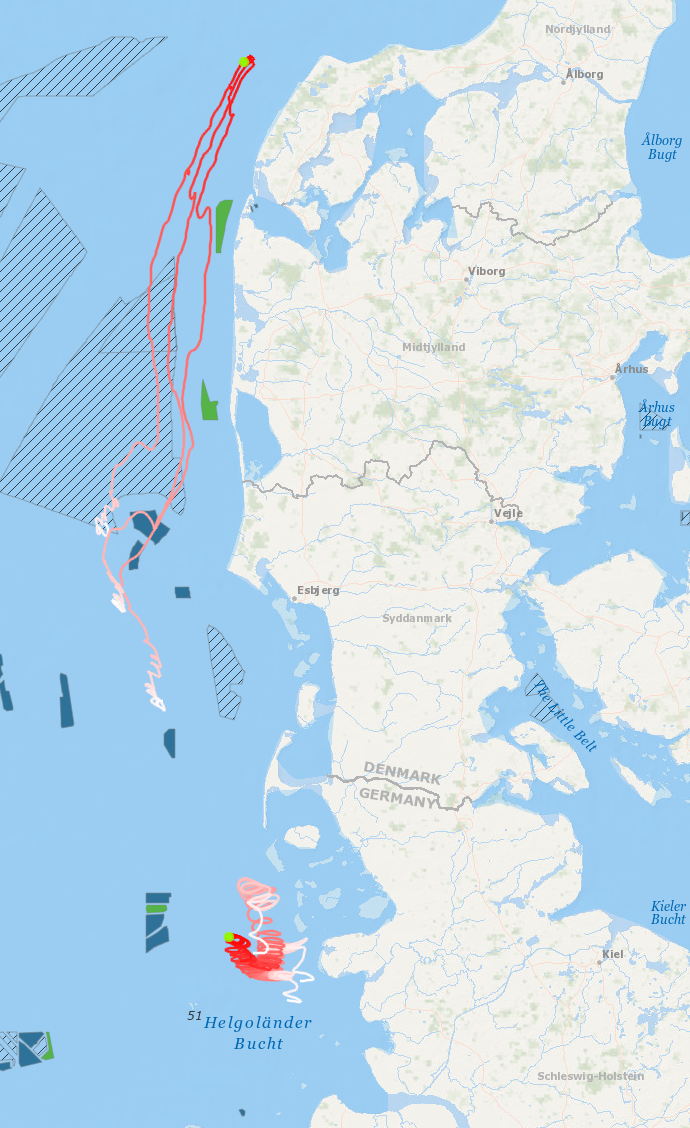
Figure S39 Drift results for AL557 st.4 and HE586 st.26 2 weeks back with vertical mean marine currents and top layer marine currents without and with (factor 0.6) wind drag.


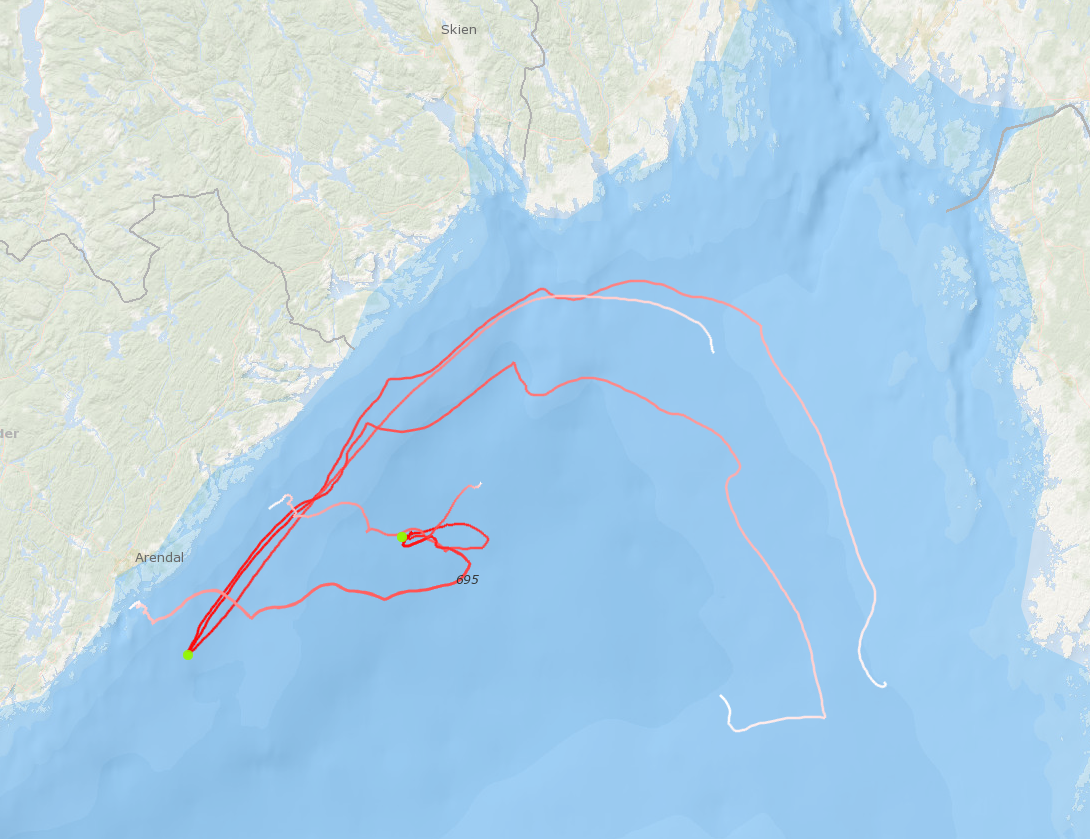


Figure S40 Drift results for HE586 st.10 (left) and AL557 st.65 (right) from two weeks before sampling (white) to sampling time (dark red) for three different model settings: vertical mean marine currents, top layer marine currents without and with (factor 0.6) wind drag.


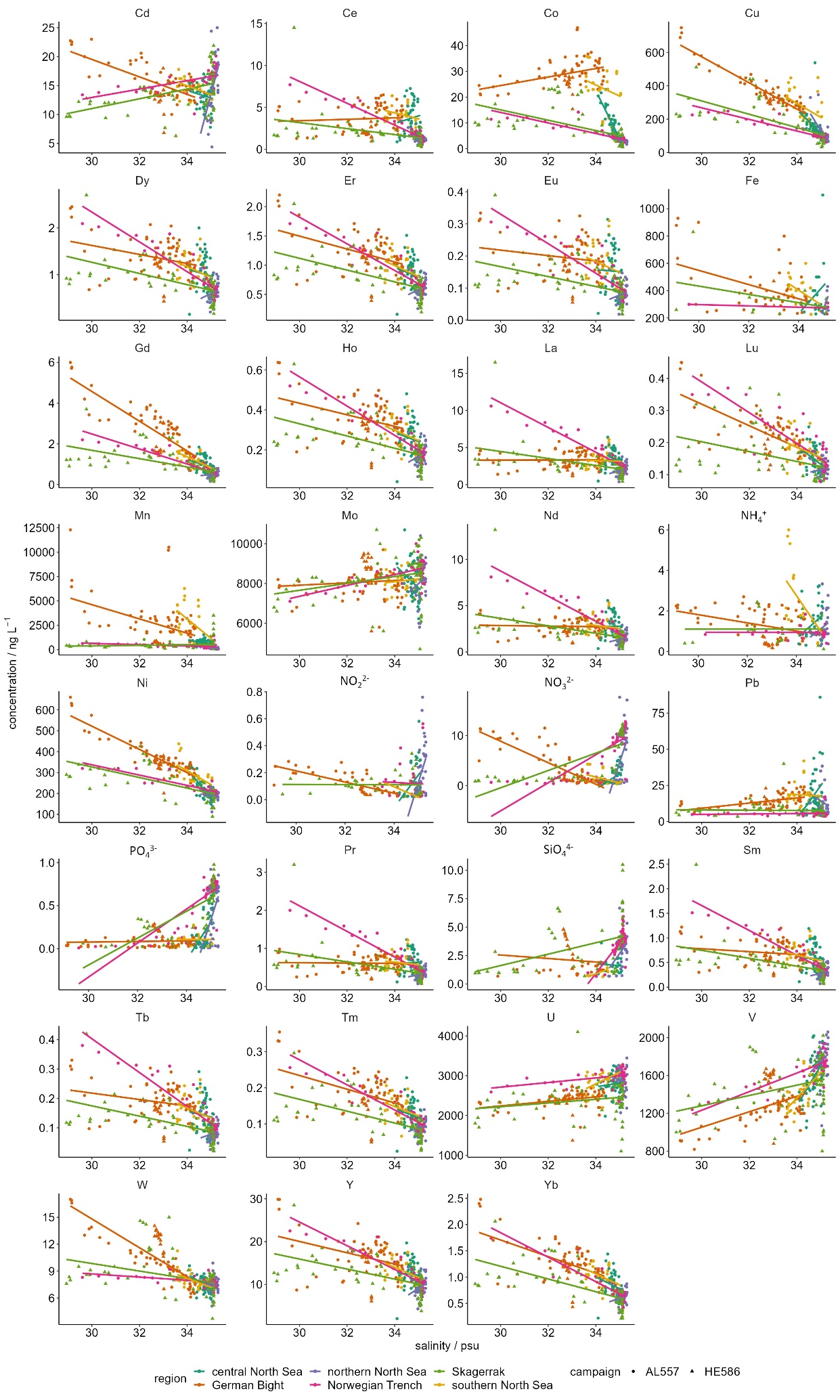


Figure S41 Correlation plot of all analytes with salinity for the six regions (see legend) and campaigns AL557 (dot) and HE586 (triangle). Regression coefficients are given in Table S5.

Table S5 Regression coefficients for all analytes with salinity for the different regions from Figure S41.

| analyte | northern North Sea_b0 | | | central North Sea_b0 | | | southern North Sea_b0 | | |
| --- | --- | --- | --- | --- | --- | --- | --- | --- | --- |
|  | intercept | slope | R^2^ | intercept | slope | R^2^ | intercept | slope | R^2^ |
| NO_3_^-^ | -504.97 | 14.56 | 0.19 | -104.06 | 3.04 | 0.26 | 42.29 | -1.19 | 0.18 |
| NO_2_^-^ | -20.55 | 0.59 | 0.22 | -8.36 | 0.24 | 0.20 | 2.70 | -0.08 | 0.68 |
| SiO_4_^4-^ | -101.07 | 2.97 | 0.12 | 8.33 | -0.19 | 0.01 | -24.52 | 0.75 | 0.42 |
| PO_4_^3+^ | -30.86 | 0.89 | 0.19 | -15.23 | 0.44 | 0.27 | 0.14 | 0.00 | 0.00 |
| NH_4_^+^ | -32.40 | 0.96 | 0.04 | -35.71 | 1.07 | 0.11 | 63.06 | -1.77 | 0.35 |
| V | -7279.27 | 257.88 | 0.04 | -13191.35 | 425.63 | 0.21 | -7743.96 | 267.86 | 0.41 |
| Mn | 11983.89 | -335.63 | 0.17 | 13165.65 | -358.62 | 0.08 | 71004.85 | -1995.64 | 0.23 |
| Fe | -17828.96 | 516.93 | 0.28 | -6735.88 | 204.92 | 0.07 | 4448.47 | -118.96 | 0.21 |
| Co | 91.26 | -2.51 | 0.28 | 658.32 | -18.70 | 0.70 | 170.26 | -4.28 | 0.07 |
| Ni | 654.02 | -13.20 | 0.01 | 5254.55 | -145.13 | 0.62 | 2544.42 | -65.75 | 0.36 |
| Cu | 8353.77 | -234.93 | 0.39 | 5111.29 | -142.74 | 0.22 | 3148.62 | -83.92 | 0.19 |
| Mo | -28948.78 | 1055.56 | 0.02 | 49218.36 | -1184.37 | 0.09 | 3499.80 | 135.03 | 0.01 |
| Cd | -533.23 | 15.62 | 0.28 | 58.46 | -1.30 | 0.02 | 67.22 | -1.54 | 0.21 |
| W | 13.92 | -0.18 | 0.00 | 61.91 | -1.57 | 0.19 | 42.29 | -1.00 | 0.26 |
| Pb | 399.47 | -11.00 | 0.03 | -780.46 | 23.01 | 0.13 | 51.20 | -0.96 | 0.00 |
| U | 10491.86 | -217.12 | 0.01 | -818.59 | 103.96 | 0.01 | -7193.87 | 293.79 | 0.27 |
| Y | -119.08 | 3.66 | 0.06 | 35.20 | -0.66 | 0.00 | 94.46 | -2.37 | 0.14 |
| La | -29.97 | 0.91 | 0.03 | -3.48 | 0.20 | 0.00 | 5.33 | -0.06 | 0.00 |
| Ce | 29.62 | -0.81 | 0.08 | -27.29 | 0.89 | 0.01 | 28.59 | -0.71 | 0.09 |
| Pr | -4.27 | 0.13 | 0.02 | -0.74 | 0.04 | 0.00 | 2.78 | -0.06 | 0.01 |
| Nd | -21.23 | 0.65 | 0.02 | 0.05 | 0.08 | 0.00 | 13.47 | -0.31 | 0.02 |
| Sm | -3.88 | 0.12 | 0.02 | 1.12 | -0.02 | 0.00 | 4.53 | -0.11 | 0.05 |
| Eu | -0.99 | 0.03 | 0.02 | 0.37 | -0.01 | 0.00 | 1.32 | -0.03 | 0.06 |
| Gd | -4.05 | 0.13 | 0.01 | 12.95 | -0.35 | 0.05 | 19.42 | -0.53 | 0.42 |
| Tb | -0.96 | 0.03 | 0.02 | 0.81 | -0.02 | 0.01 | 1.33 | -0.03 | 0.09 |
| Dy | -8.37 | 0.26 | 0.04 | 6.22 | -0.15 | 0.01 | 8.80 | -0.22 | 0.10 |
| Ho | -2.22 | 0.07 | 0.04 | 1.85 | -0.05 | 0.02 | 2.04 | -0.05 | 0.09 |
| Er | -6.09 | 0.19 | 0.03 | 6.24 | -0.16 | 0.02 | 7.32 | -0.19 | 0.12 |
| Tm | -0.73 | 0.02 | 0.02 | 0.98 | -0.02 | 0.02 | 1.02 | -0.03 | 0.11 |
| Yb | -4.56 | 0.15 | 0.03 | 8.67 | -0.23 | 0.05 | 8.31 | -0.21 | 0.19 |
| Lu | -0.47 | 0.02 | 0.01 | 2.12 | -0.06 | 0.10 | 1.25 | -0.03 | 0.11 |

Table S5 continued.

| analyte | German Bight_b0 | | | Skagerrak_b0 | | | Norwegian Trench_b0 | | |
| --- | --- | --- | --- | --- | --- | --- | --- | --- | --- |
|  | intercept | slope | R^2^ | intercept | slope | R^2^ | intercept | slope | R^2^ |
| NO_3_^-^ | 73.47 | -2.15 | 0.70 | -52.43 | 1.73 | 0.54 | -89.33 | 2.81 | 0.58 |
| NO_2_^-^ | 1.46 | -0.04 | 0.54 | 0.13 | 0.00 | 0.00 | 0.47 | -0.01 | 0.00 |
| SiO_4_^4-^ | 7.17 | -0.15 | 0.01 | -13.42 | 0.50 | 0.20 | -91.38 | 2.72 | 0.60 |
| PO_4_^3+^ | -0.02 | 0.00 | 0.00 | -4.87 | 0.16 | 0.50 | -6.25 | 0.20 | 0.66 |
| NH_4_^+^ | 7.87 | -0.20 | 0.13 | 1.09 | 0.00 | 0.00 | 0.98 | 0.00 | 0.00 |
| V | -1402.11 | 81.85 | 0.24 | -351.31 | 54.39 | 0.14 | -1716.85 | 98.33 | 0.70 |
| Mn | 26553.83 | -731.43 | 0.17 | -493.89 | 29.27 | 0.01 | 2626.85 | -66.24 | 0.09 |
| Fe | 2105.90 | -51.92 | 0.20 | 1346.02 | -30.42 | 0.15 | 439.97 | -4.74 | 0.14 |
| Co | -25.21 | 1.66 | 0.16 | 75.48 | -2.01 | 0.33 | 75.37 | -2.04 | 0.73 |
| Ni | 2190.17 | -55.60 | 0.79 | 1086.69 | -25.32 | 0.45 | 1076.72 | -24.65 | 0.73 |
| Cu | 2924.59 | -78.34 | 0.86 | 1509.95 | -40.01 | 0.52 | 1310.48 | -34.74 | 0.82 |
| Mo | 6143.24 | 59.01 | 0.01 | 2459.45 | 173.27 | 0.09 | -866.29 | 274.00 | 0.37 |
| Cd | 64.79 | -1.51 | 0.41 | -14.30 | 0.84 | 0.23 | -8.85 | 0.73 | 0.32 |
| W | 63.03 | -1.61 | 0.67 | 22.50 | -0.42 | 0.15 | 13.96 | -0.18 | 0.20 |
| Pb | -44.22 | 1.78 | 0.22 | 10.59 | -0.08 | 0.00 | 0.43 | 0.15 | 0.01 |
| U | 523.29 | 57.20 | 0.10 | 815.22 | 46.80 | 0.04 | 966.79 | 58.16 | 0.15 |
| Y | 59.63 | -1.32 | 0.14 | 51.01 | -1.17 | 0.34 | 107.53 | -2.77 | 0.81 |
| La | 3.11 | 0.01 | 0.00 | 18.57 | -0.47 | 0.22 | 61.06 | -1.67 | 0.92 |
| Ce | 0.85 | 0.09 | 0.00 | 13.86 | -0.36 | 0.16 | 47.17 | -1.30 | 0.90 |
| Pr | 0.74 | 0.00 | 0.00 | 3.75 | -0.10 | 0.24 | 12.17 | -0.34 | 0.92 |
| Nd | 3.82 | -0.03 | 0.00 | 15.70 | -0.40 | 0.24 | 49.55 | -1.36 | 0.91 |
| Sm | 1.67 | -0.03 | 0.03 | 3.16 | -0.08 | 0.26 | 9.24 | -0.25 | 0.88 |
| Eu | 0.47 | -0.01 | 0.03 | 0.63 | -0.02 | 0.28 | 1.71 | -0.05 | 0.78 |
| Gd | 26.48 | -0.73 | 0.65 | 7.82 | -0.20 | 0.38 | 13.54 | -0.37 | 0.86 |
| Tb | 0.54 | -0.01 | 0.06 | 0.70 | -0.02 | 0.33 | 2.13 | -0.06 | 0.83 |
| Dy | 4.51 | -0.10 | 0.09 | 4.85 | -0.12 | 0.36 | 11.88 | -0.32 | 0.83 |
| Ho | 1.31 | -0.03 | 0.13 | 1.25 | -0.03 | 0.38 | 2.76 | -0.07 | 0.82 |
| Er | 4.81 | -0.11 | 0.17 | 4.14 | -0.10 | 0.40 | 8.66 | -0.23 | 0.81 |
| Tm | 0.80 | -0.02 | 0.19 | 0.66 | -0.02 | 0.39 | 1.32 | -0.03 | 0.81 |
| Yb | 6.45 | -0.16 | 0.30 | 4.84 | -0.12 | 0.46 | 8.83 | -0.23 | 0.83 |
| Lu | 1.33 | -0.03 | 0.42 | 0.66 | -0.02 | 0.17 | 1.83 | -0.05 | 0.70 |
